# Sensational site: the sodium pump ouabain-binding site and its ligands

**DOI:** 10.1152/ajpcell.00273.2023

**Published:** 2024-01-15

**Authors:** Mordecai P. Blaustein, John M. Hamlyn

**Affiliations:** ^1^Department of Physiology, University of Maryland School of Medicine, Baltimore, Maryland, United States; ^2^Department of Medicine, University of Maryland School of Medicine, Baltimore, Maryland, United States

**Keywords:** cardiotonic steroids, digoxin, marinobufagenin, protein kinase cascade, signalosome

## Abstract

Cardiotonic steroids (CTS), used by certain insects, toads, and rats for protection from predators, became, thanks to Withering’s trailblazing 1785 monograph, the mainstay of heart failure (HF) therapy. In the 1950s and 1960s, we learned that the CTS receptor was part of the sodium pump (NKA) and that the Na^+^/Ca^2+^ exchanger was critical for the acute cardiotonic effect of digoxin- and ouabain-related CTS. This “settled” view was upended by seven revolutionary observations. First, subnanomolar ouabain sometimes stimulates NKA while higher concentrations are invariably inhibitory. Second, endogenous ouabain (EO) was discovered in the human circulation. Third, in the DIG clinical trial, digoxin only marginally improved outcomes in patients with HF. Fourth, cloning of NKA in 1985 revealed multiple NKA α and β subunit isoforms that, in the rodent, differ in their sensitivities to CTS. Fifth, the NKA is a cation pump and a hormone receptor/signal transducer. EO binding to NKA activates, in a ligand- and cell-specific manner, several protein kinase and Ca^2+^-dependent signaling cascades that have widespread physiological effects and can contribute to hypertension and HF pathogenesis. Sixth, all CTS are not equivalent, e.g., ouabain induces hypertension in rodents while digoxin is antihypertensinogenic (“biased signaling”). Seventh, most common rodent hypertension models require a highly ouabain-sensitive α2 NKA and the elevated blood pressure is alleviated by EO immunoneutralization. These numerous phenomena are enabled by NKA’s intricate structure. We have just begun to understand the endocrine role of the endogenous ligands and the broad impact of the ouabain-binding site on physiology and pathophysiology.

## Graphical abstract

**Figure:**
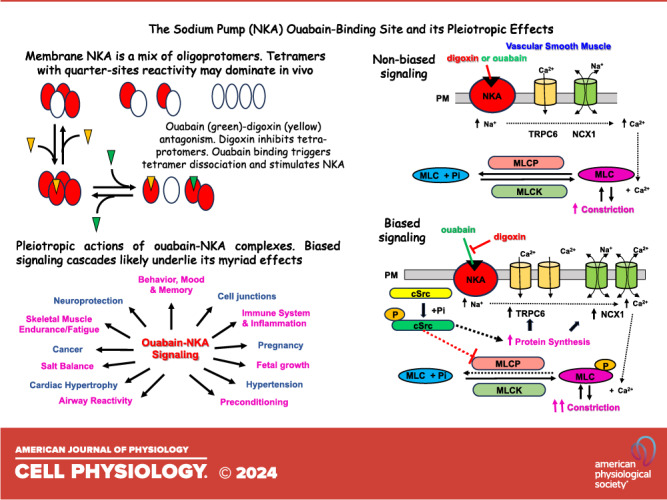

“I by no means expect to convince experienced naturalists whose minds are stocked with multitudes of facts all viewed, from a multitude of years, from a point of view directly opposite to mine… A few naturalists, endowed with much flexibility of mind… may be influenced by this volume; but I look with confidence to the future, -to young and rising naturalists who will be able to view both sides of the question with impartiality.”

Charles Darwin, *The Origin of Species by Means of Natural Selection* (1859), Chapter XV

“A new scientific truth does not triumph by convincing its opponents and making them see the light, but rather because its opponents eventually die, and a new generation grows up that is familiar with it.”

Max Planck, *Scientific Autobiography and Other Papers* (published posthumously in 1950).

## INTRODUCTION: THE PLASMA MEMBRANE SODIUM PUMP

The ubiquitously distributed plasma membrane (PM) sodium pump (Na^+^,K^+^-ATPase or NKA) is a fundamental component of virtually all animal cells and a marvelous molecular machine. It uses energy from ATP hydrolysis to transport Na^+^ out and K^+^ into cells and maintain intracellular low Na^+^ and high K^+^ concentrations, cell membrane potentials, and cell volume, as well as whole body Na^+^ homeostasis (1). The cation gradients provide the driving force for innumerable co- and countertransporters to move a large range of solutes and enable electrical signaling in nerve, muscle, and other cell types. The NKA accounts for ∼75–85% of ATP utilization in the brain and kidneys, and 33–40% of total body energy consumption but it is far more than a simple monovalent cation pump. It is the only known example in biology of a hormone receptor (the ouabain-binding site) located within a primary active ion transporter. In many cell types, the NKA is clustered into functional domains with glycolytic enzymes such that alterations in its activity are reflected by changes in local ADP production and, hence, glycolytic rates (2–5). This coupling allows the NKA to influence metabolism and underscores the links between the NKA and metabolically sensitive kinases; for example, it is involved in cardiotonic steroid (CTS)-induced activation of early-response genes (6) and several protein kinase (PK) cascades (7). It follows that ligands interacting with the ouabain-binding site will impact not only ion transport and signaling but also have more widespread influence on overall cell metabolism and function, gene regulation, and protein expression. In this way, the NKA and CTS play a role in such fundamental processes as cell proliferation and development, intercellular communication (“tight junctions”), and immune responses (8–14).

The NKA can perform these myriad functions because of its intricate, multifaceted structure and regulation summarized by the following six characteristics:

### The NKA Protomer

The NKA, which was discovered in 1967 (15), is a member of the P-type ATPase family because it forms a phosphorylated intermediate. It is an αβ protomer consisting of a catalytic (α) subunit and a small auxiliary (β) subunit (16). The α subunit contains all of the catalytic and cation transport machinery (17) as well as the CTS/ouabain-binding site (the subject of this review). The glycosylated β subunit is unique to K^+^-transporting P-type ATPases; it is an obligatory chaperone to α that can modulate the Na^+^ and K^+^ affinities (18–20).

### NKA Isoforms

Two NKA isoforms with markedly different affinities for ouabain were found in rodent brain and kidney in 1978–79 (21–23). Subsequent studies revealed that there are four NKA α isoforms (α1– α4) and three β isoforms (β1–β3) in most mammals (24). The composition of the αβ protomer can vary, but the physiological implications of the specific β isoforms, for example, α2β1 versus α2β2, are still poorly understood (16, 25). The affinities of α1–α4 for Na^+^ and K^+^ are tissue- and isoform-specific (26–30) and also depend upon the β isoform (31). All the α subunits of almost all mammals exhibit high affinity for ouabain [*K*_d_ ≤ 2–3 nM (8)] and other CTS. The rodent α1 isoform is a notable exception: this isoform has ∼1,000-fold lower affinity for CTS than the other isoforms in rodents (32) and all isoforms in other mammalian orders.

### NKA Isoform Expression and Distribution

Kidney epithelial cells express α1 NKA primarily, with only very small amounts of α2 and/or α3 (33–36). Most other cells express NKA with at least two different α isoforms (e.g., α1 and α2 or α1 and α3) that are differentially distributed in the cell membrane and have distinct functions (37–39) and distinct Na^+^ and K^+^ kinetics, perhaps modulated by the β isoform (20). Endothelia, glia, nonrenal epithelia, and all muscle cells (cardiac, skeletal, and smooth muscles), as well as some neurons express α1 and α2; many neurons, however, express α1 and α3 (24, 40). α1 is the predominant isoform (∼80–90% of total NKA) in most cell types. It is widely distributed in the PM and is often referred to as a “housekeeper” because it maintains the low cytosolic Na^+^ ([Na^+^]_CYT_) and high cytosolic K^+^ ([K^+^]_CYT_) concentrations. Exceptions include certain neurons that express only α3 (25, 41, 42), and skeletal muscle, where α2 predominates, but most of the α2 is usually located in sub-PM vesicles and can be translocated to the PM when activated, e.g., by insulin (43). α4 is expressed only in germ cells (28–30, 42).

### NKA Regulation by “FXYD” Peptides and by Protein Kinases and Phosphatases

The NKAs in myocytes, neurons, and kidney cells are regulated by seven small, single-membrane spanning “FXYD peptides” (so-called because of their signature sequence, phenylalanine-X-Y-aspartic acid) (44–47). FXYD peptides include FXYD-1, or phospholemman, which is expressed in all types of muscle (44, 46); FXYD-2, also called the NKA γ subunit, is expressed in kidney; and CHIF (corticosteroid hormone-induced factor) is expressed in the kidney and colon (48, 49). In many tissues, the NKAs are also regulated by certain PKs and phosphatases (50–53).

### NKA Regulation and Signaling by Endogenous (e)CTS

**Figure 1.**
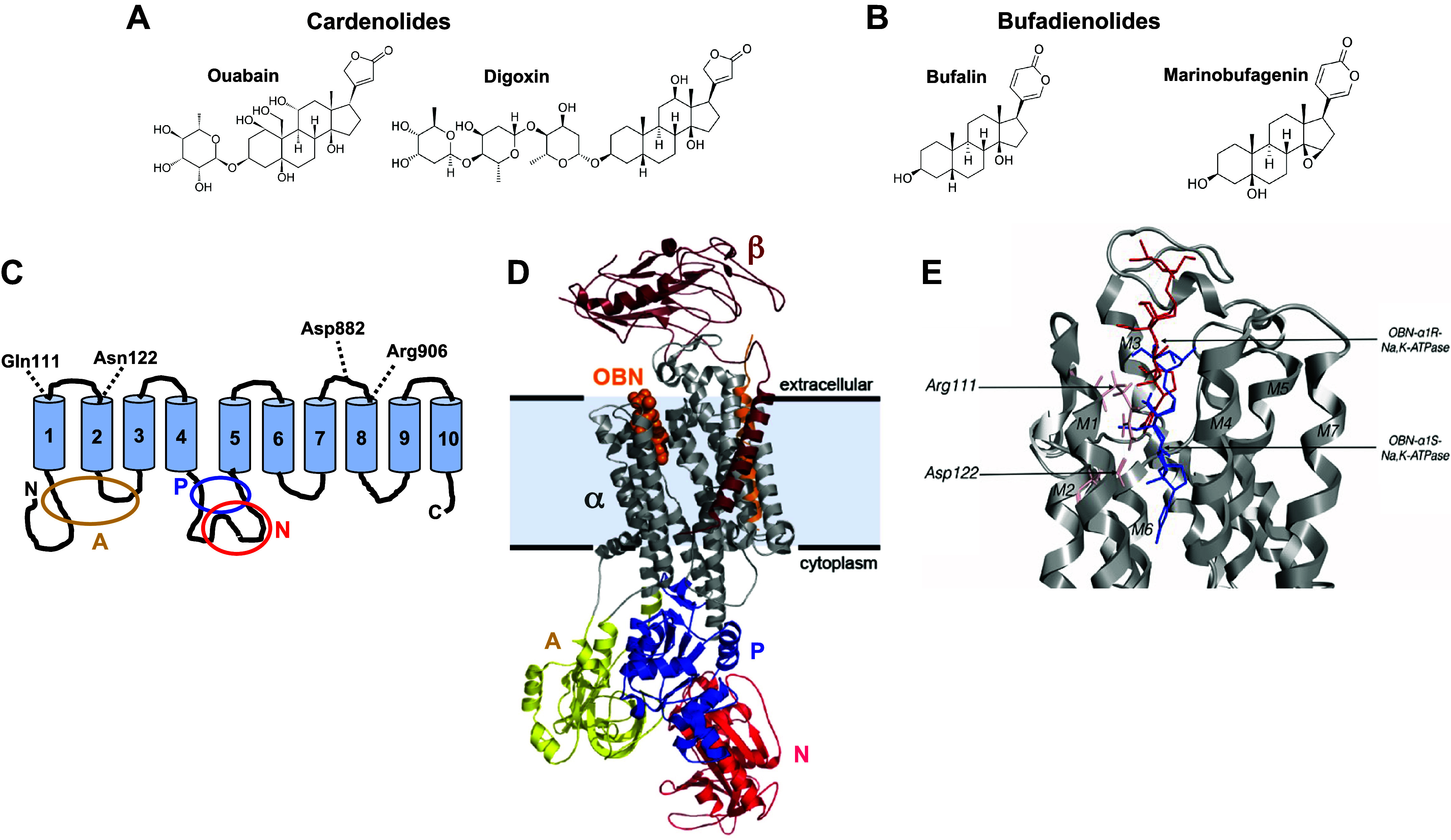
Cardiotonic steroids, NKA topography, and the NKA ouabain-binding site. *A* and *B*: the structures of two cardenolides (*A*), ouabain and digoxin, and two bufadienolides (*B*), bufalin and marinobufagenin, pertinent to this review, are illustrated. All four have both A/B and C/D rings in a cis conformation so that A and D are bent toward one another. Ouabain, digoxin, and bufalin are hydroxylated at C14, whereas marinobufagenin has an epoxide at C14–C15. *C*: diagram of the NKA α subunit topography showing the 10 transmembrane domains and the N and C termini. The colored ovals indicate the activator domain (A), spanning parts of the N terminus and the cytoplasmic M2-M3 loop, and the nucleotide-binding domain (red N) and phosphorylation domain (P), both spanning parts of the large M4-M5 cytoplasmic loop. The positions of the amino acids, Gln111, Asn122, Asp 892, and Arg906 are also indicated. *D*: structure of the NKA protomer in the E2P conformational state with bound ouabain (OBN, orange). This figure also shows the A domain (yellow), the N domain (red), and the P domain (blue) of the α subunit. Also shown are the β subunit (maroon), consisting of an extracellular (glycosylated) domain and a single transmembrane domain, and an FXYD peptide within the membrane (light orange). Reproduced from Ref. 54 with permission. *E*: detailed model structures of the pig kidney ouabain-sensitive (α1^S^) NKA ouabain-binding site with bound ouabain (blue), and the rat kidney ouabain-resistant (α1^R^) NKA with bound ouabain (red). Note that the two amino acid substitutions, Gln111Arg and Asn121Asp, enables the uncharged Arg and Asp to form a hydrogen bond and accounts for the ouabain resistance of rat α1. This bond prevents ouabain from penetrating as deeply into the pocket as it does in α1^S^, as illustrated. NKA, Na^+^, K^+^-ATPase (Na^+^ pump). Reproduced from Ref. 55 with permission.

NKAs are, in addition, regulated by endogenous steroid ligands that bind to the ouabain-binding site. This site is also the receptor for numerous exogenous, plant-derived, cardenolide CTS, and plant- and animal-derived bufadienolide CTS^1^ (Fig. 1, *A* and *B*) (56–58). One critical factor is the duration of CTS binding. Binding of CTS to NKA rapidly (seconds to a few minutes) blocks Na^+^/K^+^ transport and is cardiotonic (59) while prolonged CTS-NKA interaction (e.g., many minutes to hours, days, or longer) can activate various PK cascades (7).

### NKA Protomer Oligomerization

NKA αβ protomers are functional ATPases (60). These monoprotomers can also oligomerize to form functional diprotomers, (αβ)_2_, and tetraprotomers, (αβ)_4_ (60–64). Oligomerization apparently is required to explain certain CTS-NKA interactions (61).

*NKA Regulation and Signaling by Endogenous e(CTS)* and *NKA Protomer Oligomerization* are the primary issues explored in this review which, following introduction, is organized into three major sections, each with a number of subsegments. The first of these sections describes the origins of our knowledge about the CTS, their early uses, the detection of the CTS binding on NKA, and the discovery of endogenous CTS (eCTS). The next section summarizes the numerous surprises and complications encountered during the elucidation of CTS-NKA interactions, including the identification and characterization of CTS-activated cell signaling. We provide for the first time, an explanation for the stimulation of NKA activity by low-dose CTS and by antibodies raised against two extracellular loops of the NKA. The next section focuses on the eCTS-NKA endocrine system and the physiology and pathophysiology of CTS-NKA interactions with an emphasis on reports and reviews published in the past 6–7 years. Table 1 is a list of landmark observations about the NKA ouabain-binding site and its ligands; the table serves as a rough guide to the topics discussed here.

**Table 1.** Some of the milestone observations on the sodium pump ouabain-binding site and its ligands, the cardiotonic steroids relevant to this article

Year	Author	Event/Discovery	Reference
1532 BC	?	*Scilla* (squill) is mentioned in the Ebers Papyrus	(65)
∼600–900 BC	?	*Chan-Su* (toad skin extract containing bufadienlides) used as a therapeutic in China and Japan	(66)
∼1250	Physicians of Myddfai	Early therapeutic use of *Digitalis* in Wales described in the *Red Book of Hergest*	(67)
1542	Fuchs	Coined the Latin term, “Digitalis” (foxglove) in his herbal, *De Historia Stirpium Commentarii Insignes*	(68)
1785	Withering	Introduced *Digitalis* extract as a therapy for heart failure (“dropsy”) in *An Account of the Foxglove and Some of its Medical Uses*	(69)
1858	Kirk	Recognized that *Strophanthus kombé* seeds contain a substance that, like *Digitalis*, slows the heart	(70)
1872	Fraser	Extracted an active principle, “strophanthin,” from *Strophanthus hispidus* seeds and advocated for its use as a cardiotonic (1885)	(71)
1888	Arnaud	Crystallized ouabain (strophanthin-g) from the bark of *Acokanthera schimperi*, the “ouabaio tree”	(72)
1891	Fraser	Strophanthin is cardiotonic on frog heart (“increase of.. contractility”)	(70)
1930	Smith	Purified digoxin from *D. lanata*	(73)
1951	Maizels	During cold storage, cells lose K^+^ and gain Na^+^; after warming, Na^+^ is exported and Na^+^ is imported via “active transport”	(74)
1953	Schatzmann	Cardiotonic steroids inhibit the active transport of Na^+^ and K^+^	(56)
1953	Szent-György	In heart failure “we use (digitalis) to replace the missing screw” (the suggestion of an endogenous digitalis-like compound)	(75)
1956	Regan	The acute cardiotonic effect of acetyl strophanthidin is due to loss of K^+^ and gain of Na^+^	(76)
1960	Skou	Discovered Na^+^, K^+^-ATPase in crab nerve in 1957; in 1960 he showed that it was inhibited by ouabain	(77)
1960	Wilbrandt	Low doses of CTS can hyperpolarize frog cardiac muscle – possible evidence of NKA stimulation	(78)
1963	Repke	Low doses of CTS can stimulate the NKA and augment Na^+^ and K^+^ gradients	(79)
1969	Baker and Blaustein	Proposed that the acute cardiotonic effect of CTS is due to Ca^2+^ entry in cardiomyocytes mediated by Na^+^/Ca^2+^ exchange following NKA inhibition	(80)
1969	Dahl	Salt-sensitive hypertension can be transmitted from a hypertensive to a nonhypertensive rat by a circulating agent	(81)
1976-7	Haddy and Blaustein	Hypothesis that high dietary salt triggers secretion of a NKA inhibitor that is both hypertensinogenic and natriuretic	(82), (83)
1980	Thomas	Prescient modeling of the NKA ouabain-binding site	(84)
1980	Askari	Evidence that the NKA may operate as a tetraprotomer	(64)
1982	Hamlyn and Blaustein	The level of a circulating NKA inhibitor correlates with blood pressure (BP) in normotensive and hypertensive humans	(85)
1984	Huang and Smith	Anti-digoxin antibodies lower BP in aortic constriction hypertension	(86)
1985	Lingrel	Cloning of the NKA and elucidation of the α1 subunit isoform amino acid sequence. He later cloned and sequenced all 4 α and 3 β subunit isoforms	(87)
1985	Hamlyn	Suggests that stimulation of NKA by low ouabain concentrations may reflect displacement of an endogenous ligand	(88)
1988	Lingrel	Gln111 and Asn122 in the NKA α subunit M1-M2 extracellular hairpin loop are crucial for high ouabain affinity	(89)
1990	Goto	Analytical identification of a substance indistinguishable from digoxin in human urine	(90)
1991	Hamlyn and Blaustein	Purification of endogenous ouabain (EO) from human plasma and suggestion that it is synthesized in and secreted by the adrenal cortex	(91)
1992	Gottlieb	Plasma EO is elevated in many patients with heart failure and correlates inversely with cardiac output	(92)
1992	Wink	Asn122His substitution in the NKA α M1-M2 hairpin loop endows monarch butterflies with ouabain resistance	(93)
1993	Pamnani, Hamlyn, and Haddy	Prolonged administration of ouabain induces hypertension in rats	(94)
1993 and 2000	Hamlyn and Manunta	Ouabain-digoxin antagonism: ouabain, not digoxin, induces hypertension in rats; digoxin antagonizes ouabain’s hypertensinogenic effect	(95,96)
1993	Lichtstein	Bufadienolide, 19-norbufalin, identified in human cataractous lens	(97)
1993	Gann and Hamlyn	Ouabain is secreted by the adrenal gland in awake dogs	(98)
1994	Hamlyn	Ouabain is secreted by bovine adrenocortical cells in culture	(99)
1994	Leenen	Intracerebroventricular infusion of anti-digoxin Fab fragments, which bind ouabain, lower BP in Dahl salt-sensitive rats	(100)
1995	Rossi	Plasma EO is elevated in 45% of essential hypertensives and 56% of patients with adrenocortical adenoma	(101)
1996–9	Askari and Xie	NKA is a signal transducer: ouabain-binding activates early response genes and protein kinase (PK) signaling cascades, and increases reactive oxygen species (ROS)	(6, 102,103)
1997	Lingrel	Identification of all the amino acids in the NKA M1-M2 and M4-M8 regions that bind ouabain and influence its binding affinity	(104)
1997	Defaye	Bovine adrenocortical cells in culture secrete EO	(105)
1997	Hamlyn	Angiotensin II stimulates secretion of EO from bovine adrenocortical cells via angiotensin type 2 receptors	(106)
1998	Ferrari	Rostafuroxin, an ouabain antagonist, prevents the pressor effect of ouabain	(107)
2004	Grau	EO plays a role in osmoregulation in the euryhaline teleost, tilapia	(108)
2005	Lingrel	ACTH hypertension in mice requires an ouabain-sensitive α2 NKA	(109)
2005	Takahashi	Marinobufagenin and telocinobufagin identified analytically in human plasma	(110)
2005	Xu	Antibodies raised against the NKA M7-M8 extracellular loop triple NKA activity	(111)
2008	Deslongchamps	Total chemical synthesis of ouabain	(112)
2009	Toyoshima	Crystal structure of ouabain-bound NKA	(113)
2010	Aperia	Ouabain protects against rat fetus adverse renal development	(9)
2010	Bianchi	Ouabain antagonist rostafuroxin lowers BP in patients with hypertension with adducin- and ouabain-related gene variants	(114)
2012	Hamlyn	Plasma EO is elevated in pregnant rats which are ouabain-resistant	(115)
2013	Lingrel	Knockout of ouabain-sensitive α2 NKA in mice delays cardiac remodeling in aortic constriction-induced heart failure	(116)
2013	Golovina and Hamlyn	Ouabain-activated c-Src signaling upregulates Ca^2+^ transporters in ouabain hypertension; digoxin blocks the signaling.	(117)
2014	Leenen and Hamlyn	A novel hypothalamic slow neuromodulatory pathway, involving EO, plays a key role in the pathogenesis of salt-sensitive hypertension	(118)
2014	Hamlyn and Blaustein	Ouabain-digoxin antagonism requires NKA tetraprotomers with quarter-sites reactivity	(61)
2015	Lichtstein	Plasma EO is lower in pregnant human mothers of neonates with low-for-gestational-age birthweight than in mothers of normal birthweight neonates. Immunoneutralization of EO in pregnant rats causes cardiac enlargement and inhibits kidney and liver growth in rat pups.	(119)
2015	Nissen and Fedosova	Comparison of NKA α subunit crystal structures with ouabain, digoxin and bufalin bound	(120)
2021	Toyoshima	Comparison of NKA α subunit crystal structures with various cardenolides and bufadienolides bound	(121)
2021	Tverskoi and Lopatina	Different depth of insertion of different CTS into the binding pocket may determine differences in signaling and cytotoxic effects	(55)
2022	Toyoshima	Cryoelectron microscopy structure of NKA with ouabain bound shows conformational selection mechanism of ouabain binding	(122)
2023	Yang	EO suppresses immune response to non-small cell lung cancer and thereby promotes metastasis	(123)

## CARDIOTONIC STEROIDS AND THEIR RECEPTOR

### Origins: Digitalis

Herbs now known to contain CTS were used as medicinals by the ancient Romans, Syrians, and Egyptians. The ancients were aware of the diuretic and, perhaps, cardiac actions of extracts from the squill, *Drimia*^2^ *maritima*, which contain bufadienolide CTS including scillaren-A and bufalin (65), even though the therapeutic effects of these preparations were not systematically investigated. Perhaps the earliest written account of the medicinal use of squill is in the Ebers Papyrus of 1532 BC (124). The traditional Chinese medicine, *Chan Su* (*Senso* in Japan), prepared from dried skin gland secretions of the Chinese toad, e.g., *Bufo melanostictus*, contains bufalin (Fig. 1*B*) and other bufadienolides; it is used to treat cardiac and other ailments (66, 125).

Digitalis was reportedly described as early as 1250 (126) and was prescribed in the 13th–14th centuries by a Welsh family known as the Physicians of Myddfai whose medical writings are included in the *Llyfr Coch Hergest* (*Red Book of Hergest*, 1375–1425) (67). Leonhart Fuchs (1501–1566) coined the Latin name, *Digitalis*, as a translation of the German word for thimble, *fingerhut*, which he used to describe the shape of the flower in his herbal, *De Historia Stirpium Commentarii Insignes* (1542) (68); he even suggested that it was useful for the treatment of “dropsy” (edema). Digitalis, known as “foxglove” in English, was also described in the English herbals of John Gerarde (*The Herball or General Historie of Plantes*, 1597) and John Parkinson (*Theatrum Botanicum*, 1640) and was included in the *London Pharmacopoeia* of 1661. Nonetheless, digitalis was only used sporadically as a therapeutic agent because of its toxicity and the lack of a systematic trial.

The situation changed dramatically in 1785, when William Withering published his monumental monograph, *An Account of the Foxglove and Some of Its Medical Uses* (69). During the prior decade, Withering, who was much more than just an astute passive observer, documented the effects of an extract of *Digitalis purpurea* leaf on 163 patients. This was, of course, before the invention of the stethoscope (1816), sphygmomanometer or EKG, and prior to modern medicinal chemistry and clinical trials! *1*) Withering found, presumably by experimentation, that digitalis was the active principle in a brew of some “twenty or more different herbs” (69). *2*) He reported that “the diuretic effects (of digitalis)… do not depend on its exciting a nausea or vomiting” – i.e., low, non-toxic doses are effective. Diuresis was his primary end point, and he considered digitalis “the most certain diuretic I know.” *3*) He clearly recognized digitalis’ cardiotonic action: “it has a power over the motion of the heart, to a degree yet unobserved in any other medicine that… may be converted to salutary ends.” *4*) In his Preface, Withering stated that, “it would have been an easy task to have given select cases… But Truth and Science would condemn the procedure… I have therefore mentioned every case in which I have prescribed the Foxglove…, successful or otherwise… I seldom prescribed (digitalis), but when the failure of every other method compelled me to do it; so that upon the whole, the instances I am going to adduce, may truly be considered as cases… only snatched from destruction, by the efficacy of the Digitalis” (69). Withering provided sufficient information from 152 of his cases for a cardiologist to infer a diagnosis of either cardiac or other (noncardiac) causes of dropsy; 44 were “most likely to have been in heart failure” (HF) (127). In the latter, the “success rate” of digitalis therapy, i.e., induction of diuresis, was 89%; the overall success rate was 64% in all 152 cases (127).

Within a year of the book’s publication, Hall Jackson, a New Hampshire physician, requested digitalis seeds from Withering and introduced digitalis into clinical practice in America (128). Based on its salutary effects, digitalis became the mainstay of HF therapy for the next two centuries even though the first of its active cardenolide components, digoxin (Fig. 1*A*), was not isolated until 1930 (73). The large clinical trial in the mid-1990s, however, led the Digitalis Investigative Group (DIG) to conclude that digoxin’s effects in HF were very modest: it “did not reduce overall mortality, but… reduced the rate of hospitalization” (129). Consequently, prescription of digoxin declined markedly, especially in the United States, as other, effective therapies for HF emerged. This rejection of digoxin therapy may have been premature. Reanalysis of the DIG trial data and some small trials suggest that the lowest doses of digoxin may, in fact, be more efficacious than higher doses, e.g. Refs. 130–134; a new interpretation (below) of the long-unexplained observation that low concentrations of ouabain can stimulate the NKA may explain why. Furthermore, landmark discoveries (1990s–present) about endogenous CTS, CTS-activated cell signaling, and ouabain-digoxin antagonism, described in this review, also suggest that digoxin’s therapeutic potential merits reevaluation (135).

### Enter Ouabain

In 1858, John Kirk joined David Livingstone’s expedition to the Zambezi River, Africa, as its botanist and physician. Upon learning about the natives’ use of *Strophanthus kombé* seeds for poison arrows to kill animals as large as a buffalo, Kirk, himself, collected *kombé* seeds (70) and put them in his shirt pocket where he also kept his toothbrush. After brushing his teeth, he recognized that his pulse rate soon slowed and that this effect of *kombé* seeds seemed very similar to that of digitalis (136). In what is surely a remarkable example of independent, parallel discovery, native South American tribes also use extracts of plants from the genus *Naucleopsis*, that contain *Strophanthus* steroids, in poison blow darts (137).

Thomas Fraser extracted “a very powerful active principle” from the seeds of the closely related species, *Strophanthus hispidus*, in 1872. He named this principle, “strophanthin” (138), his extracts contained strophanthin-k and several other strophanthins, which are cardenolide CTS. He showed that a high (lethal) dose of strophanthin acts directly on the frog heart to “increase… its contractility, rendering systole more prolonged and perfect” (i.e., it is “cardiotonic”). When injected into the carotid arteries of rabbits, strophanthin slowed the pulse, increased pulse amplitude, and transiently elevated blood pressure (BP); the rabbits died in cardiac systole (70). Fraser advocated for the clinical use of strophanthins as cardiotonic agents (71). Even recently, a few investigators continue to promote ouabain (aka, strophanthin-g) as a therapy for HF (139), but others have stressed its toxicity (140).

Leon-Albert Arnaud crystallized the arrow poison, ouabain (Fig. 1*A*), from the bark of the Somali “ouabaio tree,” *Acokanthera schimperi*, in 1888 (72). Ouabain can also be obtained from the woody shrub, *Strophanthus gratus*, hence the name, strophanthin-g. More than a century later, Pierre Deslongchamps and colleagues (112) achieved the total chemical synthesis of ouabain and its aglycone, ouabagenin. Because of its relatively high solubility in aqueous media [1 g dissolves in 75 mL of water (141)], ouabain remains the investigators’ CTS of choice for identifying NKA and studying the physiological effects of NKA inhibition, e.g., Refs. 142 and 143].

### CTS as an Animal Defense Mechanism

Several insect groups have independently evolved strategies for sequestering or elaborating CTS to defend themselves (144). These include Danian butterflies, exemplified by the monarch, *Danaus plexippus,* and the milkweed bug, *Oncopeltus fasciatus*. Milkweed is a favorite food of the monarch, an important plant pollinator, even though milkweed flowers and leaves contain an abundance of cardenolide CTS (145). The butterflies feed on the milkweed nectar and deposit their eggs on the leaves, which the larvae then eat, and both larvae and adults sequester the consumed CTS. The sequestered cardenolides then serve as a defense, protecting the monarchs from predatory birds. Indeed, female, but not male, Mexican pine white butterflies, which do not sequester CTS, mimic the coloring of the monarchs, and thereby also avoid avian predators (146). But how does the monarch, which expresses NKA in all its cells, tolerate the toxic cardenolides? Answer: its NKA sequence^3^ has evolved with two key amino acid (AA) substitutions in the ouabain-binding pocket, those at positions 111 and 122 at the beginning and end, respectively, of the first external hairpin loop (M1-M2; Fig. 1*C*) that greatly reduce CTS sensitivity (93, 147, 148). This enables the monarch to avoid the inconvenience normally associated with a state of perpetual poisoning.

Despite 300 million years of insect evolutionary divergence, the identical molecular strategy is used by several different orders of insects that ingest CTS (148). But, is high ouabain sensitivity then irrelevant? Studies on the milkweed bug which, as the name implies, feeds on milkweed seeds, suggest that both high and low ouabain sensitivity have context-dependent relevance exemplified by the fact that this insect has evolved triplicate NKA genes, α1A, α1B, and α1C (149). The ouabain-sensitive α1C NKA is highly expressed in neural tissue and is believed to be important for neuronal excitability; knockdown of this isoform is lethal. In contrast, the more ouabain-resistant α1A and α1B, are primarily expressed in the periphery, outside the blood-brain barrier, especially in the Malpighian tubules that excrete the ingested CTS, thereby helping to protect milkweed bugs from the ingested toxin (149).

Fireflies, familiar luminescent beetles of the genus, *Photinus*, use an analogous strategy to protect themselves from vertebrate predators: their tissues contain several bufadienolides termed lucibufagins (150, 151). Whether these fireflies synthesize the lucibufagins from ingested cholesterol, or feed on bufadienolide-synthesizing plants is unknown. Fireflies of the related genus, *Photuris*, also contain lucibufagins but do not generate their own. Instead, female (“femmes fatales”) *Photuris* fireflies, by mimicking *Photinus* females with their flash timing, lure *Photinus* males, and catch and devour them, thereby enriching themselves with lucibufagins (152). Both firefly genera have evolved bufadienolide-resistant mutations in their NKA α subunits through gene duplication (153).

East African crested rats, too, cleverly use CTS for a defense mechanism, but they use a different strategy. These fairly large rodents (adults weigh ∼700–900 g) chew on the bark of the ouabaio tree and then deposit the masticate containing ouabain and other cardenolides onto specialized, hollowed-out hairs along their flanks that behave like wicks (154). When alarmed, the crested rats retract their heads and flare out their flanks containing these poisonous hairs to ward off predators: attackers can be gravely harmed or killed by an encounter (155). A disadvantage of ouabain and other *strophanthus* CTS is that, because they are polar and hydrophilic, they are relatively poorly absorbed from the gastrointestinal (GI) tract (156). To ward off predators, they therefore must be introduced directly into the skin or circulation to be effective. This property contrasts with the hydrophobic *Digitalis* and bufadienolide steroids that are readily absorbed when introduced orally (157).

Another twist on the chemical defense story is the case of the bufonid (“true”) toads of the *Bufonidae* family that protect themselves with bufadienolide CTS. In this case, however, the toads synthesize their own toxins and secrete them from skin glands (158). As mentioned in *Origins: Digitalis*, Chan Su is extracted from dried *Bufo* skin glands. The bufonid toads are protected from their own toxins by an ouabain-resistant α1 NKA (159), but not from certain taxa of snakes, e.g., the Japanese natricine subfamily of colubrid snakes, which are resistant to bufadienolides. These snakes safely feed on the toads and then sequester the bufadienolides as a defense mechanism (160–162), analogous to the monarch butterflies and cardenolides. Similarly, Asian and African varanid lizards feed on bufonid toads and are resistant to their toxins (163). Resistant snakes and lizards also have AA substitutions, e.g., Q111L and G120R, in the M1-M2 loops of some of their NKA isoforms (162, 163), especially the isoform expressed in the heart (161). In a further twist, Australian varanid lizards, which evolved in a bufonid-free environment, do not have those mutations, but the lizard mortality rate rose rapidly after the tasty South American cane toad, *Bufo marinus*, was introduced (163).

### The Na^+^ Pump is the Ouabain Receptor

#### Discovering the CTS/ouabain receptor.

The history of the discovery of NKA was recently reviewed (1) and will not be recapitulated. Nevertheless, a few milestones are particularly relevant to the present discussion. In 1939, August Krogh wrote that “steady expenditure of energy in special mechanisms” was required to maintain osmotic balance between cells and their environment (164). Two years later, Dean proposed that “there must be some sort of (ion) pump… in the (cell) membrane, which can pump out sodium, or, what is equivalent, pump in potassium” (165), i.e., a “sodium pump.” Harris (166) and Maizels (74) established that cold storage or glucose removal causes human erythrocytes to lose K^+^ and gain Na^+^, and that Na^+^ is exported and K^+^ is imported by “active transport” (74) when the cells are warmed and glycolysis is restored.

Hans Jürg Schatzmann’s seminal 1953 study directly linked CTS to what would be subsequently recognized as the NKA ouabain-binding site. As he later noted, based on somewhat naïve reasoning, he tested the idea that mineralocorticoids might provide the ionophoric group and, thereby, directly stimulate Na^+^ extrusion from erythrocytes, but the results were negative. Then, thinking that another steroid might work (167), he tested *Strophanthus* and *Digitalis* CTS, specifically, strophanthoside-k and the aglycones, strophanthidin and digitoxigenin, and thus, serendipitously discovered that they all inhibited the active transport of Na^+^ and K^+^ (56). That same year, Hajdu (168) reported that the cardiotonic effect of high concentrations of digitalis on the frog heart was associated with the loss of K^+^ from the heart. More convincingly, in 1956, Regan and colleagues (76) demonstrated, in acute experiments, that enhanced cardiac performance due to high concentrations of acetyl strophanthidin was associated with a gain of Na^+^ as well as a loss of K^+^ by the dog heart.

In 1957, Jens Skou discovered a Na^+^- and K^+^-dependent ATPase, or NKA, in crab nerve membranes (15). A year later, he was visited by Robin Post, who asked him if he had tested ouabain on the preparation. Skou responded, “What’s ouabain?,” so Post described to him Schatzmann’s discovery that CTS inhibit active Na^+^/K^+^ transport.^4^ Consequently, in Skou’s second (1960) article on the cation-dependent ATPase, he showed that it was, indeed, inhibited by ouabain, thereby confirming that NKA was the CTS/ouabain receptor and was linked to the active transport of Na^+^ and K^+^ (77).

#### How does NKA inhibition by CTS lead to cardiac inotropy?

The aforementioned results raised a crucial question: How does NKA inhibition enhance cardiac contraction? Experiments performed simultaneously and independently in Mainz, Germany, and Plymouth, UK, in the late 1960s provided an answer (169, 170). Harald Reuter, who had just learned about Na^+^/Na^+^ exchange diffusion at a short course on Laboratory Techniques in Membrane Biophysics (169), postulated an analogous Na^+^/Ca^2+^ exchange (NCX) in the heart. By measuring ^45^Ca efflux, he successfully demonstrated NCX in guinea pig cardiac muscle (171). Simultaneously, the Plymouth group, while studying NKA kinetics, serendipitously stumbled onto the NCX in squid giant axons (80, 172). They explicitly proposed that NCX was the missing link that explained how NKA inhibition, by raising the cytoplasmic Na^+^ concentration ([Na^+^]_CYT_), indirectly elevated cytosolic Ca^2+^ ([Ca^2+^]_CYT_) and thereby enhanced contraction (80). This concept was confirmed when Ca^2+^ measurements with the Ca^2+^-activated photoprotein, aequorin, became available (59), and it is now widely accepted (173). As we shall see, however, the CTS-NKA interaction is far more complicated than this straightforward mechanism implies. Indeed, there is evidence that low CTS concentrations can be cardiotonic without lowering the transmembrane Na^+^ and K^+^ gradients.

This view of NKA-NCX functional cooperation in the cardiotonic action of CTS was extended to include the vasotonic action of CTS with the discovery of NCX in vascular smooth muscle (VSM) (174).

The latter report noted that this functional coupling might be important for the maintenance of vascular tone and suggested that, because of a possible tendency toward an elevated [Na^+^]_CYT_, this mechanism might be involved in the pathogenesis of hypertension. Before exploring this topic further, however, we need to review the current state of knowledge about CTS and the NKA ouabain-binding site.

#### The ouabain-binding site is promiscuous and its ligands are numerous.

Over 400 naturally occurring and synthetic cardiac-active steroids were already recognized in 1970 (e.g., Refs. 175 and 176). Today, the number likely exceeds 1,000, reflecting new discoveries and the ever-increasing number of synthetic derivatives (e.g., Refs. 177–179). That all of these ligands can interact with the ouabain-binding site on NKA, albeit with varying affinities, is a remarkable biological phenomenon. For example, contrast the behavior of the NKA CTS binding site with its analog on α- and β-adrenergic receptors. In the latter examples, the number of ligands, synthetic or otherwise, is miniscule by comparison, and the receptors are highly selective for a very restricted number of structurally related ligands (180). Why then are there so many ligands for the ouabain-binding site? And, does the promiscuity of this binding site have any biological significance? We suggest that latent promiscuity ordinarily is not an issue if the number of available partners is limited.

Restricting the discussion to mammals, promiscuity of the ouabain-binding site is likely of minimal consequence. In humans, it is probably limited by the four or possibly five endogenous CTS (eCTS) entities detected in the circulation; these are the most biologically active in terms of their ability to inhibit ion transport by NKA (181) although they may have diverse effects on signaling at much lower concentrations. These eCTS include some of those shown in Fig. 1, *A* and *B*. However, latent promiscuity of the CTS binding site can be impacted by certain plant or animal materials containing high concentrations of exogenous, structurally related CTS that may be ingested either by accident or design. Lower, typically undetected, levels of various CTS are almost certainly routinely present in the food chain of humans and laboratory animals (182–186). The exogenous CTS, most of which are readily bioavailable, will be absorbed and have the potential to compete with eCTS for binding to NKA. Depending on their respective amounts, association and dissociation rates, and the structural families to which they belong, they may be able to influence signaling events ordinarily mediated by eCTS (see *The CTS-Activated “Signalosome”*).

### The Idea of a Circulating Ouabain-Like Compound: Possible Clinical Implications

Albert Szent-Györgyi, in his 1953 monograph on muscle contraction, postulated that, “if we saved a (failing) human heart with a decoct of foxglove… we… use it (Digitalis) to replace the missing screw” (75). This was, perhaps, the first hint of a functionally relevant endogenous digitalis-like substance (eDLS). A decade later, Louis Dahl and colleagues developed two genetic strains of rats with different disposition to salt-dependent hypertension, a salt-sensitive strain and a salt-resistant strain (187, 188). In experiments on a rat from each of these strains united in parabiosis, high dietary salt-induced hypertension could be passed from the sensitive to the resistant rat by a circulating agent (81, 187). Converging ideas about a hypertensinogenic and natriuretic hormone, a NKA inhibitor, were then pursued by numerous investigators (82, 189–196). This culminated with the hypothesis that excess dietary salt leads to the secretion of a humoral NKA inhibitor, an endogenous ouabain-like compound (eOLC) that, paradoxically, may be both natriuretic, due to its inhibition of renal Na^+^ reabsorption, and hypertensinogenic, due to its vasotonic action (83). In retrospect, as we shall see, this idea was prescient but far too simplistic. Nevertheless, in 1982, we discovered that the level of NKA inhibitory activity in the plasma correlates with the BP among normotensive humans and humans with hypertension (85). This fueled the hunt for the elusive endogenous NKA inhibitor(s), or eOLC(s), a subject to which we will return shortly.

### Cloning the NKA and Identifying the Ouabain-Binding Site

On the bicentenary of Withering’s seminal report on the foxglove (69), Lingrel and colleagues (87) revolutionized the NKA field by elucidating the AA sequence of the sheep kidney (α1) NKA catalytic subunit, a first. Lingrel’s group subsequently sequenced the other three mammalian NKA α subunit isoforms, α2–α4, and the three β subunit isoforms, β1–β3 (30, 197). They described the membrane topology of NKA: 10 hydrophobic transmembrane segments, M1–M10, with the N- and C- termini in the cytoplasm (Fig. 1*C*). They also identified three AAs crucial for cation binding in helices M5 and M6 (198, 199), and the ATP binding site; the latter is situated in the large cytoplasmic loop between M4 and M5, in close proximity to the cation binding sites (197).

Lingrel and colleagues used a combination of mutagenesis and expression studies to identify the α subunit AAs that bind ouabain (104, 200, 201). They showed that two regions of the NKA α subunit are involved in ouabain binding: transmembrane regions M1 and M2 and the extracellular loop joining them, and the extracellular and transmembrane portions of transmembrane segments M4–M7 (89, 104, 199, 201). The regions of cation binding and ouabain binding overlap in helices M4 and M5. Subsequent structural studies confirmed that the AAs that are critical for CTS binding are located in the extracellular-facing vestibule formed by the outer portions of the α subunit M1, M2, and M4–M7 transmembrane helices (Fig. 1, *D* and *E*) (120, 121, 202). The M4–M7 region also comprises the cation binding sites involved in Na^+^ and K^+^ translocation; Na^+^ exits to, and K^+^ enters from, the extracellular fluid via the vestibule when it is not blocked by CTS. Lingrel et al. (89) identified Gln-111 and Asn-122, located in the M1-M2 external loop adjacent to the M1 and M2 transmembrane α helices, respectively, as especially important for determining ouabain sensitivity. Substitution of either positively or negatively charged amino acids for these two uncharged AAs, as in the rodent α1 isoform, renders the NKA resistant to ouabain (197). Note that these two AA residues are in the very same positions which, when substituted through evolution, account for the resistance of certain insects to cardenolide CTS (see *CTS as an Animal Defense Mechanism*) (147).

The NKA α subunit is an ancient protein that has its origins in Archea, fungi, and bacteria; it evolved into a multifunctional P-type ATPase beginning with the paramecium, a unicellular protozoan (203, 204). The α subunit protein in the paramecium has substantial topological similarity to that of hydra, frogs, and humans, with nine transmembrane α-helical segments and two half-helices, and with cytoplasmic N and C termini (204). There is modest sequence similarity between the paramecium and human α subunit transmembrane segments, but substantial sequence similarity among the hydra, nematode, fruit fly, frog, and human NKA α subunits (204–206). The invertebrate β and human β subunit sequences are, however, more distinct (205).

The ouabain-binding site appeared early in the evolution of the α subunit in invertebrates (205): ouabain inhibits NKA in hydra, nematodes, cephalopods, and insects (143, 205, 207, 208). Clearly, NKA ouabain-binding activity has been exceedingly well-conserved throughout animal evolution. The significance of this site in the invertebrate and lower vertebrate NKAs is unknown, but the evolutionary conservation of the ouabain-binding site is surely noteworthy. With hindsight, this would seem to be strong circumstantial evidence for the presence of an endogenous ligand (or ligands) even in invertebrates. In the euryhaline teleost, tilapia (*Oreochromis mossambicus*), an eOLC may play a role in the osmoregulatory response to changes in water salinity (108). When fresh water-adapted fish were transferred to salt water, there was a significant positive correlation between plasma osmolality and plasma ouabain immunoreactivity, suggesting that, perhaps, the eOLC helps to enhance NaCl excretion. When, during evolution, did the eOLC-NKA couple and eOLC-activated cell signaling appear? It should be very instructive to learn about the origins of this couple as a hormone-receptor complex.

### The Hunt for the Holy Spirit

As noted (*The Idea of a Circulating Ouabain-Like Compound: Possible Clinical Implications*), the correlation between a circulating NKA inhibitor and BP in human subjects (85) fostered a competitive search for the eOLC(s) (83, 170). In 1991, we and our colleagues at the Upjohn Company purified one of four bioactive NKA inhibitors from human plasma and reported its identity as EO, using a combination of analytical methods including mass spectroscopy (MS). EO is endogenous in humans and rats and the bulk of its circulating level is dependent on the adrenal cortex (91, 209). While welcomed by many (210–215), this new hormone discovery was criticized by a few skeptics who claimed to be unable to detect the hormone (216–219). Two of these groups were adamant: “ouabain is unlikely to be an endogenous mammalian cardiotonic steroid” (216) and “endogenous ouabain is not ouabain” (220). Other critics simply cited the preceding authors (221) or raised the possibility of contamination by commercial (plant) ouabain (222).

Three of the four groups reporting negative results used anti-ouabain or anti-digoxin immunoassays to measure EO (216, 217, 219). In contrast, another group using an ELISA (enzyme-linked immunosorbent assay) generated with anti-ouabain antibodies that exhibited less than 1% cross-reactivity with non-*Strophanthus* steroids (223), detected EO in human plasma and cerebrospinal fluid and in the plasmas of several other mammals (224). Numerous other groups have also successfully used immunoassays based on ouabain-selective^5^ antibodies to measure EO in human plasma (e.g., Refs. 225–229), and in the plasma, tissues, and cells of other mammals (e.g., Refs. 230–232). Repeated reference (e.g., Ref. 221) to the same three negative immunoassay studies from the 1990s seems unjustifiable in light of these many positive reports.

More recently, “ultrasensitive” tandem liquid chromatography-MS (LC-MS) has been used by three groups; one has claimed that EO “is not detectable in human plasma” (218), but that study has crucial flaws. The investigators admitted that “inconsistency was introduced by editing the… raw data” (233). They neither acknowledged nor explained the fact that EO was detected in their human plasma samples by an MS-validated, ouabain-selective radioimmunoassay (RIA) (234). Most importantly, that study (218) omitted key steps needed to validate the results.^6^ As stated in a recent review (235), “Disqualifying 30 yr of work with a single study in 30 patients… (i.e., (218)), seems premature.” In contrast to the study by Baecher et al, another group detected 10 distinct eCTS in human plasma including EO as part of the RATE-AF trial. However, the measured concentrations were dramatically lower than those previously reported by immunoassays (236). Furthermore, in that trial, the baseline levels of these eCTS did not influence the beneficial therapeutic effect of digoxin. In contrast to the abovementioned studies, a third group described circulating levels of EO as determined by LC-MS-MS that overlapped with radioimmunoassay values (123). It is not clear why these supposedly gold-standard analytical systems yield such differences.

The original identification of the purified ouabain in human plasma by MS (91, 209) was not questioned (the MS spectra are undeniable) and the methods were not criticized. Rather, the possibility that this substance might be a laboratory contaminant from a plant source (e.g., commercial ouabain) was implied or even asserted (222). Concerns about the possible sequestration of ouabain from the diet (213, 216) seem far-fetched because of ouabain’s low bioavailability (237) and lack of apparent plant-related dietary sources. Nevertheless, many animal tissues and meat products consumed by humans do contain eCTS. Typically, the tissue levels, especially for EO in muscle and, with the exception of the adrenal glands, pituitary, and some regions in the brain (not ordinarily consumed), are probably too low to act as meaningful sources. Furthermore, all these concerns were addressed by the detection of ouabain in the plasma of subjects on total parenteral nutrition (91).

Skepticism was expressed about the ability of mammals to synthesize the stereochemical features of the steroid nucleus found in some plant CTS. There was concern that the A and B, and C and D rings are both in cis rather than trans conformation so that rings A and D face one another, and that the carbon at position 14 (C_14_) is hydroxylated because these are not characteristic of common mammalian steroids (213). But this is not evidence that EO cannot be a mammalian steroid. These are structural requirements for both cardenolide and bufadienolide cardiotonic steroids that inhibit vertebrate cardiac muscle NKA with high affinity (238). Furthermore, bufadienolides are synthesized from cholesterol in the parotid and skin glands of bufonid toads (239, 240) so it is entirely reasonable to anticipate that mammals, too, may be able to synthesize CTS with cis-fused rings and a β-hydroxylated C_14_.

Critics ignored the fact that the scaled-up extraction from 300 L of human plasma, and the purification and analysis in the initial studies, were all performed at a large ethical pharmaceutical firm, the Upjohn Company (181, 209), where quality control was standard. The isolation yielded 31 µg of pure product, sufficient for functional testing and immunoassay (181). This corresponds to an original starting concentration of 177 pM in the native plasma that, corrected for ∼50% loss during the Na^+^, K^+^-ATPase enzyme affinity step, results in a concentration of ∼354 pM, well within the normal range of EO measured in multiple clinical studies by immunoassay (241–243). Laboratory contamination by exogenous ouabain was, therefore, quantitatively unlikely. In contrast, all subsequent attempts to purify eCTS from mammals to date have used dramatically smaller amounts of plasma or tissue so that only trace quantities of products would be expected. Furthermore, the carbon ratio, C^12^/C^13^, helps distinguish substances of plant and animal origin, and high-resolution MS indicated that the C^12^/C^13^ ratio of bovine adrenal EO was significantly lower than that of commercial (plant) ouabain (244, 245). Thus, adrenal EO was not a laboratory contaminant.

Others expressed doubts about the ability of mammals to synthesize and utilize the sugar, rhamnose (ouabain is a rhamnoside: Fig. 1*A*) (216), but the evidence for rhamnose metabolism in mammals and other animals was overlooked. Rhamnose is present in rat brain mitochondria (246); it is metabolized in pigs, primarily in the adrenals, hypothalamus, and pituitary (247, 248); and it is coupled to hormones such as gonadotropins in pregnant mares (249). The nematode, *Caenorhabditis elegans*, can synthesize l-rhamnose (250) as can rabbit skin (251). Furthermore, this sugar is abundant in animal diets, e.g., it is a key component of plant cell walls (252). The availability of l-rhamnose for ouabain biosynthesis is, therefore, a nonissue even though its metabolism in mammals is not well understood.

EO has now also been identified analytically in numerous other laboratories by MS and/or nuclear magnetic resonance (NMR), e.g., in human plasma (91, 123, 209, 241, 253), in bovine adrenals (105, 231) and hypothalamus (254), and in rat and mouse plasma (244) and rat urine (255). Moreover, in addition to EO, at least two ouabain isomers, detected as their lithium adducts using offline LC-MS-MS-MS and absent from commercial ouabain sources, have been detected in rat plasma (115, 118). Adrenal biosynthesis has been verified even though only part of the biosynthetic pathway has been elucidated (8, 98, 99, 105). EO tissue distribution, adrenal versus systemic vein EO measurements, incorporation of precursors, and tissue culture and adrenalectomy studies all indicate that the adrenal cortex is a major source of circulating EO (91, 98, 99, 256, 257). Both adrenocorticotropic hormone (ACTH) and angiotensin II (Ang II) stimulate EO production by adrenocortical glomerulosa cells in primary culture (106, 258–261). Possibly relevant to EO biosynthesis, normal rats infused with anti-ouabain antibodies for 4 wk develop adrenal hypertrophy but unaltered plasma aldosterone and corticosterone levels (223), consistent with an attempt by the adrenals to augment EO biosynthesis and secretion specifically. Furthermore, Hamlyn and co-workers described a new syndrome (“ouabainoma”) with poorly controllable hypertension, an adrenocortical adenoma, and elevated plasma EO but normal plasma renin activity (PRA), aldosterone, and cortisol levels. Following adrenalectomy of the adenoma-bearing gland, BP and plasma EO declined and hypertension became manageable with a single drug. Most importantly, the adenoma tissue exhibited a very high level of EO (262). Subsequently, Naruse and colleagues (256) reported another possible case of this syndrome, a patient with severe hypertension, an adrenal adenoma, and elevated plasma OLC in the adrenal vein on side of the adenoma tumor, but normal PRA, aldosterone, and cortisol.

There is also convincing evidence for local EO biosynthesis in the brain. Ouabain immunoreactivity has been detected in the hypothalamus (263), small amounts of OLC immunoreactivity are secreted by hypothalamic cells in vitro in an aldosterone-dependent fashion (232), and hypothalamic tissue EO has been verified by MS (254). In addition, intracerebroventricular (ICV), but not intravenous (IV), infusion of a low dose of benzamil, an epithelial Na^+^ channel (ENaC) blocker, prevents the increase of OLC in the hypothalamus and pituitary, and the BP elevation, in Dahl salt-sensitive (S) rats on a high-salt diet (264). Furthermore, ICV low dose mineralocorticoid receptor blockers (e.g., eplerenone) and Ang II receptor antagonists (e.g., losartan), as well as benzamil, all prevent the increase of OLC in the hypothalamus and pituitary, and the BP rise in central aldosterone-induced hypertension (265, 266) (see *The NKA Ouabain-Binding Site-EO-BP Connection*).

One skeptic (221) not only questioned the identification of EO, but even raised doubts about the existence of an endogenous CTS: “What is puzzling… is that so many… investigators doing… research on the sodium pump or digitalis have bought into the proposal that there must be ‘digitalis‐like hormones’.” Astonishingly, this view ignores all the available ancillary evidence, especially the dramatic functional consequences of more or less obliterating the ability of the NKA ouabain-binding site to bind CTS, endogenous or otherwise. The functional consequences of these manipulations in various models of hypertension and heart failure, along with the impact of immunoneutralization of eOLCs cited below, are remarkable (see *The NKA Ouabain-Binding Site-EO-BP Connection*).

There should now be no doubt about the existence of eCTS, and that ouabain, per se, is endogenous in humans and other mammals; it is widely referenced as such. For example, “Ouabain, an endogenously synthesized hormone… (is) capable of inducing mitogen-activated protein (MAP) kinase phosphorylation and intracellular Ca^2+^ signaling…” (267) and “Endogenous cardiotonic steroids… including ouabain… are now accepted as a class of steroid hormones involved in BP regulation and sodium handling” (268).

### EO Is Not the Only Endogenous CTS in Humans and Other Mammals

We have already alluded to the detection of two EO isomers in rat plasma (115, 118); these isomers have not been isolated in sufficient quantity to determine their structures or test for their ability to inhibit NKA. Although an ouabain isomer was initially reported present in bovine hypothalamus based upon the circular dichroism of naphthoylated derivatives (269) that substance was later claimed to be an unrelated chemical contaminant (glycerol). Subsequent studies from the same laboratory determined that the main brain compound was indeed EO (ouabain), itself (254). Importantly, while EO has been studied most extensively, numerous reports indicate that mammals, including humans, also have circulating endogenous digoxin-like compounds and bufadienolides (211, 215, 231, 235, 270). Some of these are undoubtedly present in the brain in varying amounts.

During the initial isolation and characterization of EO in human plasma, four major and at least two minor fractions that were separated by HPLC inhibited ouabain-sensitive ^86^Rb uptake by human erythrocytes (Supplemental Fig. S1) (229), the “gold standard” test for CTS-like inhibition of the Na^+^, K^+^-pump. The major biologically active fraction also cross-reacted strongly with ouabain-selective, but not digoxin-selective, antibodies and was identified as ouabain by MS (209). Another biologically active fraction cross-reacted strongly with digoxin-selective, but not ouabain-selective antibodies; the third and fourth major and two minor biologically active fractions unfortunately were not further characterized (229). In a study using only immunological methods, peritoneal dialysis fluid from patients in chronic renal failure was separated into three distinct “digitalis-like immunoreactive factor” fractions by HPLC. One fraction coeluted with ouabain and a second “co-eluted precisely with digoxin”; the third peak was not identified. Functional activity of the peaks (e.g., NKA inhibitory activity) was not tested and they were not subjected to MS (271). Goto and co-workers (90) isolated a substance from large volumes of human urine that inhibited ^3^H-ouabain binding to human erythrocytes and was indistinguishable from digoxin by MS and NMR. Valdes and colleagues isolated a substance from bovine adrenal cortex with digoxin-like immunoreactivity that had “some structural similarity,” but not identity, to digoxin (272). They also demonstrated that a mouse adrenocortical tumor cell line could synthesize steroids that cross-reacted with digoxin-selective and dihydrodigoxin-selective antibodies (273). Together, these observations indicate that there are one or more digoxin-like eCTS in mammalian plasma.

Bufadienolides are synthesized in plants and amphibians (274), and marinobufagenin (MBG) (Fig. 1*B*) has been analytically identified in human plasma (110, 275, 276); its biosynthesis has been suggested to occur in the adrenals via a bile acid pathway (277). Komiyama and collaborators (110) identified both MBG and a novel bufadienolide, telocinobufagin, in human plasma by MS and NMR; they found elevated plasma levels of telocinobufagin in patients in terminal renal failure. Other bufadienolides have also been isolated and identified by analytical methods in human plasma and tissues: proscillaridin A in plasma (231), 3β-OH, 14α20:21 bufenolide in the placenta (278), and 19-norbufalin in cataractous lenses (97). The 19-norbufalin inhibits rat brain microsome NKA and ^3^H-ouabain binding to rat synaptosomal membranes; it cross-reacts poorly with anti-digoxin-specific antibodies (97). It has been reported, however, that eCTS that cross-react with anti-bufalin antibodies were not detected in bovine or rat adrenal glands (279).

With these several potential eCTS, albeit not yet all identified analytically, circulating around the mammalian body it is surely prudent to ask: What are they all doing? In the following sections, we attempt to summarize much of what is currently known, although it will become abundantly clear that there is still a lot to learn.

## CTS BINDING BY THE NKA IS A COMPLICATED AFFAIR

### Surprising CTS-NKA Interactions Defy Dogma

#### Stimulation of NKA by low CTS concentrations.

Based on Schatzmann’s pivotal 1953 publication (56) and numerous subsequent studies showing that cardenolide and bufadienolide CTS all inhibit the NKA (58), it was widely believed that these CTS are simply “full antagonist” blockers of the NKA. Seven years after Schatzmann’s article appeared, his institute director and former mentor, Wilbrandt, reported that low doses of CTS increased frog skin transepithelial potential (78), a measure of active Na^+^ transport (280); higher concentrations reduced the potential to near zero (Table 2). The apparent stimulation was a surprise because CTS had been expected only to inhibit the NKA (57) and thereby reduce the transepithelial potential (280, 301, 302). Several other studies indicated that low-to-therapeutic concentrations of exogenous CTS have little or no effect on cell Na^+^ and K^+^ or even augment their ion gradients, whereas higher “toxic” concentrations invariably raise cell Na^+^ and reduce K^+^ (303–306). This implies that the dose-response relationship between these CTS and tissue electrolytes could be biphasic wherein the stimulatory concentrations might be 10-to-500-fold lower than the EC_50_ for the inhibitory and toxic effects. Indeed, this has been one of the long-standing enigmas regarding the CTS-NKA interaction: the inability of picomolar to low nanomolar concentrations of ouabain and/or digoxin to raise tissue Na^+^ and K^+^ concentrations.

**Table 2.** Studies that reported a stimulatory effect of low concentrations of cardiotonic steroids* on NKA

Year	Species	Preparation	Stimulating Agent	Likely α Isoform Target	Max Stimulatory Effect§	Transient and/or Aging Effect§§	References
1960	Frog	Skin potential	K-strophanthoside	α1	90	Yes	(78)
1963	Guinea pig	Cardiac myocyte membrane Na,K-ATPase	Ouabain Digitoxigenin Dihydrodigitoxin K-Strophanthoside Digitoxin	α1, α2	30 55 45 43 16	Yes	(79)
1963	Bovine	Ventricular sarcolemmal Na,K-ATPase	Digitoxigenin	α1, α2	∼5		(281)
1963	Guinea pig	Cardiac microsomal Na,K-ATPase	Strophanthin-K	α1, α2	∼25	Yes	(282)
1964	Rabbit	Brain microsomal Na,K-ATPase	Ouabain	α1, α2, α3	8.6		(283)
1964	Chicken	Kidney microsomal Na,K-ATPase	Ouabain	α1	33		(284)
1964	Chicken	Renal Na^+^ reabsorption in vivo	Ouabain	α1	>40		(284)
1965	Toad	Bladder short circuit current	Ouabain	α1	∼20	Yes	(285)
1966	Cat	Cerebrospinal fluid production Choroid plexus Na,K-ATPase	Ouabain Ouabain	α1, α2, α3	45 10–29		(286)
1966	Chicken Rabbit	Kidney microsomal Na,K-ATPase Brain microsomal Na,K-ATPase	Ouabain Ouabain	α1 α1, α2, α3	20–38	Yes	(287)
1972	Squid	Giant axon ^22^Na^+^ efflux	Ouabain	α1, α3	20		(288)
1976	Sheep	Outward current and twitch tension in Purkinje fibers	Ouabain	α1, α2	∼20†		(289)
1978	Sheep	Intracellular Na^+^ in cardiac Purkinje fibers	Strophanthidin Acetylstrophanthidin	α1, α2	∼15†	Yes	(290)
1979	Guinea Pig	Intracellular Na^+^ in stimulated cardiac atria	Ouabain	α1, α2	∼20		(291)
1983	Sheep	Outward current and twitch tension in Purkinje fibers	Ouabain Strophanthidin Digoxin	α1, α2	∼10† ∼25	Yes	(292)
1985	Human Dog	Red cell ghost Na,K-ATPase Kidney microsomal detergent treated Na,K-ATPase	Ouabain Ouabain	α1 α1	∼25∼ 20††	Yes	(88)
1987	Rat	Brain homogenate Na,K-ATPase	Ouabain	α2, α3	5–70††	Yes	(293)
1998	Human	Rb^+^ uptake in mesenteric artery rings	Ouabain, Marinobufagenin	α1, α2	33–58		(294)
2002	Guinea Pig, Dog, Human	Ventricular myocyte Na^+^ pump current	Dihydroouabain	α1, α2	25–107†††		(295)
2004	Human	Umbilical artery endothelial cells	Ouabain	α1, α2	15–50		(296)
2006	Opossum	Rb^+^ uptake in renal proximal tubule cells	Ouabain	α1	∼40†		(297)
2007	Human	Red cell Rb^+^ uptake	Ouabain, Ouabain-like factor, Digoxin	α1	14–18 0		(298)
2010	Rat	Hippocampal slices ^86^Rb uptake	Ouabain, Digoxin, Marinobufagenin	α2, α3	40–80		(299)
2016	Human	Umbilical vein [K^+^]_i_/[Na^+^]_i_ Membrane NKA	Ouabain	α1, α2	20–50 25–30		(300)
2018	Insect Sf9 cells	Membrane NKA	γ-Benzylidine digoxin derivatives	α1-3	25–300††††		(179)

*Typical stimulatory concentrations of the indicated agent ranged between 10 pM and 10 nM. Higher concentrations invariably led to inhibition of the measured parameter.

**§**Percent increase above control (Na,K-ATPase activity, ouabain-sensitive ^86^Rb uptake, ouabain-sensitive ^22^Na^+^ efflux, ouabain-sensitive short circuit current, ouabain-sensitive renal sodium reabsorption, ouabain-sensitive CSF production, outward sodium pump currents) or % decline in intracellular sodium concentrations.

§§Transient effect: stimulation followed, usually over minutes to tens of minutes, by either a return to baseline or the onset of inhibition depending on the dose of CTS used. Aging effect includes the progressive loss of stimulation with time typically observed in ATPase reactions, as well as the progressive loss of stimulation in tissues during prolonged super/perfusion, or with the prolonged storage of membrane preparations.

†Not stated; inferred from recordings.

††Magnitude of ouabain activation inversely proportional to baseline specific activity.

†††Upper value calculated from a model and ascribed specifically to stimulation of the α-2 subunit isoform.

††††NKA α subunit isoforms 1–3 were coexpressed with β1 in insect Sf9 cells. Certain synthetic γ-benzylidene-digoxin derivatives showed isoform-selective effects. Some analogs (BD-3, BD-8, and BD-13) stimulated all NKA isoforms while others selectively inhibited α1 and α3 but not α2 (see text for details). The magnitude of the stimulatory effects as well as the concentrations evoking maximal stimulation and inhibition were notably higher than observed with digoxin and other naturally occurring CTS.

**Figure 2.**
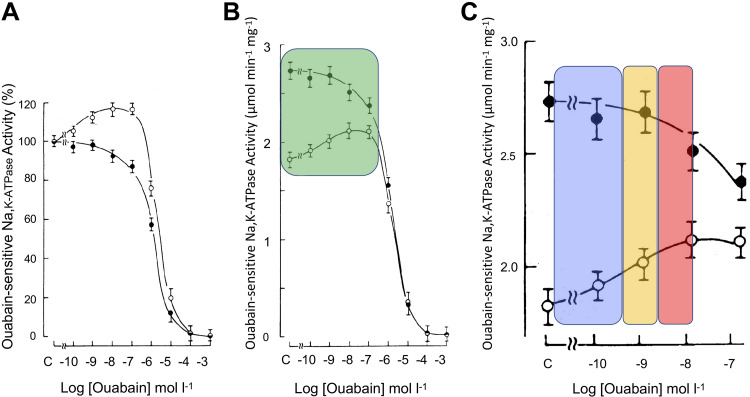
Stimulation and inhibition of a dog kidney NKA membrane preparation by ouabain. *A*: graph shows the dose-response relationships for the NKA preparation after rapid washing in high Na^+^ or K^+^ solutions. The Na^+^-washed NKA (O) retained its ability to be stimulated by low concentrations of ouabain, whereas the K^+^-washed enzyme (•) did not. Each data point is the mean of 4 determinations ± SD and is presented as a % of control activity. *B*: data from the same experiments but presented in terms of absolute-specific NKA activity. The specific activity of the potassium-washed NKA increased but lost all ability to be stimulated by ouabain. *C*: the green area highlighted in *B* is enlarged in *C* to illustrate the physiological (blue) range for ouabain- and digoxin-like eCTS, as well as the therapeutic (orange) and toxic/lethal (red) values for ouabain and digoxin in the human circulation. The upper value for these eCTS is at least one order of magnitude lower than the concentration of their exogenous counterparts given therapeutically. CTS, cardiotonic steroid; NKA, Na^+^, K^+^-ATPase (Na^+^ pump). Illustrations reproduced and modified from Ref. 88) with permission of The Company of Biologists Ltd.

In 1963, Repke described these observations on tissue electrolytes as an inverted U-shaped concentration-activity curve after reproducing this relationship in a membrane NKA preparation (79). The stimulation phenomenon has been confirmed in diverse biochemical and physiological preparations (Fig. 2 and Table 2). This phenomenon is notable for its specific concentration dependence, magnitude, temporal characteristics, CTS specificity, and occurrence throughout the animal kingdom. It also appears to be a fundamental property of three and possibly all four of the mammalian NKA α subunit isoforms.

Despite the numerous reports (Table 2), the significance and mechanism of the stimulation phenomenon remained largely ignored. With the benefit of hindsight, we now realize that the concentration dependence of the stimulation phenomenon overlaps directly with the levels of EO and other eCTS reported in the circulation of humans and other species (135, 307) (and see Supplemental Fig. S9*B*). This, in turn, raises a number of intriguing issues and complications in understanding the physiological consequences of variations in circulating eCTS levels and the nature of their interactions at the NKA ouabain-binding site in vivo. As illustrated in Fig. 2, for example, increases in ouabain (or circulating EO) starting from the low picomolar up to the low nanomolar range can stimulate NKA activity. This is paradoxical because increases in the ambient concentration of ouabain (and EO) in exactly the same concentration range would be expected to inhibit the NKA and reduce its activity. Furthermore, it has been widely noted that therapeutic levels of ouabain or digoxin in the very low nanomolar range (1–3 nM), i.e., those typically observed in the circulation during the treatment of cardiac conditions (84, 307), initially stimulate NKA, and then, as the incubation proceeds, may become inhibitory (88). It is not known if the stimulation observed with subtherapeutic concentrations of ouabain or digoxin (i.e., those within the physiological range) can persist indefinitely. At much higher, toxic concentrations (30–1,000 nM), the initial stimulatory phase is often brief or not detected so that only profound inhibition of NKA is observed.

The magnitude of the reported stimulatory effect evoked by ouabain varies from very modest to large (∼5–70%; Table 2) with the latter being observed in rat brain homogenates (293). Most of the larger stimulatory effects were observed in preparations from CTS-sensitive animals including chicken, dog, and cat. They were also observed, however, in some preparations from animals with ouabain-insensitive α1 NKA such as the rat where the phenomenon is most likely mediated by the highly ouabain-sensitive α2, α3, and/or α4 subunit isoforms of the NKA (e.g., in brain and testis).

Efforts to improve the α isoform selectivity of the CTS have led to a series of synthetic γ-benzylidene-digoxin (BD) derivatives with distinct lactone ring substitutions; several of these agents produced unanticipated stimulatory effects on NKA activity (179). Insect Sf9 cells separately transfected with the α subunit isoforms 1–3 in the presence of β1 were used to explore the α selectivity of a series of synthetic analogs. Some of the analogs (BD-3, BD-8, and BD-13) between 10 and 1,000 nM stimulated α2 approximately threefold, while others (BD-5) at 10 µM selectively inhibited α1 and α3 but not α2. Similarly, BD-10 inhibited α1 at all concentrations >10 nM but stimulated α2 and α3 ∼1.5- to 2-fold. Yet others (BD-14, BD-15, and BD-16) preferentially stimulated α3 ∼2–2.5-fold.

Critically, the mechanism of the stimulatory phenomenon, both with naturally occurring and synthetic CTS, especially in isolated membrane preparations, remained unclear. Initially, it was suggested that detergent-like actions of ouabain and digoxin might expose latent substrate sites but this is highly unlikely because nanomolar concentrations of these CTS have negligible detergent-like action. A stimulatory effect of catecholamines, whose release can be augmented by ouabain and other CTS, was also excluded (281).

The first clue to the stimulatory mechanism came in 1985 when tests of a partially purified detergent-treated dog kidney NKA raised the possibility of displacement of an inhibitory factor (88). This NKA preparation was routinely stimulated by subnanomolar concentrations of ouabain; stimulation persisted after the preparation was quickly washed in high Na^+^ medium but was absent after washing in high K^+^ medium (Fig. 2). While the original preparation and that washed with Na^+^ had comparable specific activities, the activity of NKA increased after the K^+^ wash and the stimulatory effect of ouabain was absent. Hence, the loss of stimulation and increase in specific activity occurred simultaneously and were related. This led to the proposal that the stimulation phenomenon reflects the loss of an endogenous inhibitor and that it is not a “true” stimulation per se (88). Notably, the NKA in the washed membranes in both cases was fully inhibited by high concentrations of ouabain.

The second clue was based on the expectation that the ability of ouabain to stimulate the NKA should be related to the initial specific activity of the NKA if one assumes the following sequence of events: *1*) the binding of an endogenous inhibitor in vivo dose-dependently reduces a portion of NKA activity. *2*) This binding survives all steps leading to isolation of PM containing NKA. *3*) When ouabain is applied in low doses in vitro, the magnitude of the evoked stimulation would be expected to be inversely correlated to the fraction of NKA inhibited in vivo. This expectation was realized by the direct observations of Lichtstein (293). From a mechanistic perspective, the idea that an endogenous inhibitory factor (eCTS or otherwise) can be displaced from the NKA by the binding of low concentrations of another inhibitor is most unusual, especially if the endogenously bound and exogenous inhibitors are both CTS entities and there is only one binding site per α subunit.

The third crucial clue followed from the analysis of the antagonism between ouabain and digoxin in isolated small arteries and cultured neurons (61). To explain this peculiar interaction, a tetraprotomeric form of NKA was invoked de novo. Accordingly, modeling studies indicated that the stimulation phenomenon resulted from the displacement of an endogenous inhibitor from a tetrameric form of the NKA (see next section) and that a high K^+^ wash could also remove the endogenously bound inhibitor as outlined in Supplemental Section S1.

Many investigations listed in Table 2 indicated that the magnitude of the stimulation often varied dramatically from preparation to preparation. This might be explained by several factors. First, the secretion of the endogenous inhibitor may be episodic so that the in vivo concentrations of free and bound inhibitor may have differed before euthanasia. Second, the inhibitor concentrations may reflect the method of euthansia; some eCTS are increased by hypoxia (308–313). Third, the typical NKA isolation, storage (most would be suspended in liquid), and use conditions may have allowed the inhibitor to dissociate. For example, virtually all of the physiological preparations listed in Table 2 typically were perfused/superfused for extended periods before recordings were made, and some or all of any prebound inhibitor will have dissociated and washed away. Fourth, in the ATP hydrolysis studies, the greatest observed NKA stimulation (∼70%) occurred in crude homogenates where the time and number of manipulations are expected to be minimal (293). Fifth, the stimulation phenomenon has never been reported in NKA preparations highly enriched by SDS or C12E8 detergents in the presence of millimolar ATP (314, 315). This seems logical; the usual enzyme handling conditions often not only favor the rapid unbinding of ouabain, digoxin, and their endogenous counterparts from NKA but also minimize the tetrameric form of NKA upon which stimulation depends (63, 296, 315–319). The method used to prepare the NKA preparation studied in Fig. 2, *A* and *B* was gentle; it was modified from a published method (320) and used a low concentration of deoxycholate in the absence of added ATP and presence of EDTA to chelate Mg. It also involved a subsequent lyophilization. Fortuitously, this procedure allowed the endogenous inhibitor to remain attached throughout isolation and during prolonged storage. It is noteworthy that ouabain can remain tightly bound to NKA under similar mild enrichment conditions (321, 322); see Supplemental Sections 1 and 2.

With regard to the synthetic γ-benzylidene-digoxin derivatives mentioned earlier, both the stimulatory as well as the inhibitory effects of these analogs are shifted to considerably higher concentrations than observed with digoxin and many other naturally occurring CTS (179). The magnitude of some of the stimulatory effects are especially noteworthy, reaching almost 300%. This implies that these analogs evoke near complete dissociation of α-subunit tetramers to protomers without generating much residual inhibition. One of these analogs (BD-15) stimulated NKA α3 activity in the brain but not in heart indicating isoform preference for the brain in vivo where the NKA isoforms are in their usual membrane environments (323).

#### All CTS are not created equal: ouabain-digoxin antagonism and other strange effects.

**Figure 3.**
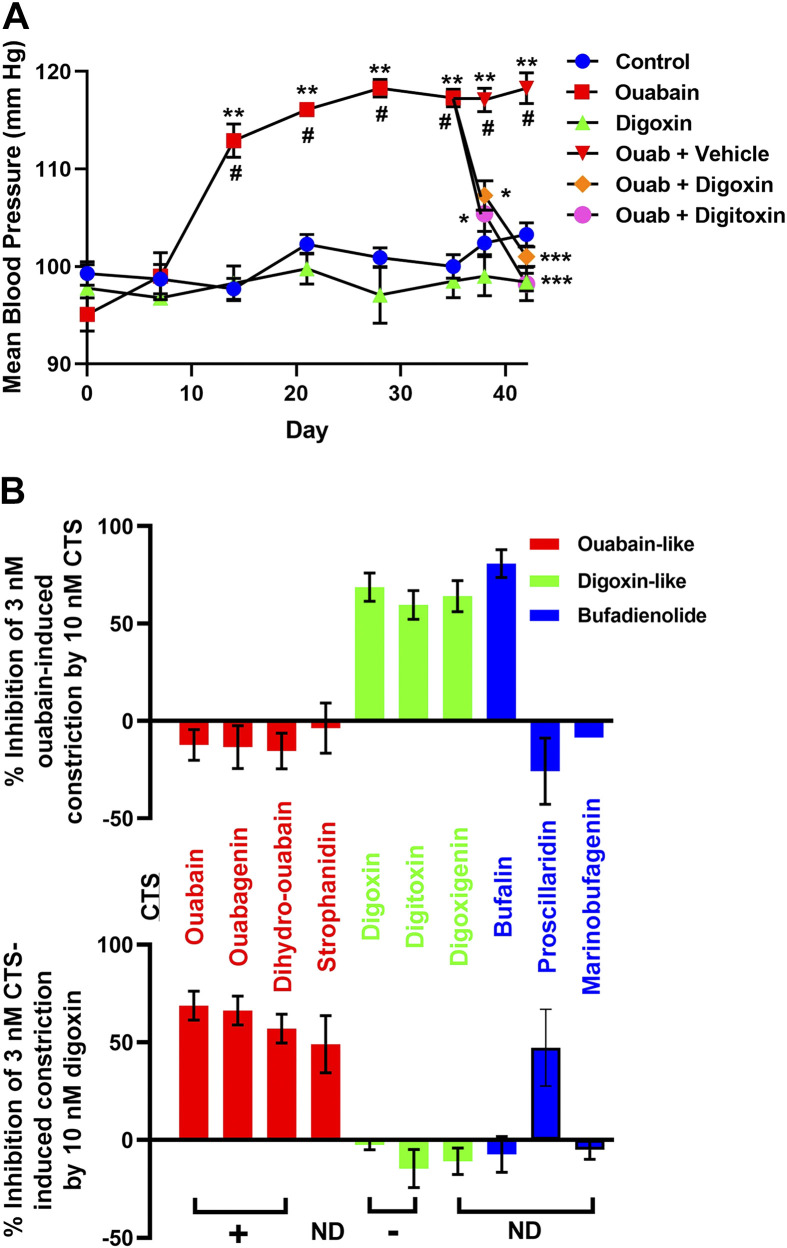
Ouabain-induced hypertension and ouabain-digoxin antagonism. *A*: prolonged infusion of ouabain, but not digoxin, elevates blood pressure (BP) in rats; digoxin antagonizes the effect of ouabain. Sprague-Dawley rats were infused by subcutaneous osmotic minipumps with vehicle, ouabain (15 µg/kg/day) or digoxin (30 µg/kg/day) for 6 wk. After 5 wk, some ouabain-infused rats (*n* = 8/group) received a secondary infusion of vehicle, digoxin (30 µg/kg/day) or digitoxin (30 µg/kg/day). Mean BPs were measured by tail cuff. *P < 0.05 vs. ouabain; ***P < 0.001 vs. ouabain; #*P* < 0.005 vs. vehicle; **P < 0.001 vs. digoxin. In a second study, after infusion of vehicle, ouabain, or digoxin for 5 wk, plasma steroid immunoreactivity was measured with ouabain- or digoxin-specific immunoassays. The levels were: vehicle + endogenous ouabain (EO), 0.66 ± 0.12 nM; ouabain-infused, 5.2 ± 0.39 nM ouabain + EO; digoxin-infused, 3.3 ± 0.32 nM digoxin (*n* = 8 rats/group). Data are regraphed from Ref. 96 and presented with permission. *B*: the abilities of various cardiotonic steroids (CTS) to alter myogenic tone in isolated rat mesenteric small arteries pressurized to 70 mmHg (MT_70_). Summarized data show the relative abilities of various CTS to antagonize the 3 nM ouabain-induced increase in MT_70_ (upper row of bars), and the ability of 10 nM digoxin to antagonize the increase in MT_70_ induced by various CTS (lower row of bars). The data (means of 5–8 arteries ± SE per bar) are expressed as: b/a × 100% where “a” is the 3 nM ouabain-induced (top) or other CTS-induced (bottom) constriction and “b” is the constriction when 10 nM other CTS (top graph) or digoxin (bottom graph) was added. Positive values indicate vasodilation (or antagonism); negative values indicate vasoconstriction (or synergism) induced by the second CTS. The ability of the various CTS to induce hypertension when infused subcutaneously (96, 326) is indicated at the bottom: “+” agents induce hypertension; “–“ agents do not induce hypertension and are antihypertensinogenic (i.e., they antagonize the hypertensinogenic effect of ouabain); ND, not determined. The figure is regraphed from Ref. 61 and reproduced with permission.

Following the discovery of EO, it seemed logical to test for the possibility that ouabain is an hypertensinogenic agent, as indirectly suggested by the correlation between a circulating NKA inhibitor and BP (85). Indeed, in 1993, Pamnani and colleagues (94) showed that prolonged administration of ouabain to rats elevated BP. But the results posed a dilemma; ouabain and digoxin both inhibit the NKA in similar fashion, yet digoxin was never reported to induce hypertension even though it was used widely to treat HF for more than two centuries. In fact, digoxin has no effect on daytime BP in healthy subjects or patients with HF, and it modestly decreases nighttime diastolic and mean BPs (324); digoxin has even been reported to lower BP in patients with hypertension (325). Therefore, one of us (JMH) tested the effect of prolonged administration of ouabain and digoxin in rats. Not only did digoxin not elevate BP, but it antagonized the hypertensinogenic effect of sustained ouabain administration, as did a related *Digitalis* glycoside, digitoxin (Fig. 3*A*) (95, 96, 327). These remarkable results (functional selectivity or “biased signaling”) revealed that ouabain and digoxin do not have identical effects and that these two cardenolides can be antagonistic to one another under some circumstances. Nevertheless, this finding, too, was largely ignored, it did not fit the prevailing dogma. The manuscript was initially rejected for publication, and the full article (96) only appeared several years after the preliminary reports (95, 327). Even before the full article was published, others had replicated the finding and shown that digoxin also greatly attenuates salt-dependent hypertension in rats (328).

Ouabain-digoxin antagonism was subsequently also demonstrated in guinea pig heart (329) and rat-resistance arteries and neurons (Supplemental Figs. S4 and S5) (61). Bufalin, too, antagonizes the acute cardiotonic and vasotonic effects of ouabain, but not digoxin, in the heart and arteries (61, 329). Also, bufalin and digoxin enhance membrane endocytosis in NT2 (human neuronal precursor) cells; ouabain does not, but it does block the membrane trafficking effects of bufalin and digoxin (330). MBG, however, does not antagonize the vasoconstrictor action of either ouabain or digoxin on isolated arteries (Fig. 3*B*) (61). In contrast, the bufadienolide, proscillaridin A, is “ouabain-like” in that it antagonizes the effect of digoxin, but not ouabain, on isolated rat arteries (61). In the arteries, several *Digitalis* steroids antagonized the action of ouabain, but not digoxin, whereas several *Strophanthus* steroids antagonized the action of digoxin, but not ouabain (Fig. 3*B*). Thus, in more general terms, we may speak of *Strophanthus*-like and *Digitalis*-like actions. Interestingly, bufadienolides apparently may be either *Strophanthus*-like (proscillaridin A) or *Digitalis*-like (bufalin), or neither (MBG) (61).

The concentration ratio between ouabain and digoxin or digoxin-like eCTS must be a determinant of their respective net impact on BP because the chronic pressor effect of ouabain in rodents is reversed by digoxin. This raises a question about the importance of dietary factors related to digitalis in trying to understand the chronic pressor effects of ouabain. Although there are no recognized common dietary sources of ouabain and, in any event, its intestinal absorption is minimal, digoxin and digitalis-like CTS are long recognized to be present intermittently in the diets of some livestock, are bioavailable, and can be ingested to excess (182–186, 331). These instances of toxicity apparently are rare, but the point is that unrecognized exogenous sources of digitalis steroids in the diet as well as those produced endogenously might offset the hypertensinogenic activity of ouabain.

#### Another dogma-defying observation: the NKA mediates CTS-activated signaling.

Shortly after ouabain-digoxin antagonism was first demonstrated, Askari, Xie and colleagues made the seminal discovery that ouabain-NKA interaction induces the Ca^2+^-dependent expression of some early-response genes (6) and activates several PK signaling pathways (7, 102). For example, cardiomyocyte Src kinase, protein kinase C (PKC), and extracellular signal-related kinases (ERK)1/2 activation accompanied the cardiotonic response to 1–50 µM ouabain in intact guinea pig and rat hearts (332), but whether the PK effect was directly related to the cardiotonic response was not examined. Rosen and Lichtstein, however, explored the effects of CTS on membrane trafficking (330) and showed that NKA inhibition, either by ouabain or reduction of extracellular K^+^, did not promote trafficking whereas digoxin, digoxigenin, bufalin, and proscillaridin A all did. Clearly, the trafficking effect was independent of NKA inhibition.

Subsequently, Golovina and colleagues studied arterial myocytes isolated from rats after subcutaneous (SC) infusion of ouabain and digoxin (both 30 µg/kg/day x 3 wk), or vehicle (117). The ouabain-, but not digoxin-, infused rats developed hypertension, and arterial myocytes isolated from the ouabain-hypertensive rats exhibited elevated basal [Ca^2+^]_CYT_ and augmented phenylephrine (PE)-induced Ca^2+^ transients despite the absence of CTS in the incubation media (Supplemental Fig. S6, *A–C*). Phosphorylation of Src kinase Tyr-418 (Y418) was increased and phosphorylation of ERK1/2 was decreased only in the arterial myocytes of ouabain-treated rats (Supplemental Fig. S6*D*), while total Src and ERK1/2 were unchanged. Two transporter proteins involved in Ca^2+^ homeostasis and signaling, NCX1, and transient receptor potential channel-6 [TRPC6, a cation channel with low selectivity for Ca^2+^ over Na^+^ (333)] were upregulated in the arterial myocytes from the ouabain-treated rats (Supplemental Fig. S4*E*).

Similarly, primary cultured rat arterial myocytes treated for 72 h with 100 nM ouabain, but not with digoxin, exhibited elevated basal [Ca^2+^]_CYT_, augmented PE-induced Ca^2+^ transients, and upregulated NCX1 and TRPC6 proteins (Fig. 6, *A*–*C*). Furthermore, the ouabain-induced transporter protein upregulation was completely suppressed if digoxin was included with ouabain in the incubation medium (i.e., ouabain-digoxin antagonism), or if Src kinase inhibitor, PP2, but not the inactive analog, PP3, was included with ouabain (Fig. 6, *C*–*E*) (117). This implies that the transporter protein upregulation was mediated by ouabain-triggered Src activation. In contrast, the acute application of digoxin as well as ouabain rapidly augments Ca^2+^ signaling and contraction in cardiac (336) and arterial myocytes (61), and Ca^2+^ signaling in neurons (61) (Supplemental Figs. S4 and S5).

In sum, these findings not only demonstrate ouabain-digoxin antagonism but also show that the *Digitalis* steroids and *Strophanthus* steroids have some selective (biased) effects even though they all inhibit NKA and are acutely cardiotonic and vasotonic. The data imply, further, that the elevated BP in ouabain-hypertensive rats is not due solely, or even primarily, to acute inhibition of NKA and the consequent vasotonic effect. Rather, it is most likely due largely to ouabain-triggered modulation of PK signaling and to the ouabain/EO-regulated central nervous system control of sympathetic drive (see *The NKA Ouabain-Binding Site-EO-BP Connection*).

**Figure 4.**
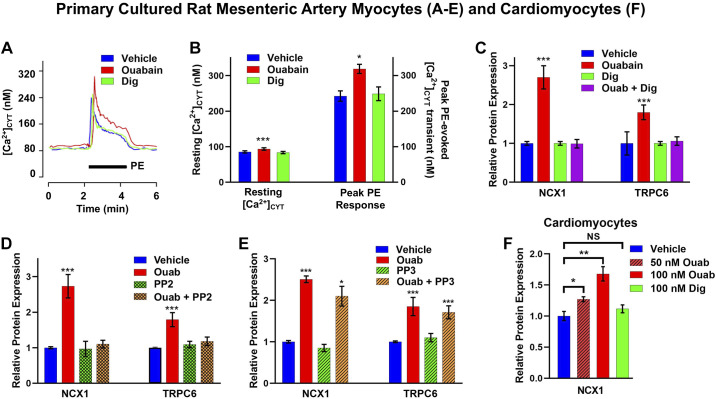
Prolonged incubation with ouabain, but not digoxin, augments Ca^2+^ transporter expression in primary cultured rat arterial and cardiac myocytes; Src kinase inhibition prevents the upregulation in the arterial myocytes (cardiomyocytes were not tested). Primary cultured rat superior mesenteric artery myocytes and left ventricular cardiomyocytes were incubated for 72 h with vehicle, ouabain (Ouab, 50 or 100 nM), digoxin (Dig, 100 nM), or ouabain + digoxin (Ouab + Dig, 100 nM, each). In some instances, the arterial myocytes were incubated with the Src kinase inhibitor, PP2 (4-amino-5-(4-chlorophenyl)-7-(*t*-butyl)pyrazolo[3,4-*d*]pyrimidine, 1 µM), or its inactive analog, PP3 (4-amino-7-phenylpyrazol[3,4-d]pyrimidine, 1 µM), both without and with 100 nM ouabain. *A* and *B*: after washout of the cardiotonic steroids, the cytosolic Ca^2+^ concentration ([Ca^2+^]_CYT_) in the arterial myocytes was measured with Fura-2, both at rest and during stimulation, with 1 µM phenylephrine (PE). *A*: representative cell recordings. *B*: resting: *n* = 298–316 cells/bar ± SE; peak: *n* = 39–49 cells/bar ± SE. *C–E*: the effects of ouabain (100 nM), digoxin (100 nM) and ouabain + digoxin, and of PP2 (Src kinase inhibitor) and PP3 (inactive PP2 analog), both without and with ouabain, were tested on arterial myocyte Na^+^/Ca^2+^ exchanger-1 (NCX1) and transient receptor potential C6 (TRPC6) protein expression. Each bar shows the mean of 4–8 immunoblots ± SE. *F*: Ouabain (50 and 100 nM), but not digoxin (100 nM), also upregulates NCX1 expression in cardiomyocytes. *P < 0.05; **P < 0.01; ***P < 0.001 vs. vehicle and digoxin. Data from Ref. 117 (*A–E*) and Ref. 341 (*F*) regraphed and reproduced with permission.

Acute application of either ouabain or digoxin to cells (e.g., myocytes and neurons) inhibits NKA and, via NCX, elevates basal [Ca^2+^]_CYT_, increases agonist-evoked Ca^2+^ signals and, in cardiac muscle and VSM, augments contractions (59, 61, 336–340). Recovery is rapid after CTS washout (Supplemental Figs. S4 and S5) because the enhanced contraction depends upon the acutely altered transporter function and not on slow effects such as PK signaling and modification of protein expression. Following prolonged exposure to ouabain, agonist-evoked Ca^2+^ transients in primary cultured or freshly isolated arterial myocytes are increased (117), as in acutely treated cells and arteries. The washout of ouabain during isolation and incubation of myocytes from ouabain-hypertensive rats does not, however, rapidly reverse the amplification of the agonist-evoked responses. This amplification is due to the aforementioned sustained Ca^2+^ transporter protein upregulation (Fig. 4 and Supplemental Fig. S6*E*). In striking contrast, the agonist-evoked Ca^2+^ transients in arterial myocytes exposed to digoxin for a prolonged period (3 days in vitro or 3 wk in vivo) do not differ from the Ca^2+^ transients in control cells (Fig. 4, *A* and *B,* and Supplemental Fig. S6, *B* and *C*) (117). In other words, when treatment is prolonged, digoxin apparently does not maintain its vasotonic effect and does not alter arterial myocyte PK signaling and protein expression, whereas ouabain does. Obviously, the mechanisms underlying the sustained vasotonic effect of ouabain differ from those responsible for the acute effect.

In primary cultured rat cardiac myocytes, too, 72-h incubation with low-dose ouabain upregulates NCX1 expression, whereas digoxin does not (Fig. 4*F*) (341). This appears to belie the widely held view that the long-term beneficial effect of *Digitalis* preparations in HF is due to their cardiotonic action (173) – “The fundamental therapeutic… action of digitalis is clearly the stimulation of ventricular contractile state” (342). Some authors have, in fact, suggested that digoxin’s beneficial effect may be due, not to a cardiotonic action but, rather, to its neuromodulatory action in suppressing the renin-angiotensin-aldosterone system (RAAS) and sympathetic drive (343). Alternatively, digoxin’s antagonism of ouabain action may be a better, more mechanistic explanation for digoxin’s therapeutic efficacy in HF (see *Are NKA Ouabain-Binding Sites and EO Involved in the Manifestations of Heart Failure?*). Justification for this view is the observation that bufalin is digitalis-like in its actions (Fig. 3*B*) (61) and exhibits bufalin-ouabain antagonism in the heart (329). Most investigators consider “ouabain” and “digitalis” interchangeable (e.g., Refs. 344 and 345) and seem unaware that ouabain (i.e., *Strophanthus* glycosides) and *Digitalis* glycosides can have different effects. Ouabain is used for most research simply because it is highly water soluble and, thus, more convenient to use than digoxin, which typically requires an organic solvent. Many of the aforementioned findings underscore the biased actions of different CTS species on PK signaling, which was recently reaffirmed by a report on different bufadienolides (346) and one on different cardenolides (347).

#### Anomalies of ouabain kinetics.

The actions of these low nanomolar concentrations of ouabain led to another unanticipated observation. Earlier studies of ouabain-binding kinetics, usually measured as equilibrium binding to red blood cells (RBCs) or isolated membrane preparations, had demonstrated that the rates of binding and dissociation are quite slow. This is exemplified by the time-course of [^3^H]-ouabain binding to, and dissociation from, human RBCs and RBC membranes; the ouabain saturates the binding sites with a half-time of >30 min, and only ∼50% is washed out in a similar period (Supplemental Fig. S7) (57, 348).

This kinetic behavior contrasts markedly with observations in vascular, neuronal, and cardiac preparations of which Supplemental Fig. S5 is an example (61). In those experiments, incubation of the primary cultured rat neurons with 3 nM ouabain or 10 nM digoxin for only 5 min was sufficient to augment, robustly, glutamate-evoked Ca^2+^ transients. Furthermore, the CTS washout was virtually complete within 5 min (the earliest time tested). An even clearer illustration of the rapid on- and off-time course of ouabain’s physiological effects is seen in Supplemental Fig. S4 (61). In these isolated rat mesenteric small arteries, 3 nM ouabain or digoxin induced peak vasoconstriction within 3–5 min and washout was similarly fast (Supplemental Fig. S4, *A* and *B*). Following destruction of the endothelium, 0.1 nM ouabain swiftly constricted the artery and swiftly washed off (Supplemental Fig. S4C). The antagonizing effects of low concentration digoxin or ouabain on, respectively, ouabain- or digoxin-constricted endothelium-intact or -denuded arteries were similarly rapid (Supplemental Figs. S4 and S5) (61). Importantly, a number of other reports have documented comparably rapid physiological responses and washout with low nanomolar ouabain (e.g., Refs. 349–352) as well as with higher ouabain and MBG concentrations (e.g., Refs. 59, 353, 354).

It is astonishing that these numerous, highly unusual CTS actions/interactions (355) which have been known for decades, including anomalous ouabain kinetics, ouabain-digoxin antagonism, CTS-activated NKA-mediated signaling, and the biased actions of different CTS, are not yet mentioned in standard textbooks (173). These data are largely ignored; they do not fit the prevailing dogma, the classic picture of the NKA as an αβ protomer that operates simply as a CTS-inhibitable cation pump. Nevertheless, it should be obvious that conventional understanding of the interaction of ouabain and digoxin with the NKA, and their physiological consequences, is deeply flawed. We therefore now turn to structural studies of the NKA and its ouabain-binding site for possible insight into these issues.

### What is the Molecular Structural Basis for Distinct Actions of Strophanthus-Like and Digitalis-Like CTS?

The NKA α subunit contains a single CTS binding site. Even before the NKA was cloned and its molecular structure determined, Richard Thomas had deduced the basic architecture of the ouabain-binding site from CTS structure-activity relationship studies (84). He inferred that the CTS inserts lactone ring first into a cleft or pocket in the NKA that is accessible to the extracellular fluid. The CTS forms hydrogen bonds as well as multiple Van der Waals and/or hydrophobic interactions with the receptor. Thomas also recognized the critical importance of the ketone in maintaining electronegativity in the lactone ring (Supplemental Fig. S8). Lingrel and co-workers later identified the AAs that contact ouabain within the binding site and influence ouabain affinity (104) (see above, *Cloning the NKA and Identifying the Ouabain-Binding Site*). The structures of dogfish shark rectal gland and pig kidney α1 NKA with ouabain, digoxin, bufalin, and MBG bound have been determined by X-ray crystallography, cryo-electron microscopy (cryo-EM), and modeling (Fig. 1, *D* and *E*), and the CTS-bound structures have been compared (42, 55, 120–122, 202, 356–358).

Despite the differences in these CTS (Fig. 1, *A* and *B*), all four bind to, and stabilize, the phosphoenzyme conformation, E2P, of the NKA α subunit and there is a “general structural similarity” of the CTS-NKA complexes (Fig. 1, *D* and *E*) (120, 121). Ouabain/EO and other cardenolides preferentially bind to and stabilize the E2P conformation and are sensitive to antagonism by high K^+^ (202, 358, 359), whereas bufadienolides appear to be much less E2P-selective (55, 356, 358). Cryo-EM indicates that ouabain binds by a “conformational selection” mechanism (122) in which the E2P conformation changes very little during binding, rather than by an “induced fit” mechanism (360). The CTS insert into, and occlude, the extracellular ion pathway formed by the extra-cellular halves of transmembrane segments αM1-M7. The lactone “head” is inserted first into the “funnel opening” formed by αM1-M2 and αM4–M7, and is situated close to the K^+^ binding sites; the sugar moiety “tail” (absent from bufalin) extends back toward the external aqueous solution (Fig. 1, *D* and *E*) (120, 357). The sugar residues increase CTS affinity relative to their respective aglycones (55, 358) and help stabilize the CTS-NKA complex; the hydroxyl groups on the β face of the steroids hydrogen bond with the polar side chains of αM1-M2 and αM6 (357). Ouabain, but neither digoxin nor bufalin, forms hydrogen bonds with either Gln111 or Glu117 in the αM1-M2 loop, and the extracellular portion of the M1-M2 loop apparently moves slightly closer to the CTS binding site when bufalin or digoxin is bound, than when ouabain is bound (120). Perhaps because of their smaller (5-member) lactone rings, ouabain and digoxin fit ∼5 Å deeper into the pocket, closer to the cation binding sites, and bind better to the E2P conformation than bufalin or MBG (55, 358). The C_14_-C_15_ epoxide of MBG stiffens the steroid core and prevents it from forming an H-bond with Thr797 in M5 that is typical of all the CTS with a C_14_-OH (55, 358). In the ouabain-sensitive α1 NKA, 16 AAs contact bound ouabain, whereas digoxin and MBG have only 13 and seven AA contacts, respectively (55). The binding of CTS to the N-terminal region (i.e., M1 and M2) and to the more central M4–M7 helices hinder the conformational changes required for cation transport (199).

When α1 NKA is made ouabain-resistant by the Gln111Arg and Asn122Asp substitutions located at the N-and C-terminal ends of the M1-M2 loop, respectively, the charged Arg and Asp form a hydrogen bond that limits CTS access to the binding pocket. As a result, ouabain does not penetrate as deeply into the pocket (Fig. 1*E*) and the number of AA contacts is reduced to five for all three CTS, ouabain, digoxin, and MBG (55). These alterations undoubtedly account for the ouabain-resistant isoform’s greatly reduced affinity for CTS.

The NKA α subunit is a docking station for an increasing number of proteins that participate in EO-activated signaling (see *The CTS-Activated “Signalosome”*). The list includes Src kinase, phosphoinositide-3-kinase (PI3K), and the inositol trisphosphate receptor (IP3R) (54). Nevertheless, detailed information about the interaction between the NKA α subunit and its protein binding partners is lacking. Although several groups reported direct interactions between some CTS and nuclear receptors, the concentrations required for these effects (generally in the 0.1–10 µM range) (361, 362) seem unlikely to be present in nuclei under physiological or even pathophysiological conditions (see Supplemental Fig. S9).

It seems doubtful that the small structural differences described above can explain the differences in the function of ouabain, digoxin, and the bufadienolides. Molecular dynamics simulations of the CTS-NKA complexes as well as details of NKA-PK interactions may be needed to resolve the issue. In this regard, ouabain displays a marked flexibility of the steroidal A ring and, when ouabain binds to the NKA, flexion of the A and B rings is crucial for high-affinity binding (363). This raises the possibility that dynamic synchronization between flexion in the ouabain A ring and its NKA binding site may be a critical determinant of ouabain’s selective actions including those related to signaling.

The X-ray-based structural studies confirm that each α protomer has only one CTS binding site and spatially locate the specific residues in the α subunit to which ouabain and other CTS bind (55, 122, 202). The resolved structures do not provide insight into the paradoxical antagonism between ouabain and digoxin or the stimulatory effects of ouabain and other CTS; both effects require multiple interacting sites.

On a related topic, in primary sensory neurons, the ouabain-NKA-Src complex apparently is involved in transducing signals from opioid-like receptors to NaV1.8 channels (364). In this system, ouabain has an antinociceptive effect (365) and it has been suggested that the rhamnosyl residue of ouabain is critical because ouabagenin did not activate signaling (366). Although it has long been recognized that the rhamnose moiety contributes to the high-affinity binding of ouabain to NKA, e.g., Ref. 357, this appears to be the first claim that the sugar moiety in ouabain has a specific role in signaling. The same authors also suggest, based upon modeling studies of the polyhydroxylated steroid cores in ouabain and ouabagenin, that both steroids might chelate Ca^2+^ ions and that the resultant NKA bound complexes lead to calcium-related ionic interactions with Glu-116 and Glu-117 (366). We suspect, however, that Ca^2+^ chelates in the circulation are likely to be relatively unstable. In most instances where ion adducts of ouabain/ouabagenin have been detected in mass spectrometry, the steroid nucleus readily forms stable adducts with H^+^, Li^+^, or Na^+^, and not larger metals such as K^+^ or Ca^2+^, e.g., Refs. 115, 118, 209, 253, 254.

### Mechanism of Ouabain-Digoxin Antagonism and Ouabain Stimulation

**Figure 5.**
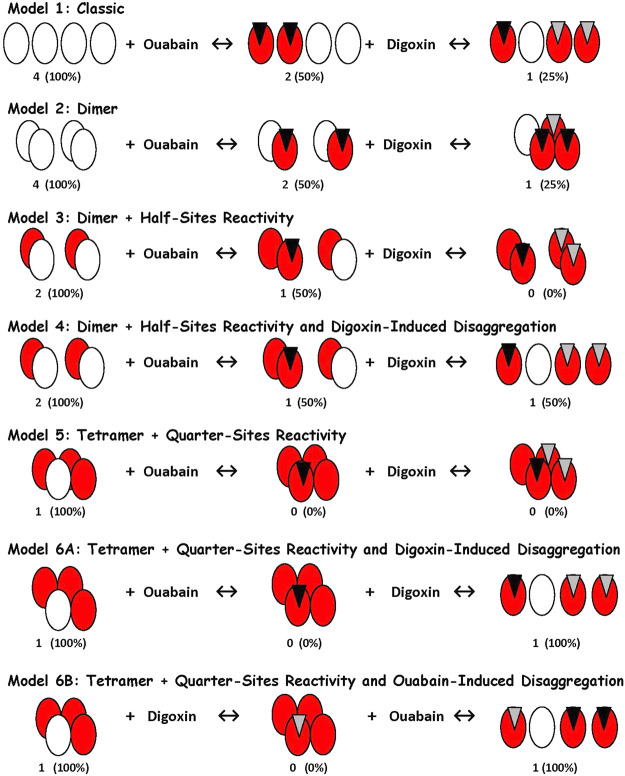
Models for the interaction of ouabain and digoxin with the Na^+^ pump. In all models, four α2 Na^+^ pump subunits are shown for consistency. White and red ovals denote active and inactive α2 subunits, respectively. For simplicity, in Models 1–4, each reaction initially involves addition of one CTS (usually ouabain, black triangle) at its EC50, so that, at steady state, exactly one half of the subunits are inactivated (residual pump activity = 50%). Addition of a second, structurally different, CTS (e.g. digoxin, gray triangle), at a concentration approximately twice the EC50 for ouabain, is then shown. Model 1 uses monoprotomers, while Models 2–4 invoke diprotomers, either without (Model 2), or with (Models 3 and 4), half-of-the-sites reactivity. As illustrated, none of those models can display ouabain antagonism. Models 5 and 6 invoke tetraprotomers each with quarter-site reactivity. In the latter models, binding of one CTS molecule to a single tetramer is sufficient to stop all ion pumping. Models 4 and 6 invoke disaggregration of complexed subunits upon addition of a second CTS. In each case, the relative ion-pumping activity after addition of the first and second CTS (as a percentage of baseline) is shown below each model. In Model 6A, the binding of a single ouabain molecule (inverted black triangle) to an active protomer (white subunit, 100% activity; red subunits are inactive) fully inhibits the NKA (red subunit with inverted black triangle, 0% activity). Subsequent binding of one or more digoxin molecules (inverted gray triangles) to an unbound protomer(s) then causes the tetraprotomer to disaggregate and one of the protomers (white subunit) recovers 100% activity, i.e., ouabain-digoxin antagonism. Conversely, in Model 6B, binding of a digoxin molecule to one of the four protomers also inhibits the NKA; subsequent binding of one or more ouabain molecules to an inactive protomer(s) causes the tetraprotomer to disaggregate and one of the protomers (white subunit) to recover 100% activity. CTS, cardiotonic steroid. Reproduced from Ref. 61 with permission.

Much evidence indicates that NKA pumps can operate as independent αβ protomers (references in Ref. 63) and that, when a CTS molecule is bound, cation transport is blocked. This “classic” functional mode, however, cannot display ouabain-digoxin antagonism (Fig. 5, Model 1) or ouabain-induced stimulation (Fig. 2, Supplemental Fig. S2). These phenomena require multiple interacting CTS binding sites as elucidated by experiments on NKA enzyme preparations that explored the stoichiometric interactions of various NKA substrates. Those and related studies showed cooperative substrate-related interactions between α subunits (296). They also revealed that the functional NKA could be an αβ diprotomer, (αβ)_2_, or a tetraprotomer, (αβ)_4_ (367, 368), wherein highly cooperative inter-α subunit interactions culminated in half- and quarter-site reactivity, respectively (e.g., Refs. 63, 64, 296, 319). By definition, a tetraprotomer contains four CTS binding sites.^7^ As we will now show, the interaction among these four sites as well as the occupation of one of these sites by an eCTS provides the most plausible mechanism underlying the stimulatory effect of ouabain and other CTS, as well as the peculiar interactions between ouabain and digoxin in vivo and in vitro. Analysis of this interaction also provides new hints into the physiological and therapeutic roles of the endogenous and exogenous CTS and highlights the amazing complexity of the CTS-NKA interactions and their consequences.

NKA αβ protomers can form diprotomers (64, 369) in which one (half-sites reactivity; Fig. 5, Models 3 and 4) or both (Fig. 5, Model 2) of the protomers in the pair may be active. But, blocking one protomer of the diprotomer pair with ouabain and then binding digoxin to the other, or displacing the ouabain by digoxin also cannot give rise to ouabain-digoxin antagonism, i.e., cannot increase net NKA activity (Fig. 5, Models 2–4). The NKA also can form stable tetraprotomers (60, 63, 368, 370). With all-sites reactivity (not shown), or even with quarter-sites reactivity but without disaggregation (Fig. 5, Model 5), tetraprotomers do not exhibit ouabain-digoxin antagonism. Further modeling arrived at a mechanism in which there were tetraprotomers with quarter-site reactivity. This model, too, was insufficient to generate stimulation unless digoxin could induce disaggregation of ouabain-bound tetraprotomers or alternatively, ouabain could induce disaggregation of digoxin-bound tetraprotomers, (Fig. 5, Models 6, A and B, respectively) (61). The conclusion is that, in vivo, a large portion of NKA protomers aggregate and function as tetraprotomers with quarter-site reactivity. Fortuitously, direct evidence supporting this model was published in 2007 but was largely ignored; radiation inactivation analysis of human erythrocyte NKA revealed that the target size is consistent with αβ tetraprotomers and their dissociation by digitoxigenin (367).

A question we have not yet addressed is: How can we account for the apparent difference in ouabain-binding kinetics between the biochemical experiments on purified PM preparations and the physiological experiments (*Anomalies of Ouabain Kinetics*)? To our knowledge, this discrepancy has not been investigated. There are two general possibilities. One is that the difference in kinetics might simply be the result of different ligand concentrations for the in situ NKA versus that in isolated membranes, for example, higher concentrations of uncomplexed ATP in situ. Alternatively, the rates of CTS-NKA interaction might depend upon the protomer aggregation: slow binding/unbinding when the protomers are dissociated, and much faster when, as in the physiological experiments, most protomers may be associated into di- and tetra-protomers.

### What Happens In Vivo?

Starting from the simplest situation in vivo where there is a mixture of protomers, diprotomers, and tetraprotomers, and assuming no eCTS are bound, then the addition of either ouabain or digoxin in incrementing amounts will lead only to inhibition, i.e., the upper curve in Fig. 2*B*. Now, consider a more complex situation where we assume the presence of two distinct eCTS, one ouabain-like and the other digoxin-like. We assume that these two eCTS are both present at well below their respective EC_50_ values, that they are present in equal concentrations, and that they have similar binding affinities. We can then anticipate that a portion of all protomeric forms of the NKA will be complexed with a digoxin-like eCTS and that the measurement of the NKA activity reflects only active protomers that are uncomplexed with eCTS. This is the starting (control) situation in Fig. 2*B* for the lower curve. At this point, the addition of ouabain in increasing concentrations will lead to inhibition of protomers and diprotomers, but activation (stimulation due to disaggregation) of tetraprotomers originally bound with digoxin-like eCTS. The net result shown in the lower curve in Fig. 2*B* (and see Fig. 5, Model 6B) is a stimulation of NKA activity that, depending on the dose of ouabain used, may be transient. The measured activity is the sum of the inhibition and stimulation reactions and it is apparent that, at the incubation time employed in this experiment (88) and the low concentrations of ouabain, the stimulatory effect dominates the reaction. With further reaction time in this model, both curves shown in Fig. 2*B* will shift leftward, probably by an order of magnitude and the higher concentrations of ouabain (especially 3–100 nM) will become inhibitory. The major question is: What happens with concentrations of ouabain between 10 pM and 1 nM? Does net stimulation persist at these low concentrations? Some of the results in Table 2 hint that it may last for many hours. To persist indefinitely, the relevant tetraprotomeric forms would need to be regenerated at a rate at least equivalent to the disaggregation shown in Fig. 5. Significantly, the low stimulatory concentrations of ouabain overlap the physiological range of the eCTS as measured by various immunoassays (8) (Supplemental Fig. S9). Thus, variations in the circulating and local tissue concentrations of eCTS well below 1 nM would appear to be the most physiologically important in terms of effects (14, 371). Note, also, that, in vivo, both ouabain- and digoxin-like eCTS will be bound to tetraprotomers. In the example given above, only the impact of ouabain was considered on a tetraprotomer in which a digoxin-like eCTS was bound. In reality, there also will be tetraprotomers with ouabain-like eCTS bound and the addition of digoxin will stimulate their activity. In turn, the relative amounts of ouabain-like and digoxin-like eCTS in the circulation will determine the respective proportions of their tetraprotomer complexes (see Supplemental Section 2 and Fig. S2).

Importantly, monovalent cations influence both the eCTS binding and the association of protomers. As mentioned above, washing minimally purified NKA in its near-native membrane environment with a high K^+^, but not high Na^+^, solution promotes eCTS dissociation likely from a tetrameric NKA, presumably by accessing the cation-binding site in the inhibited E1P conformation from the cytoplasmic side (see Supplemental Section S1). But, in the presence of detergent and under CTS-free conditions, a high K^+^ solution favors protomer aggregation, whereas high Na^+^ favors dissociation of the polyprotomers to monoprotomers (370).

Thus far, our focus has been largely on the NKA’s cation-pumping function. The revolutionary discovery that the NKA is also a hormone receptor and signaling molecule has received only passing mention here, even though this is essential for understanding the full impact of the NKA on physiology and pathophysiology that are covered in the final sections of this review. We therefore next address aspects of the NKA as a signal transducer.

### The CTS-Activated “Signalosome”

**Figure 6.**
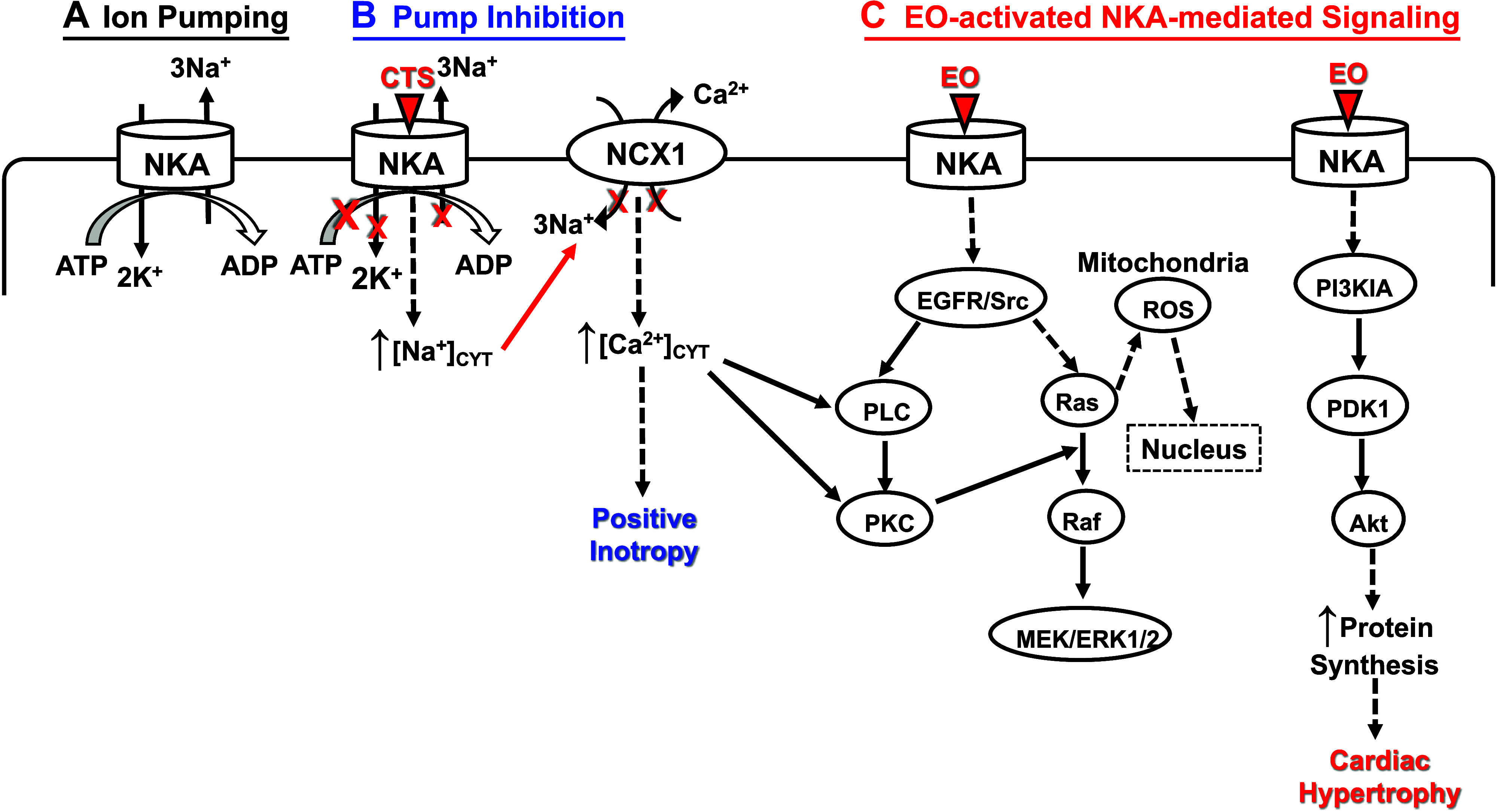
Signaling pathways involved in cardiotonic steroid (CTS)-mediated positive cardiac inotropy and ouabain-activated cardiac hypertrophy. *A*: normal operation of the NKA (Ion Pumping). *B*: acute NKA inhibition by any cardiotonic steroid (“Pump Inhibition”) raises cytosolic Na^+^ ([Na^+^]_CYT_), thereby inhibiting Na^+^/Ca^2+^ exchanger-1 (NCX1)-mediated Ca^2+^ extrusion (and promoting NCX1-mediated Ca^2+^ entry - not shown). The result is a net rise in cytosolic Ca^2+^ ([Ca^2+^]_CYT_), enhanced Ca^2+^ signaling and, in the heart, positive inotropy. *C*: sustained NKA block by (endogenous) ouabain (EO) activates protein kinase cascades, which lead to reactive oxygen species (ROS) generation, altered protein expression, altered Ca^2+^ signaling (not mediated by NCX1) and, in the heart, cardiac hypertrophy. 335. NKA, Na^+^, K^+^-ATPase (Na^+^ pump). The model is modified from Fig. 10 in Ref. 334 and Fig. 11 in Ref.

Following their seminal studies on ouabain-activated PK signaling in the late-1990s (6, 7), Xie and Askari discovered that ouabain can also trigger the generation of reactive oxygen species (ROS) in primary cultured cardiomyocytes (103). They and others extended their findings on PK cascade signaling to numerous cell types and elucidated several CTS-activated signaling pathways that may be cell type-specific (372). These pathways may involve Na^+^-dependent or Na^+^-independent mechanisms (334, 335) and may be selectively activated by specific CTS species (e.g., ouabain, digoxin, or bufalin) (117, 347, 373) although most have only been tested with ouabain. Four examples are *1*) the NKA-Src-PLCγ-InsP_3_R cascade that may modulate Ca^2+^ release from the sarco/endoplasmic reticulum and opening of l-type Ca^2+^ channels (374). *2*) The NKA-Src-EGFR-Ras-Raf-MAPKK-ERK1/2 cascade that may regulate the renal tubule cell apical membrane Na^+^/H^+^ exchanger, NHE3, and, thus, Na^+^ reabsorption (375, 376). *3*) The NKA-PI3K1A-PDK-Akt pathway that may be associated with ouabain-induced cardiac hypertrophy (Fig. 6) (334, 344, 377). *4*) Aperia and colleagues reported that the ouabain-NKA complex interacts with the inositol trisphosphate receptor to activate NF-κB and induce [Ca^2+^]_CYT_ oscillations (378, 379).

To participate in these numerous signaling activities implies that the NKA must interact directly and indirectly with numerous protein molecules. Xie tackled this issue by invoking the concept of the “signalosome” (372). Accordingly, the NKA functions as a “scaffold protein,” a molecular device that transiently assembles transducing molecules and activated (ligand-bound) transmembrane receptors in dynamic clusters (380). The NKA signalosome serves as a docking station presumably because the NKA cluster has a high concentration of low-affinity protein-binding sites (54). The dynamic nature of the cluster assembly and disassembly should prevent inadvertent signal transduction due to fluctuations in the cytosolic concentrations of the transducers because transduction will not occur unless the NKA receptors are activated and clustered in the signalosome (380).

Signalosome assembly has been elucidated in the case of other receptors (e.g., Refs. 380–382) but few details of NKA signalosome assembly have been investigated. For example, whether protomers, diprotomers, and/or tetraprotomers cluster in signalosomes is unknown, as are differences in clustering among the α subunit isoforms and whether these polyprotomers are homo- or heteropolyprotomers. Also, does the specific β isoform influence cluster formation? Investigations into the identity of the numerous transducer proteins that can bind to, or cluster with, NKA (54) have not necessarily accounted for the difficulties caused by the dynamic nature of the assemblies. Moreover, because NKAs are present in virtually all cells and, as indicated above, the cluster composition is cell type- and ligand-specific, the number of possible combinations is enormous.

The precise mechanisms by which CTS activate the signaling pathways are still uncertain. Based on a series of studies performed mainly on pig kidney LLC-PK1 cells transformed by replacement of ouabain-sensitive (S) with ouabain-resistant (R) α1 NKA, Xie and co-workers described a number of observations about ouabain-activated, NKA-mediated PK signaling (383–388). *1*) There is a nonpumping (“silent”) pool of α1, but not α2, NKA, located in caveolae (385, 388) that binds Src at NKA cytosolic domains (CD-) 2 and CD-3 (384, 386). The latter is at the N-terminus of the nucleotide-binding domain (red region of the NKA protomer large M4-M5 cytoplasmic loop in Fig. 1*D*). *2*) The α1 NKA-Src complex is the functional, ouabain-triggered signaling complex; ouabain binding releases inactivated Src, thereby initiating Src activation and PK signaling (383). *3*) A 20-AA peptide, termed “NaKtide,” derived from the α1 NKA CD-3 sequence, can bind Src and thereby antagonize ouabain-induced signaling. The α2 CD-3 sequence, which differs from α1 by three AAs in both humans and rodents (376), neither binds Src nor antagonizes signaling (386). *4*) Rat α2^R^ NKA transfected into α1^S^-deficient pig kidney proximal tubule cells can pump cations but not mediate ouabain-triggered signaling (387).

This seemingly straightforward signaling mechanism proposal raises a number of concerns. First, other investigators report that the caveolar and noncaveolar NKA pools are indistinguishable in terms of function (389, 390). If not binding to caveolin-1 (388), however, what determines NKA clustering in signalosomes? This issue may be moot, however, if only CTS-bound (i.e., by definition, nonpumping) NKA molecules, be they monoprotomers or tetraprotomers with quarter-site reactivity, are able to form signalosome clusters and/or there is a reserve sub-PM pool of NKA as in skeletal muscle (43). Second, it is surprising that Xie and colleagues studied transformed renal cells expressing R-α1 and R-α2 NKA rather than S-α1 and S-α2 in most of their experiments, and that they needed at least 0.1, and usually 1.0–100 µM ouabain (i.e., toxic concentrations) to activate signaling. Third, their use of renal cell lines, primarily, seems in conflict with the aforementioned evidence that EO-activated signaling is cell type-specific. Do most renal cells, which express very little or, perhaps, even no α2 or α3 (33, 34), have the machinery to initiate signaling via α2 NKA? Fourth, the view that Src interacts directly with α1, but not α2, NKA (376) is controversial (221, 391) and a few groups have been unable to replicate Xie’s finding (392–394). Picomolar or low nanomolar concentrations of CTS surely activate Src signaling via α2 in rodents in vivo because rodent α1 NKA is ouabain-resistant (117, 395). This seems clear from the experimental evidence discussed in the next few sections, namely, that EO-triggered signaling in mice is greatly attenuated or abolished by replacement of S-α2 by R-α2 NKA.

The following concerns and controversies exemplify some of the other unsettled issues also raised by numerous studies of CTS-NKA-linked signaling. *1*) The concentrations of ouabain used in many in vitro signaling studies exceed 0.1 or 1 µM (14, 332, 334, 396, 397) (see Supplemental Fig. S9*A*), two or more orders of magnitude higher than the EC_50_ of ouabain-sensitive NKAs. The physiological relevance of such studies must be questioned. In humans, normal physiological plasma EO is in the range of ∼0.05–0.6 nM, the pathophysiological range is ∼0.4-2 nM, and higher levels are toxic/lethal (Supplemental Fig. S9*B*); these ranges are a little higher in rodents, where α1, but not α2–α4, is ouabain-resistant (8). *2*) CTS species-specific, or biased, actions (e.g., ouabain vs. digoxin or bufalin; see Figs. 2 and 3, and Supplement Figs. S4, S5, and S6) (96, 117) are usually ignored. Ouabain has been used in the vast majority of studies on NKA-mediated signaling. A very few studies have shown that bufalin (398) and digoxin (392) also activate Src but we are unaware of a systematic comparison of the selective actions of the different eCTS on NKA-mediated PK signaling pathways. *3*) It is crucial to address cell-type specificity. Also, how well do studies on cell lines (385) apply to primary cultured cells and cells freshly derived from live animals (389)? *4*) Does the genetic background of different mouse lines influence signaling responses? *5*) Finally, the FBS and horse serum routinely used for tissue culture contain EO (224) and, perhaps, other eCTS. Care must be taken to serum-starve cell cultures for a sufficiently long period (e.g., Refs. 103, 344) to avoid confounding results because of the presence of EO in the presumably ouabain-free “controls.”

With this new insight into CTS-NKA interactions, it is appropriate to ask the critical question: Does stimulation of the NKA by eCTS or exogenous CTS have any physiological and or pathophysiological significance? There are at least five situations where phasic changes in the concentrations of circulating eCTS may be of interest. They are the “pre-conditioning phenomenon,” bipolar disorder, blood pressure control, pregnancy, and the impact of digoxin therapy. Consider the preconditioning phenomenon (the other situations will be covered in subsequent sections of this review), transient exposure of brain and cardiac tissue to ouabain (i.e., “preconditioning”) protects the tissue from ischemic damage; in some instances, stimulation of the NKA by CTS has been linked directly with its protective effects (299, 399, 400). A similar mechanism was proposed for the beneficial effects of oral dosing with ouabain on the failing human heart (401).

### A Stimulating New Twist: Effects of Antibodies Raised against the M7-M8 and M1-M2 Loops

Although perhaps only indirectly related to ouabain-binding site-eCTS interaction, Kai Xu’s largely overlooked experiments with antibodies raised against two NKA extracellular loops may shed new light on the oligomer dissociation mechanisms we just discussed. She examined the role of the α1 NKA M7-M8 loop, using polyclonal antibodies (pAb) raised against the rat Asp892-Arg906 sequence (see Footnote No. 3 and Fig. 1*C*) in the C-terminal half of the loop; the AA sequence is identical in human, rat, dog, sheep, and pig α1 NKA (111). The pAb cross-reacted with rat and dog α1, and with rat α2 and α3 in which the 15 AA sequence is 87% and 93% conserved, respectively, as it is in human α2 and α3. The rate of ATP hydrolysis by purified rat cardiac and dog kidney NKA (both predominantly α1) pretreated with the pAb increased by as much as a factor of 3.1–3.5 versus the control (Supplemental Section S3 and Fig. S3*A*). Regrettably, the effect on cation transport was not measured. This leaves open the question of whether these antibodies simply uncouple ATP hydrolysis from ion transport or whether there is an alternative and more intriguing explanation as described below.

When incubated with ^32^P-ATP, the ^32^P labeling of anti-M7-M8 loop pAb-pretreated rat and dog NKA preparations was >300% of controls (111). Treatment of primary cultured rat cardiomyocytes with the pAb did not affect the cytosolic Na^+^ concentration (402) but, nevertheless, increased Ca^2+^ transients by 24% and contraction amplitude by 67% (111). IV infusion of the pAb into normal mice in vivo exerted a cardiotonic effect; for example, it increased ejection fraction and cardiac output and decreased end-systolic volume (402). The anti-α1 M7-M8 loop pAb did not interfere with ^3^H-ouabain binding to rat cardiac sarcolemmal vesicles, whereas pAb raised against the rat α1^R^ M1-M2 (Arg111-Asp122) loop reduced ouabain binding by one-third (111).

How can we explain the very large anti-α1 M7-M8 loop antibody-induced increases in ^32^P-labeling and ATP hydrolysis rate? Such large increases would surely not be anticipated after a bulky antibody protein binds to NKA protomers. The observations could, however, readily be explained both qualitatively and quantitatively if *1*) much of the rat and dog cardiac membrane NKA was clustered in tetraprotomers with quarter-site reactivity and *2*) the antibody binding facilitated dissociation of tetraprotomers into a mix of functional monoprotomers and diprotomers (see Fig. 5, Models 6 A and B, and Supplemental Section 3 and Fig. S3*B*).

The cardiotonic effect in the absence of NKA inhibition or a rise in cytosolic Na^+^ also requires explanation because this seemingly eliminates or greatly reduces the involvement of an NCX-mediated increase in cell Ca^2+^. Xu and co-workers showed that the anti-α1 M7-M8 loop pAb increases whole cell l-type Ca^2+^ channel (LTCC) currents in freshly isolated rat ventricular cardiac myocytes (403). This activation did not involve the well-known LTCC regulatory mechanisms: the adrenergic nervous system, protein kinase-A (PKA), or Ca^2+^-calmodulin kinase II (CaMKII) (403). The antibody-augmented Ca^2+^ entry was completely blocked by the LTCC antagonist, nifedipine, and was unaffected by NCX antagonist, KB-R7943 (rostafuroxin); in contrast, ∼70% of the ouabain-augmented Ca^2+^ entry was blocked by KB-R7943, and only ∼30% by nifedipine (404). The antibody-augmented Ca^2+^ current increase was mediated by a signaling cascade involving Src and ERK1/2 (403). This raises the likelihood that antibody binding to the NKA, like the binding of ouabain, impedes NKA conformational rearrangement during cycling and operates through the signalosome to activate, slowly, Src-mediated PK cascades in the cardiomyocytes (see *The CTS-Activated “Signalosome”*).

Polyclonal antibody raised against the rat α1^R^ M1-M2 loop (Arg111-Asp122; see Footnote No. 3) also immunoprecipitated rat α2^S^ NKA (405). The anti-M1-M2 pAb, like the anti-M7-M8 pAb, augmented cytosolic Ca^2+^ transients and cell shortening in isolated rat ventricular cardiomyocytes and induced a cardiotonic effect in vivo (405). In immunofluorescent experiments, both the anti-M1-M2 loop pAb and a monoclonal antibody (mAb) raised against the same α1^R^ M1-M2 loop sequence were bound to the PM external surface of cultured rat cardiomyocytes or CV-1 monkey kidney cells that, respectively, express α1^R^ and α1^S^ NKA. Immunofluorescence cytochemistry showed that these mAb could be largely displaced by very high concentrations (1–5 mM) of ouabain (405, 406), suggesting that the antibodies might not bind ouabain-bound NKA. This, plus the evidence that ouabain could displace anti-M1-M2 pAb from the PM (111), implies that the antibodies and ouabain apparently compete for binding and that both cannot be bound at the same time.

The anti-M1-M2 mAb increased the rate of ATP hydrolysis by normal rat and dog heart NKAs by ∼80%. Anti-M1-M2 mAb and pAb both increased the ATP hydrolysis rate of NKA obtained from failing rat hearts by a factor of ∼1.7–1.9 (406). In ouabain dose-response curves on normal rat and dog heart NKAs, the magnitude of the stimulation declined as the ouabain concentration increased (406), supporting the view that the anti-M1-M2 antibodies cannot bind to (or stimulate) ouabain-bound NKA. The stimulation of ATP hydrolysis by both anti-M1-M2 and anti-M7-M8 antibodies is consistent with the idea that both antibodies probably promote dissociation of NKA tetraprotomers with quarter-site reactivity, and, perhaps, diprotomers with half-site reactivity (see Supplemental Section 3 and Fig. S3*B*). Furthermore, by impeding NKA conformational rearrangement, the bound antibodies may activate the NKA signalosome complex.

With this insight into the cellular/molecular functions of EO and other eCTS, CTS-induced NKA stimulation and ouabain-digoxin antagonism, we now turn our attention to some of the numerous physiological and pathophysiological consequences of these interactions.

## THE PHYSIOLOGY AND PATHOPHYSIOLOGY OF CTS-NKA INTERACTIONS

### Genetic Engineering Provides Some Strategic Models

Lingrel and colleagues, beginning in the late 1990s, followed up their identification of the NKA α and β subunit isoform sequences and discovery of the AAs responsible for ouabain binding (16, 89) with genetic engineering studies on the α subunit. Initially, they studied the specific physiological roles of the various NKA isoforms in mice. To this end, they deleted the α1 and α2 isoform genes to generate haplo-insufficient and knockout (KO) mice (37, 407, 408) and also induced tissue-specific α isoform deletions (116, 409, 410). They then used a knock-in strategy to generate tissue-specific α1 and α2 transgenic (TG) overexpressor mice (411, 412). Finally, they mutated AAs 111 and 122 in the α1 and α2 isoforms to alter ouabain affinity (Fig. 1, *C* and *E*). The substitution of neutral for charged AAs, Arg111Gln and Asp122Asn, renders mouse α1 NKA ouabain-sensitive (α1^S^); conversely, replacement of hydrophobic or neutral AAs by charged AAs, Leu111Arg and Asn122Asp, renders mouse α2 NKA ouabain-resistant (α2^R^). The specific roles of the NKA α subunit isoforms have been reviewed elsewhere (e.g., Refs. 8, 413); here, we focus on the ouabain-binding site and endogenous ligands.

Lingrel, reportedly, had been very skeptical of the view that hypertension is linked to EO.^8^ Partly with this in mind, he and his collaborators generated α2^R^ mice, genotype α1^R/R^α2^R/R^ (414), and “SWAP” mice with genotype α1^S/S^α2^R/R^ (415), versus wild type (WT) α1^R/R^α2^S/S^ mice. They also generated cardiac-selective α2 NKA-KO mice (C-α2^S/S^-KO) (116) and cardiovascular-selective α2 NKA-KO mice (CV-α2^S/S^-KO) (410). Their aim was to determine if the near-total prevention of CTS binding to the NKA α1 and α2 isoforms had little or no biological significance; by analogy, the same conclusion would extend automatically to their endogenous ligand(s). As we shall see in the ensuing two sections, the results proved beyond doubt that the NKA ouabain-binding site and, thus, its eCTS ligands are of direct relevance to hypertension and HF, and to biology in general.

### The NKA Ouabain-Binding Site-EO-BP Connection

The observation that sustained administration of ouabain to normal rats usually induces hypertension (Fig. 3*A*) (94) has now been replicated in numerous laboratories, e.g. Table 3 and Refs. 107, 416, 432–434. Ouabain infusions of ∼10–30 µg/kg/day, which elevate BP, produce a sustained circulating ouabain level in the range of ∼1–5 nM (435). A few groups have been unable to induce hypertension with ouabain (see Supplemental Tables S1, S2, and S3 in Refs. 416) but one explanation may be that ouabain sensitivity is under genetic control: ouabain-sensitive (α1^R/R^α2^S/S^) and ouabain-resistant (α1^R/R^α2^R/R^) rat strains can be generated by inbreeding rats for 5–6 generations (431). Another explanation is the aforementioned presence of antihypertensive digoxin-like CTS in some laboratory chows.

**Table 3.** NKA ouabain-binding site-endogenous ouabain-hypertension links

Ouabain- and ouabain analog-induced hypertension:			
Animal	Ouabain or Analog Administered	Route	References
Rat	Ouabain; ouabagenin (not digoxin or digitoxin)	SC or IP	Fig. 3A (94, 96, 326, 416)*
Rat	Ouabain + high dietary salt	SC	(328)
Rat	Dihydro-ouabain; ouabagenin; iso-ouabain; ouabain with open lactone ring	SC	(326)
Rat	Ouabain	ICV	(118)
Mouse	Ouabain	IP	(414)
Mouse	Ouabain	ICV	(417)

Hypertension studies in which elevated plasma EO levels have been measured:		
Hypertension	Assay Method	References
SC Ang II + HS (rat)	Anti-ouabain RIA	Fig. 8B (418)
ICV Ang II (rat)	LC-RIA & LC-MS-MS	Fig. 8D (118)
Dahl salt-sensitive (rat)	LC-MS-MS	Fig. 7D (419)
ACTH-induced hypertension (mouse)	Immunoassay using Digibind**	Fig. 9B (109)
ACTH-induced hypertension (rat)	Anti-ouabain immunoassay	(260)
Ectopic corticotropin syndrome (human)	Anti-ouabain immunoassay	(420)
DOCA-salt (mouse)	Anti-ouabain immunoassay	(421)
“Essential” hypertension (human)	Anti-ouabain immunoassay	(101, 243, 256)
“Essential” hypertension (human)	Red cell ouabain binding assay	(422)
Milan hypertensive rat strain	Anti-ouabain RIA and radioenzyme assay	(423)
Primary aldosteronism (human)	Anti-ouabain immunoassay	(101, 256, 424,425)
Primary aldosteronism (human)	Red cell ouabain binding assay	(422)
Reduced renal mass + salt (rat)	Anti-ouabain immunoassay	(426,427)
1Kidney-1clip (1k1c) hypertension (rat)	Anti-ouabain immunoassay	(428,429)
Spontaneously hypertensive rat (SHR)	Anti-ouabain RIA	(430)

*Supplemental Table S1 in Ghadhanfar et al. (416), published in 2014, lists 16 reports showing that sustained exogenous ouabain administration elevates BP in rats. That table was incomplete (e.g., Refs. 328, 431), and there have been several subsequent reports.

**Digibind is anti-digoxin antibody Fab that also binds ouabain with high affinity. It was used to measure endogenous CTS in the hydrophilic extract of the plasmas to detect EO and minimize cross-reactivity with common glucocorticoids, mineralocorticoids, and digoxin and MBG-like molecules that are all hydrophobic.

Elevated plasma EO has been measured by MS and by immunoassay with selective anti-ouabain antibodies in rodent and human hypertension, including human “essential” (primary) hypertension and primary aldosteronism (references in Table 3). In patients with primary aldosteronism, the plasma EO level was “normalized” after removal of the adenoma (256, 424). The adenomatous tissue was examined in one study but did not exhibit ouabain-like immunoreactivity (424).

**Figure 7.**
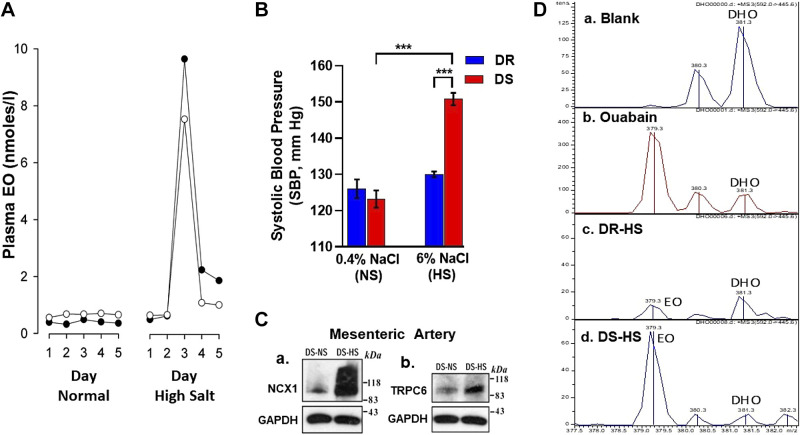
High dietary salt increases plasma endogenous ouabain (EO) in normal human subjects and in Dahl salt-sensitive (DS) rats. *A*: on day “0,” 10 g (171 meq) NaCl was added to the normal salt diet (NS) of normal human males for 5 days (= high salt, HS). Endogenous ouabain (EO) was measured on C-18-extracted plasma samples with an ouabain-specific antibody immunoassay (229) for 5 days before the dietary salt increase and during the 5 days on HS. HS led to a 13-fold increase, on average, in plasma EO on day 3 (5.8 ± 2.2 nM; *n* = 13, *P* <0.05), after which the EO level fell to an elevated plateau relative to the level during NS. Data from two representative subjects are shown (○ and ●, respectively). Reproduced from Ref. 227 with permission. *B–D*: an HS diet increases plasma endogenous ouabain (EO), arterial myocyte Ca^2+^ transporter expression, and systolic blood pressure (SBP) in DS rats. Dahl salt-resistant (DR; *n* = 5) and DS (*n* = 7) rats were initially fed a 0.4% NaCl (NS) diet and SBP was measured by tail cuff for 5 days before switching to a 6% NaCl (HS) diet. *B*: graph shows the mean SBP in the rats for the 5 days on the NS diet and for days 7–11 on the HS diet, when the SBP had reached a plateau. ***P < 0.001 for the indicated pairs. *C*: representative immunoblots indicate that the HS diet, elevated plasma EO, and the high BP in DS rats on the HS diet are all associated with enhanced expression of the Ca^2+^ transporters, Na^+^/Ca^2+^ exchanger-1 (NCX1), and transient receptor potential channel-6 (TRPC6). This is also seen after prolonged ouabain treatment of arterial myocytes (Fig. 4, *C*–*E*) and in arterial myocytes from rats with ouabain-induced hypertension (Supplemental Fig. S6*E*). *D*: plasma EO is higher in DS than DR rats on a HS diet. EO was measured in pooled plasma samples following solid-phase extraction by direct spray-tandem multistage mass spectroscopy (SPE-MS-MS-MS) using exogenous dihydro-ouabain (DHO) as an internal reference standard. Samples were monitored for lithiated molecular adducts (M+Li^+^) at 379.2 and 381.2 m/z (mass/charge ratio), respectively. *D*, *a* and *b*: standards: absolute ion intensities are unsuppressed. *D*, *c* and *d*: highly concentrated C18 extracts of pooled plasma samples were spiked with 50 nM DHO; the absolute ion intensities are suppressed by ∼75%. The relevant numbers are the EO/DHO peak ratios: 0.65 for DR-HS vs. 8.50 for DS-HS rats, a 13-fold increase. Data in *B* and *C* reproduced from Ref. 419 with permission; D from the online supplement to Ref. 244.

There is broad consensus that excessive dietary salt is a major factor in the etiology of human hypertension (436, 437). Increasing dietary salt (from 2 to 20 g/day) in patients with essential hypertension increases systolic BP (122–138 mmHg) and elevates eOLC (438). In normal human subjects, the switch from a “normal salt” (NS) to a high-salt (HS) diet for 5 days triggers a large rise in circulating EO that peaks within 2–3 days and then declines to a modestly elevated plateau (Fig. 7*A*) without a change in BP (227). The absence of an effect on BP in this short period is consistent with the delayed impact of peripheral ouabain infusions in normal rats (Fig. 3*A*) or a HS diet in Dahl salt-sensitive (DS) rats, which typically take 1–2 wk to raise BP to a stable elevated level. The HS diet also elevates plasma EO in the DS rats whereas, in Dahl salt-resistant (DR) rats, HS does not raise BP and plasma EO is lower than in DS rats (Fig. 7, *B*–*D*). Importantly, the BP in rats with ouabain-induced hypertension is not salt-sensitive and a HS diet does not alter BP in these animals; thus, the mechanisms downstream of ouabain that raise BP in this model apparently are not impacted by HS (435).

The renin-angiotensin-aldosterone system (RAAS) is critically involved in many aspects of cardiovascular homeostasis. Activation of the RAAS, with augmented Ang II generation, increases sympathetic drive to the heart and arteries and plays a central role in the pathogenesis of hypertension and HF (439, 440). Moreover, high dietary salt and Ang II are convergent signals that increase sympathetic nerve activity (441). In addition, the elevated plasma EO observed in many patients with hypertension may be explained by the fact that Ang II, acting via Ang type 2 receptors, stimulates secretion of ouabain by adrenocortical cells (106). Importantly, all of these mechanisms contribute to the vasoconstriction, increased peripheral vascular resistance, and elevated BP in hypertension. It is not surprising, therefore, that agents that interfere with Ang II generation or action, namely, angiotensin-converting enzyme inhibitors and Ang II receptor blockers, are very effective antihypertensive agents (439, 440, 442, 443).

**Figure 8.**
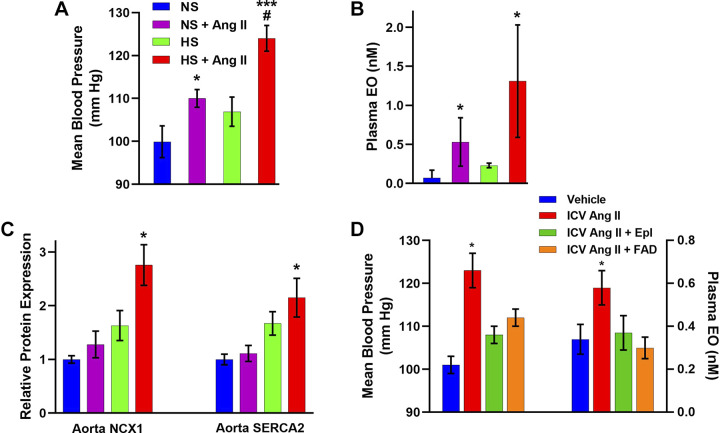
Subcutaneous (sc) low-dose angiotensin II (Ang II) + high dietary salt (HS) increases plasma endogenous ouabain (EO) and expression of arterial smooth muscle Ca^2+^ transporters, and raises mean blood pressure (MBP). Intracerebroventricular (ICV) very low-dose Ang II also raises MBP and plasma EO. *A–C*: Sprague-Dawley rats were fed a normal salt diet (0.4% NaCl, NS) for 1 wk, and then NS or an HS diet (2% NaCl) and infused sc with vehicle or low-dose Ang II (150 ng/kg/min via minipump) for 2 wk. MBP was measured by telemetry (*A*). Plasma EO was measured by radioimmunoassay (RIA) (*B*). Relative expression of aortic Na^+^/Ca^2+^ exchanger-1 (NCX1) and sarco-/endoplasmic reticulum ATPase-2 (probably mostly SERCA2b, the predominant isoform in vascular smooth muscle) was measured by immunoblot (*C*). *P < 0.05, ***P < 0.001 vs. NS; #*P* < 0.05 vs. HS and NS+ Ang II (ANOVA). Data from Ref. 418 regraphed and published here with permission. *D*: *n* = 4 (NS) or 8 rats (all others); intracerebroventricular (ICV) infusion of Ang II (2.5 ng/min) in male Wistar rats for 14 day elevates plasma EO and MBP. Concurrent ICV infusion of eplerenone (Epl, 5 µg/day), a mineralocorticoid receptor blocker, or FAD-286 [FAD, 4-(5,6,7,8-tetrahydroimidazo[5,1-f]pyridin-5-yl)benzonitrile hydrochloride; 25 µg/day], an aldosterone synthase inhibitor, prevents EO and MBP elevation. MBP was measured via a right femoral artery catheter. EO was measured by RIA on solid phase-extracted plasma samples; EO was verified off-line by liquid chromatography-multistage mass spectroscopy (LC-MS-MS-MS) on plasma samples pooled from each of the groups. *P < 0.05 vs. all other groups (two-way ANOVA); *n* = 7 or 8 rats/group. Data from Ref. 118 regraphed and published with permission.

An HS diet enhances mesenteric artery NCX1 and TRPC6 protein expression in the hypertensive DS rats (Fig. 7, *B* and *C*), likely mediated by increased EO and analogous to the effect of ouabain infusion in normal rats (Fig. 4*C*). In normal rats, too, HS plus low-dose Ang II increases plasma EO, upregulates VSM Ca^2+^ transporters, and raises BP (Fig. 8, *A*–*C*) (418). The implication is that elevated EO triggers the VSM Ca^2+^ transporter protein upregulation and, along with increased sympathetic drive, helps elevate BP in normal rats infused with low-dose Ang II and in DS rats on a HS diet.

Additional evidence for a crucial role of EO in the elevation of BP comes from studies on the effects of ouabain antagonism and ouabain immunoneutralization in a variety of rodent hypertension models, in human preeclampsia (Fig. 3*A* and Table 4) and in “essential” hypertension patients with certain genetic profiles. Specifically, some genetic variants of lanosterol synthesis, an enzyme involved in the biosynthesis of the ouabain precursor, cholesterol, have been linked to altered Na^+^ excretion and hypertension (450, 456). The ouabain antagonists used were digoxin, digitoxin (Fig. 3*A*) and rostafuroxin, a derivative of digitoxigenin (457). Importantly, rostafuroxin lowered BP in a number of salt/volume-sensitive forms of hypertension (Table 4) but not in spontaneously hypertensive rats (SHR) (458); digoxin was not tested in this model. Another group, however, reported that serum EO (RIA) was elevated in SHR versus control Wistar-Kyoto (WKY) rats and that renal denervation in SHR lowered the EO to the level in WKY, but BP was not measured (430).

**Table 4.** Infusion of anti-ouabain antibodies or Fab fragments that bind ouabain with high affinity (e.g., Digibind)*, or an ouabain antagonist (digoxin, digitoxin, or rostafuroxin), or primary immunization against ouabain prevents or greatly attenuates BP elevation in hypertension

Rat (Mouse, Human) Hypertension	Agent (SC except as noted)	References
Ouabain	Digoxin; digitoxin	(96)
Ouabain	Rostafuroxin (oral)	(107)
Ouabain + salt	Digoxin	(328)
ACTH	Digibind	(109, 444)
Ang II	ICV Digibind	(445)
Ang II-salt (mouse)	Digibind	(446)
Aortic coarctation	Digibind	(86)
Dahl salt-sensitive rat	ICV Digibind	(100)
DOCA-salt	Digibind	(447)
DOCA-salt	Rostafuroxin (oral)	(448)
ENaC upregulation + Na^+^-rich aCSF	ICV Digibind	(449)
“Essential” hypertension (human) with certain gene variants	Rostafuroxin (oral)	(450)
ICV-infused Na^+^-rich aCSF	ICV anti-ouabain antibodies	(417)
1Kidney-1clip (1k1c) hypertension (rat)	Anti-ouabain antibodies	(428)
Milan hypertensive strain, MHS (rat)	Rostafuroxin (oral)	(451)
Preeclampsia (human)	Digibind	(452)
Preeclampsia model (rat)	Digibind	(453)
Reduced renal mass	Digibind	(454)
Reduced renal mass + salt	ICV Digibind	(455)
Reduced renal mass + salt	Rostafuroxin (oral)	(448)
Reduced renal mass + salt	Autoimmunization against ouabain	(426)
Sino-aortic denervation + salt	Digibind; digoxin	(328)

*See text Footnote No. 8 regarding the controls for these experiments.

The immunoneutralization experiments used anti-ouabain-selective antibodies,^9^ active anti-ouabain antibody generation, or Digibind. The latter is a commercial anti-digoxin Fab fragment preparation that also binds ouabain with high affinity (459, 460). In all the studies listed in Table 4, the antibodies or the antagonists either prevented or markedly attenuated BP elevation. Note that in the ICV infusion experiments, the doses of antibodies or Fab were far too low to be effective in the periphery, thereby demonstrating that hypertension could be attenuated or prevented by immunoneutralizing only the central (brain) EO. This suggests that “brain ouabain,” alone, is sufficient to evoke sustained hypertension; but, as we shall soon see, the story is more complex.

**Figure 9.**
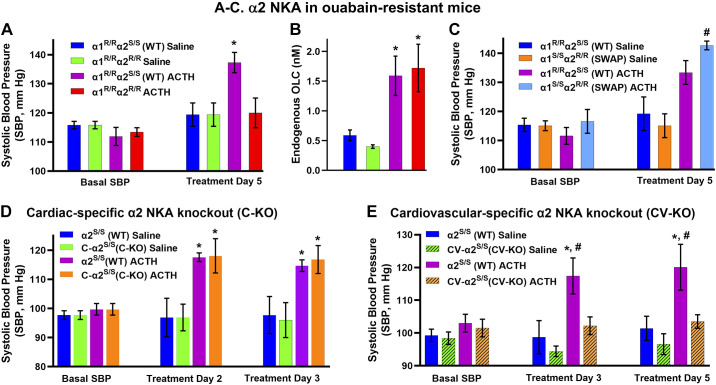
Impact of adrenocorticotropic hormone (ACTH) on endogenous ouabain (EO) and blood pressure (BP) in normal and genetically modified (ouabain-resistant) mice. *A–C*: ACTH injections elevated plasma (endogenous) ouabain-like compound(s) (eOLC) in wild-type (WT; α1^R/R^α2^S/S^) mice and mice with ouabain-resistant α2 NKA (α1^R/R^α2^R/R^) (*B*) but induced hypertension only in the WT mice (*A*). WT and α1^R/R^α2^R/R^ mice were injected subcutaneously with saline (*n* =15 per genotype) or 500 µg/kg ACTH fragment 1–24 (*n* =25 per genotype) for 5 days; systolic blood pressure (SBP) was measured by tail cuff 3 h after each injection. Bars represent means ± SE; *P < 0.05 vs. saline treatment. *C*: when α1 was made ouabain-sensitive in the α2^R/R^ mice (i.e., α1^S/S^α2^R/R^ or “SWAP” mice), ACTH elevated SBP even more than in WT mice. Protocol similar to that for *A*; *n* = 12 mice of each genotype injected with saline and *n* = 30 of each genotype injected with ACTH. #*P* < 0.05 vs. α1^R/R^α2^S/S^ ACTH. Data from Ref. 109 regraphed and published with permission. *D*: mice (C57Bl/6) with cardiac-specific knockout of α2^S/S^ NKA (C-α2^S/S^-KO), like wild-type (WT) mice, develop ACTH-induced hypertension. SBP was measured by tail cuff. Synthetic ACTH (Cortrosyn, 375 µg/kg) or saline was injected subcutaneously (sc) every 12 h; *n* = 5 WT and 5 C-α2^S/S^-KO mice injected with saline and *n* = 6 WT and 4 C-α2^S/S^-KO mice injected with ACTH. Bars represent means ± SE; *P < 0.05 vs. saline treatment (two-way ANOVA). Data from Ref. 116 regraphed and published with permission. *E*: in contrast to C-α2^S/S^-KO mice, mice with cardiovascular-specific KO of α2^S/S^ NKA (CV-α2^S/S^-KO) do not develop ACTH-induced hypertension. Protocol similar to that in *D*; *n* = 6 WT and 9 CV-α2^S/S^-KO mice injected with saline and *n* = 6 WT and 8 CV-α2^S/S^-KO mice injected with ACTH. *#*P* < 0.05 vs. the corresponding ACTH or saline basal SBP (two-way ANOVA). Data from Ref. 410 regraphed and published with permission.

These ouabain antagonism and immunoneutralization investigations are complemented by studies with Lingrel’s genetically engineered α2 NKA mice. The α2^R/R^ mice are unable either to develop or sustain hypertension that is normally induced in WT (α2^S/S^) mice by several common methods (Table 5). These include ACTH (Fig. 9, *A*–*C*) (109), Ang II + HS or deoxycorticosterone acetate (DOCA) + HS, and IP or ICV ouabain (references in Table 5). ACTH does, however, elevate BP more rapidly and to a greater extent in SWAP (α1^S/S^α2^R/R^) than in WT mice (Fig. 9*C*) (109). Also, knockout, by ∼90%, of the α2 NKA isoform in mouse cardiac muscle [C-α2^S/S^-KO; (116)] or both cardiac and VSM [CV-α2^S/S^-KO (410)] has little effect on basal BP, whereas CV-α2^S/S^-KO, but not C-α2^S/S^-KO, prevents ACTH-induced hypertension (Fig. 9, *D* and *E* and Table 5) (410). Furthermore, the hypertension in ACTH-injected α1^S/S^α2^R/R^ (109) as well as in ACTH-injected WT mice [Supplement Fig. S10 (109)] can be prevented by treatment with Digibind to immunoneutralize EO. Thus, EO and a VSM ouabain-sensitive NKA are required for the hypertension in mice, and either α2^S/S^ or α1^S/S^ will suffice. Reduction of smooth muscle (SM) α2^S/S^ NKA by ∼50–70% with a SM-selective α2-dominant negative construct (equivalent to inhibiting α2^S/S^ NKA by ∼50–70%), however, significantly elevates basal BP and augments Ang II-high salt hypertension (412). Conversely, transgenic mice with SM-specific overexpression (by >2-fold) of α2 NKA (SM-α2^S^-TG mice) are hypotensive (411, 412).

**Table 5.** Ouabain-resistant mutation of α2 NKA (α2^R/R^) or knockout of cardiovascular α2 NKA (CV-α2^S/S^-KO) in mice prevents or greatly attenuates hypertension; if, in the a2^R/R^ mice, α1 is made ouabain-sensitive (i.e., α1^S/S^α2^R/R^, or SWAP, mice), ACTH raises BP

Mouse Hypertension Model	Effect in Engineered Mice	References
IP ouabain	No BP increase in α2^R/R^	(414)
ICV ouabain	No BP increase in α2^R/R^	(417)
ACTH	No BP increase in α2^R/R^	(109, 461)
Ang II-salt	BP rise attenuated in α2^R/R^	(446)
ENaC upregulation + Na^+^-rich aCSF	No BP increase in α2^R/R^	(449)
ICV Na^+^-rich aCSF	BP rise attenuated/prevented in α2^R/R^	(417, 449)
Reno-vascular (2 kidney-1 clip)	BP elevation not sustained in α2^R/R^	(462)
ACTH	No BP increase in CV-α2^S/S^-KO	(410)
ACTH	Increases BP in α1^S/S^α2^R/R^ mice	(109)

Together, these data demonstrate that, in rodents, α2 NKA, its high-affinity ouabain-binding site, and its endogenous ligand, EO, all contribute to the pathogenesis of hypertension. It had been assumed that VSM α2^S/S^ NKA is primarily responsible for these effects, but Leenen and colleagues identified a novel slow neuromodulatory pathway in the hypothalamus that plays a crucial role in regulating sympathetic drive to the cardiovascular system. This brain pathway includes Ang II, aldosterone, epithelial Na^+^ channels (ENaC), EO, and α2 NKA. Interference, locally, with any step in this pathway (Fig. 8*D*; and see *The Hunt for the Holy Spirit*), including brain EO [e.g., with ICV Digibind or anti-ouabain antibodies (Table 4)], greatly attenuates or prevents many forms of experimental hypertension (463). It thus seems clear that both central and vascular α2 NKA and their ouabain-binding sites are critical in the pathogenesis of hypertension.

Despite these seemingly straightforward results related to hypertension, the α2^R/R^ mice pose a paradox with respect to their basal BP. Baseline data reveal that the mean BP (telemetry or direct arterial measurement) in untreated α2^R/R^ mice is ∼8 mmHg higher (*n* = 5 studies; range = 4.2–10.4 mmHg) than in WT mice (Table 3 in Ref. 170). In contrast, systolic BP (tail cuff) is ∼10 mmHg lower in pregnant α2^R/R^ dams than in pregnant WT dams (464). The α2 NKA ouabain-binding site and its endogenous ligand must, therefore, play a role in regulating vascular reactivity and BP in mice even under basal conditions. Pregnancy is a high-plasma volume state in which plasma EO is elevated in both rats (115) and humans (119) but BP is reduced relative to the nongravid state (465). Pregnant rats are remarkably resistant to the hypertensinogenic effects of EO and exogenous ouabain; this may, at least in part, be due to greatly reduced expression of NCX1.3, the vascular isoform subtype (115), but whether this also applies to humans has not yet been investigated.

#### MBG and hypertension.

Several reports suggest that MBG, too, is involved in the pathogenesis of hypertension (466, 467) but the evidence is much more fragmentary than for ouabain. First, like ouabain, MBG, isolated from the toad, *Bufo marinus*, can raise BP when infused into rats for 3–4 wk (468, 469). Second, elevated plasma MBG has been measured by immunoassay in humans with hypertension and hypertensive animals. In one report on human subjects, plasma MBG and an eOLC were both elevated in patients with primary aldosteronism, but only MBG was elevated in patients with essential hypertension (470), and see Table 3 regarding EO. Another group reported that immunoreactive MBG levels were much higher than eOLC levels in patients with primary aldosteronism than in patients with essential hypertension, but there were no normotensive controls in that study (471). A study on ACTH-induced hypertension in rats showed that plasma MBG, but not eOLC, was elevated (472). Another study from the same group found that MBG, but not eOLC was elevated in DS rats on an 8% NaCl diet for 4 wk (but contrast Fig. 7*D*) when systolic BP rose from 120 to 196 mmHg (sic!) and the rats showed signs of left ventricular hypertrophy. After 9–12 wk on the HS diet, however, when the rats went into HF, MBG fell to the control level while eOLC rose to about triple the control level (473). Finally, in spontaneously hypertensive rats (SHR), MBG was lower than it was in the control Wistar-Kyoto (WKY) rats, but eOLC was not measured (474). Thus, the link between MBG and hypertension appears more tenuous than the link to EO.

#### Preeclampsia.

The groups of Bagrov and Puschett detected elevated plasma MBG levels in pregnant, preeclamptic women and invoked a role for MBG in the pathogenesis of preeclampsia (475–477). Bagrov and colleagues also observed “dramatically” increased levels of a more polar eOLC in one study of patients with preeclampsia (475) but a smaller increase in a second study (477). Both groups employed salt-loading (an HS diet, alone, or DOCA + salt) to elevate BP in normal pregnant rats as a rodent model of preeclampsia; the studies showed that polyclonal or monoclonal antibodies to MBG lowered the BP in the salt-loaded, versus nonsalt-loaded rats (477–479). In normal pregnant rats, intraperitoneal (IP) injection of MBG daily for 2 wk elevated BP to the level observed in the pregnant DOCA-salt model (478). In the latter model, administration of the MBG analog, resibufogenin, which has an H- rather than OH- at C_5_, prevented hypertension and oxidative stress normally observed in this preeclampsia model, perhaps evidence of MBG-resibufogenin antagonism (480, 481).

Several investigators have suggested that an endogenous digitalis-like compound (eDLC), although not always distinguished from eOLC, might also play a role in preeclampsia (references in 482). This led to a small clinical trial of anti-digoxin Fab fragments for the treatment of preeclampsia. The Fab therapy failed to lower BP significantly in the patients with preeclampsia, but it did reduce maternal pulmonary edema and fetal intraventricular hemorrhage (483).

Elevated circulating levels of EO have been described in normal human and rodent pregnancies (226, 484, 485). The circulating levels were further increased among patients with preeclampsia (475) and this led to the impression that EO contributed to the elevated BP and associated outcomes. To test this concept, Rana and colleagues administered ouabain (20 µg/kg/day, ip) on gestation days 12–19 to pregnant rats with a uterine artery constriction (reduced uterine perfusion pressure, RUPP) model of preeclampsia. The addition of exogenous ouabain lowered mean BP from 126.3 ± 1.5 to 119.2 ± 2.0 mmHg and enhanced phosphorylation of placental heat-shock protein 27 (p-HSP27) without adversely affecting the pups (486). The authors interpret their result as an upstream step in the reduction of the placental vascular endothelial growth factor (VEGF) receptor that is upregulated in preeclampsia (487, 488). More critically, the results suggest that the elevated EO in patients with preeclampsia potentially is a compensatory/protective response that may reduce inflammation. This may explain why immunoneutralization of EO failed to lower BP in patients with preeclampsia.

Imbalances in angiogenic factors and inflammation have been implicated in the pathogenesis of preeclampsia (486). The aforementioned evidence that MBG and/or other eCTS may also be involved raises the possibility that these eCTS may mediate the inflammation and angiogenic factor imbalance.

#### Idiopathic pulmonary hypertension.

The preceding sections all concern systemic hypertension. In contrast, a recent “exploratory study” indicates that EO also is elevated in patients with idiopathic pulmonary arterial hypertension (489). In this group of five patients, plasma EO was negatively correlated with pulmonary artery pressure and total pulmonary artery resistance, but positively with cardiac index. The authors suggest that the elevated EO may be a compensatory response to increased right ventricular afterload.

Before moving on it should be mentioned that one group claimed that MBG inhibits rat aorta ouabain-resistant α1 (α1^R^) with ∼25-fold higher affinity, but ouabain-sensitive α3 (α3^S^) NKA with ∼50-fold lower affinity than does ouabain (490). The implication is that α1^R^ NKA is not MBG-resistant because MBG has a much higher affinity for rodent α1^R^ than α3^S^ NKA (490). This is surprising because the AA substitutions at positions 111 and 122 on the rodent α1^R^ NKA M1-M2 external loop interfere with both cardenolide and bufadienolide binding (55) (and see *CTS as an Animal Defense Mechanism*). Also, others have reported that MBG has a lower affinity for pig kidney α1^S^ than does ouabain (55, 358) and, while the affinity of ouabain for rat kidney α1^R^ is ∼60-fold lower than for pig kidney α1^S^, there was no measurable binding of MBG to the rat α1^R^ (55). In sum, the several apparent discrepancies in the MBG literature are worrisome and indicate the need for further investigation.

### Are NKA Ouabain-Binding Sites and EO Involved in the Manifestations of Heart Failure?

**Figure 10.**
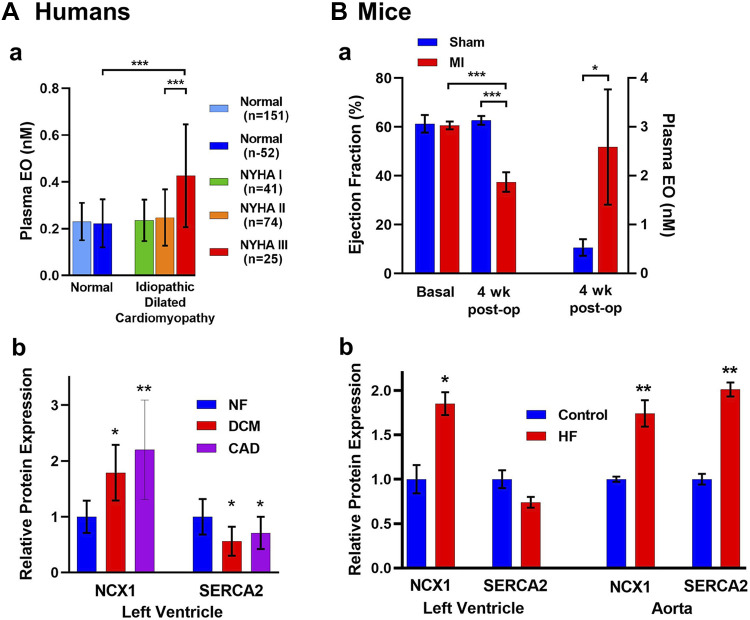
Association between plasma endogenous ouabain (EO), Ca^2+^ transporter expression, and heart failure (HF) in humans and mice. *Aa*: plasma EO in normal human subjects and patients with idiopathic dilated cardiomyopathy. Severity of HF is denoted by the New York Heart Association (NYHA) classification: I. No limitation of physical activity; II. slight limitation of physical activity; III. marked limitation of physical activity (modest activity causes fatigue, palpitations or dyspnea). Plasma EO measured with an ouabain-specific radioimmunoassay (RIA); results were confirmed with liquid chromatography-mass spectroscopy (LCMS) in four random patients. “*n*” = numbers of patients in each group. Data are shown as means ± SE; ***P < 0.001 (ANOVA). Data from Table 3 in Ref. 241. *b*: expression of Na^+^/Ca^2+^ exchanger = 1 (NCX1) and sarco-/endoplasmic reticulum Ca^2+^ ATPase-2 (SERCA2; likely SERCA2a, the predominant cardiac isoform) in the left ventricle (LV) of patients with non-failing (NF) hearts and those in HF. Patients with HF due to either dilated cardiomyopathy (DCM) or coronary artery disease (CAD) increased expression of NCX1 and reduced expression of SERCA2a in the LV. *P < 0.05; **P < 0.01 vs. NF (ANOVA). Data regraphed from Ref. 491 and used with permission. *Ba*: a left ventricular (LV) myocardial infarction (MI ≈ 45% of LV) was induced in C57Bl/6 mice (*n* = 5) by ligation of the left anterior descending coronary artery; controls (*n* = 5) were subjected to sham surgery. Four weeks after surgery, the LV ejection fraction, determined by echocardiography, had declined by >33% in the operated mice, indicative of HF, and plasma EO was significantly increased. *b*: NCX1 expression was increased and SERCA2 (likely SERCA 2a) was decreased in the LV of mice in HF, as in humans (*Ab*). Expression of SERCA2 (probably the 2 b isoform, which predominates in vascular smooth muscle) as well as NCX1 was increased in the aortae of the mice in HF (also see Fig. 6). *P < 0.05; **P < 0.01; ***P < 0.001 vs. sham (ANOVA). Data in *Ba* and *Bb* from Ref. 341 used with permission.

Plasma EO is elevated in HF in both humans (Fig. 10*Aa*) (92, 228, 241, 492) and mice (Fig. 10*Ba* and Table 6) (341). Even before the identification of EO, Shenkman and colleagues, using an anti-digoxin RIA, had reported that the level of a “digoxin-like immunoreactive substance” of adrenal origin (493) is increased in human HF (492). Furthermore, cardiomyocyte Ca^2+^ transporter protein expression is altered during HF in humans and animals. Notably, NCX1 and TRPC6 are usually upregulated, and SERCA2a, the predominant sarcoendoplasmic reticulum Ca^2+^ pump (SERCA) isoform in the heart (494), is usually downregulated in HF (495–498), albeit not always (499). Since prolonged treatment with low-dose ouabain increases NCX1 expression in rat cardiomyocytes (Fig. 4*F*; TRPC6 was not tested), it seems likely that, as in VSM (Fig. 4, *C*–*E*), the altered cardiomyocyte Ca^2+^ transporter expression in HF may be triggered by EO-activated PK signaling.

**Table 6.** Heart failure studies in which elevated plasma EO levels have been measured

Subjects with Heart Failure	Assay Method	Notes	References
Human HF	Ouabain ELISA (229)	Inverse correlation between EO and cardiac output	(92)
Human HF	Anti-ouabain RIA	EO confirmed by LC-MS; RIA/LC-MS correlation coefficient: r =0.89	(241)
Human HF	Anti-ouabain RIA	Negative correlation between EO and cardiac left ventricular ejection fraction	(228)
Mouse MI	Anti-ouabain RIA	See Fig. 10*Ba*	(341)

To determine whether the α2 NKA ouabain-binding site has a role in HF, experiments were performed on α2^R/R^ mice, SWAP mice, C-α2^S/S^-KO mice, and transgenic (TG) mice that overexpress either α1^R^ NKA or α2^R^ NKA in the heart (C-α1^R^TG and C-α2^R^TG mice, respectively) (500, 501). In the two TG models, the transgene was inserted into FVB/N background WT mice with an α1^R/R^α2^S/S^ genotype (500). HF was induced either by transaortic constriction (TAC) to elevate thoracic aorta BP and cause cardiac pressure overload (116, 415, 500) or by coronary artery ligation and consequent myocardial infarction (MI) (410). The results are summarized in Table 7 where the structural manifestations of HF include cardiac remodeling and hypertrophy, while cardiac “dysfunction” includes decreased cardiomyocyte shortening, reduced ejection fraction, and increases in left ventricular end-systolic and end-diastolic volumes. The data (Table 7) are consistent with the evidence that ouabain activates the generation of ROS, a mediator of the cardiac hypertrophy phenotype (103).

**Table 7.** Effects of genetic engineering of NKA on the manifestations of heart failure (cardiac hypertrophy and cardiac dysfunction) in mice

HF Method	Duration (Wk)	Genetic Model	Cardiac Pathology in Engineered/Treated Mice		References
			Hypertropy*	Dysfunction**	
Cardiac-specific decrease of α2^S^ expression:
TAC	(9 wk)	C-α2^S/S^-KO	Attenuated	Attenuated	(116)
TAC	(10 and 16 wk)	C-α2^R^-TG	Attenuated	Attenuated	(500)
MI	(8 wk)	C-α2^R^-TG	Attenuated	Attenuated	(501)
Cardiac-specific increase of α1^R^ expression:		
TAC	(12 and 16 wk)	C-α1^R^-TG	No attenuation	No attenuation	(500)
SWAP (α1^S/S^α2^R/R^) mice:		
TAC	(4 wk)	α1^S/S^α2^R/R^	Augmented	Augmented	(415)
TAC	(4 wk) Digibind (last 2 wk)	α1^S/S^α2^R/R^	No Augmentation	No augmentation	(415)

Some genetic alterations resulted in reduced cardiac ouabain-sensitive α2^S^ NKA expression: *1*) cardiac-specific knockout of cardiac α2 NKA (C-α2^S/S^-KO); *2*) cardiac-specific transgenic overexpression of ouabain-resistant α2^R^ NKA (C-α2^R^-TG), knockout of cardiac α2^S^ NKA (C-α2^S/S^-KO); and *3*) replacement of α2^S^ genes by α2^R^ genes (α1^R/R^α2^R/R^ mice). Cardiac-specific TG overexpression of ouabain-resistant α1^R^ NKA (C-α1^R^-TG) and reversal of ouabain sensitivity of α1 and α2 (α1^S/S^α2^R/R^ or “SWAP” mice) versus wild-type (α1^R/R^α2^S/S^ or WT mice) were also tested, as was treatment with Digibind to immunoneutralize EO).

*Hypertrophy refers to evidence of cardiac hypertrophy and remodeling such as heart weight/body weight ratios, myocyte cross-section diameters or areas, and/or fibrosis.

**Dysfunction refers to echocardiographic evidence of cardiac dysfunction such as impaired ventricular muscle fractional shortening, reduced cardiac ejection fraction, and altered left ventricular end diastolic and/or systolic volume.

The expression of α2^S^ NKA was greatly reduced specifically in cardiomyocytes in the two models, C-α2^S/S^-KO and C-α2^R^-TG mice. In the latter model, however, most of the α2^S^ NKA was replaced by a far greater number of α2^R^ NKA so that most α2 could still function in the presence of elevated EO. In all three studies on these two models, the structural and functional manifestations of HF were attenuated (Table 7; 116, 500, 501). This implies that the lack of α2^S^ and predominance of α1^R^ or α2^R^ NKA expression was responsible for the attenuation. One might then have expected a similarly attenuated response in α1^R/R^α2^R/R^ versus WT mice but, because the post-TAC observation period was very brief (only 4 wk), even the WT mice displayed negligible functional manifestation of HF (415). The latter study was also compromised by the small numbers of animals (*n* = 3–5) in some of the groups. Indeed, this issue, insufficient statistical power, was a problem in two of the other studies as well, e.g., see Fig. 6C in Ref. 116 and Fig. 5, C and D, in Ref. 500, which display type II statistical errors (also see discussion of this issue in Ref. 170).

Despite the short (4 wk) post-TAC period, SWAP (α1^S/S^α2^R/R^) mice, in which all the α1^R^ was replaced by α1^S^, exhibited accelerated manifestations of HF even in the absence of α2^S^ NKA. These effects were prevented by infusing the mice with Digibind during the last 2 wk of the post-TAC period [Table 7 (415)]. The inference is that the manifestations of TAC-induced HF were due to the interaction of EO with the cardiac α1^S^ NKA in this model. These data add to the evidence that ouabain-sensitive NKA is important for the development of HF; furthermore, they suggest that, in humans, with α1^S^ NKA, this isoform, too, might contribute to the pathogenesis of HF.

In contrast to the mice with markedly reduced α2^S^ NKA expression, including C-α2^R^-TG mice, overexpression of α1^R^ in the heart (C-α1^R^-TG mice) neither attenuated nor augmented the manifestations of HF (Table 7); i.e., the cardiac changes mimicked those in WT mice (500). This is not surprising because the cardiac genotype of the TG mice was identical to WT, i.e., α1^R/R^α2^S/S^, even though α1^R^ expression was increased and α2^S^ expression was somewhat reduced (500).

Based on the data in Tables 3, 4, 5, 6 and 7, it is difficult to escape the conclusion that NKA, its ouabain-binding site, and EO play key roles in the pathogenesis of hypertension and the cardiac structural and functional changes that occur in HF. The genetic engineering experiments surely allay any doubt about whether NKA and its ouabain-binding site are the relevant ouabain receptor (221). It is astonishing, therefore, how little attention has been paid to these findings in developing new strategies for therapy despite the prevalence and seriousness of these diseases.

### NKA Ouabain-Binding Sites and EO Contribute to Normal Physiology as Well as Pathophysiology

In addition to its pathophysiological actions in the cardiovascular system, EO and, thus, the ouabain-binding site, have been implicated in the pathophysiology of a number of other diseases. As discussed below, these include acute kidney injury (502) and autosomal dominant polycystic kidney disease (ADPKD) (503, 504), mania and bipolar disorders (505, 506), and cancer metastases (123). But how about normal physiology? After all, ouabain-sensitive NKA isoforms are present in virtually all cells in mammals [canine erythrocytes are an exception (507)]. The limited data available, cited in the preceding two sections, indicate that basal cardiac function appears to be unimpaired in α2^R/R^ and SWAP mice but the role of the α2 NKA ouabain-binding site in normal BP regulation was discussed. In another effort to determine whether eCTS influences normal cardiac function, Pierre and colleagues compared WT and α1^S^ (α1^S/S^α2^S/S^) mice (508). They found that ROS production was elevated and the hearts were modestly hypertrophied in the α1^S^ mice, inferring that eCTS does apparently modulate the normal heart. They did not, however, attempt to immunoneutralize the eCTS, e.g., with anti-digoxin Fab fragments or anti-ouabain antibodies, or block the effect with rostafuroxin, to confirm their inference.

Genetic engineering of ouabain sensitivity has been confined to the α1 and α2 isoforms and, for the most part, limited to studies on the CV system and skeletal muscle. There is, nevertheless, a wealth of evidence that the EO-NKA ouabain-binding site couple plays countless roles in normal physiology. These activities are modulated by EO-activated PK signaling and, most likely, also by EO/NKA influences on [Na^+^]_CYT_ and [Ca^2+^]_CYT_. Unfortunately, testing for EO involvement in physiological processes has therefore been largely limited to EO immunoneutralization studies with anti-ouabain antibodies or anti-digoxin Fab, or to treatment with low or, too often, high doses of ouabain or other CTS.^10^ In the following sections, we briefly summarize some examples from the enormous literature with an emphasis on recently published articles (especially in the past 5 years). Additional details and references are given in a recent review (8).

#### NKA-independent effects of CTS.

Before addressing these data on CTS-NKA interactions, we need to mention, briefly, articles on the NKA-independent effects of CTS. These are sporadic reports because there has not been a systematic analysis. Two recent examples illustrate some of the critical issues surrounding these studies. One such report describes the inhibition of atrial-specific K_2P_3.1 two-pore K^+^ channels by digitoxin and digoxin, which bind to AAs on the cytoplasmic side of the channel (509). A problem is that the IC_50_ for digitoxin inhibition is 7.4 µM, well into the lethal range (Supplemental Fig. S9). Another report describes the suppression of pyruvate kinase M2- (PKM2-) promoted transactivation of HIF-1α, which protects the liver from steatohepatitis (510). In this case, the effect was observed in mice in vivo with IP doses of 0.05–1.0 mg/kg, and 1–10 nM digoxin improved the redox status of human neutrophils treated with a ROS indicator (CM-H2DCFDA), but a reported digoxin concentration of 10 mM was used to demonstrate direct binding to PKM2.^11^ This raises the question of whether the in vivo effects might actually be mediated by CTS-NKA-mediated signaling and activation of a PK cascade. Furthermore, the intracellular sites are likely not accessible to ouabain because it is very hydrophilic; thus, this is probably not a general effect of CTS but only a possible effect of lipophilic CTS.

#### Cell viability, growth, and proliferation.

The discovery of the molecular mechanisms that underlie the growth-enhancing effects of low doses of CTS by Askari and Xie raised questions about the possible influence of low-circulating effects of EO on normal physiology. Indeed, subnanomolar concentrations of ouabain are associated with cell proliferation [(14); Supplemental Fig. S9*A*]. Numerous in vitro studies demonstrate that low concentrations of ouabain, e.g., <10 nM in rat retinal ganglion cells (511), and other CTS stimulate proliferation and improve viability of a variety of types of cells in culture. Further evidence for the role of EO/ouabain in cell growth and proliferation will be presented in *Pregnancy and fetal development* and *CTS and cancer*.

Commercial FBS and horse serum, which are routinely included in tissue culture media, contain EO (224), and lowering the culture medium EO content with anti-ouabain antibodies decreases the viability of a number of cell lines in culture, likely, in part, by curtailing EO-induced activation of ERK1/2 (224). Conversely, supplementation with low-dose ouabain may enhance growth; e.g., in experiments on chicken 12-day embryo bone tissue explants grown in nutrient media with 15% FBS (512). The bone explants exhibited greatly enhanced growth when 1 pM–100 pM ouabain, but not digoxin, was added to the medium; higher concentrations (10 nM–100 µM ouabain, in a dose-dependent manner, or 1 µM digoxin) inhibited bone growth. Indeed, very high concentrations of ouabain, e.g., 25 nM–2 µM in adult rat cardiomyocytes (513) or 1–10 µM in human neuroblastoma cells (514), can cause cell death [additional references in (224)].

#### Intercellular junctions and cell-cell communication.

CTS-activated PK cascades are intimately involved in modulating cell-cell interactions. Low concentrations of ouabain promote intercellular junction formation and intercellular signaling, but higher concentrations uncouple cells (8, 12, 515). A good example is the action of ouabain on Madin-Darby canine kidney (MDCK) cells described by Cereijido and Ponce (515, 516). Ouabain (10 nM) enhances the formation of “tight” “gap junctions” and, thus, intercellular communication, by increasing E-cadherin and β-catenin expression in the PM. These effects of ouabain are not observed in ouabain-resistant MDCK-R cells (517) with α1^R^ instead of the normal α1^S^ (518). The ouabain-promoted gap junction formation is apparently mediated by Src and ERK1/2 because junction formation is blocked by selective blockers of these PKs (518). Recently, the Cereijido and Ponce group reported that ouabain-promoted formation of tight junctions also is mediated by the small GTPase, RhoA, and Rho-associated PK (ROCK) signaling, and is prevented by inhibitors of RhoA activation and ROCK (519). Whether RhoA/ROCK lie downstream of the Src/ERK1/2 signaling pathway, or are in an independent pathway, is unknown. Like ouabain, low doses of digoxin and MBG also promote gap junction formation in MDCK cells (520). In addition to tight junctions, ouabain (10 nM) upregulates adherens junctions in MDCK-S cells, which suggests that ouabain has a physiological role in the modulation of cell contacts (518). Loss of this function might imply a role in some cancers.

#### The immune system and inflammation.

EO is a “stress hormone,” and the plasma EO level is elevated under many stressful conditions including cardiac surgery, renal failure, and critical illness (228, 521, 522). This hormone modulates both the immune system and inflammation via PK signaling pathways (13). As mentioned above, ouabain stimulates ROS generation (103). In turn, this oxidative stress activates the immune system; it can trigger the secretion of proinflammatory cytokines such as TNF-α, IL-1β, and IL-6 (523) and induce an inflammatory response consistent with the following sequence (8):

EO → ↑ROS → immune system activation → ↑proinflammatory cytokines → inflammation.

Importantly, EO can trigger pathological inflammatory responses that may involve neutrophils (524) and activation of the nuclear transcription factor, NF-κB (525), a “master regulator” of inflammation (526, 527) and, while NF-κB can modulate ROS, ROS can stimulate or inhibit NF-κB signaling depending upon context (528, 529).

Low-dose EO can also exert a crucial beneficial effect by suppressing inflammation, as demonstrated by several groups using in vitro and, especially, in vivo models. For example, ouabain, injected into mice IP, downregulates TNF-α, IL-1β, and IL-6 and thereby protects the mice against lipopolysaccharide-induced acute lung injury and lethal endotoxemia (523, 530). In another study, ouabain and α2 NKA silencing both protected cultured mouse glial cells from lipopolysaccharide-induced, NF-κB-mediated inflammatory responses (531), thereby confirming the key role of NKA in inflammation. (Regrettably, they did not compare cells from α2^R^ and α2^S^ mice to determine if the crucial factor was the ouabain binding site rather than α2 NKA itself.) IP ouabain also attenuates ovalbumin-induced acute airway inflammation and subsequent airway remodeling in a model of allergic asthma (532, 533). Bufalin and MBG (534–536), and digoxin (510, 537), too, reduce proinflammatory cytokines and exert an anti-inflammatory effect when administered IP in various mouse models including MI. The contrasting effects of CTS are apparent also in the brain as summarized elsewhere (538). The neuroprotective effect of ouabain is mediated by the NKA α2 isoform (539) and several neuroprotective CTS reduce proinflammatory cytokine production and inhibit NF-κB and p38 MAPK pathways. Despite these numerous reports, a common blind spot is revealed in a recent review of T-cell trafficking in cardiac and vascular inflammation (540). The article discusses the need for “better understanding of the central players” that modulate inflammation and may induce both the beneficial and harmful consequences of immune system activation but there is no mention of EO or other eCTS.

#### Antiviral actions of CTS.

Ouabain and other CTS interfere with viral entry and reproduction. The Zika virus is associated with neurological disorders and, in the fetus, leads to growth restriction and microcephaly. Both ouabain and digoxin, via their interaction with NKA, reduce Zika infection, lower the viral load, and protect the fetus from infection (541). Subsequent work has indicated that nanomolar concentrations of ouabain interfere with the replication step of the virus once it has entered the cell (542). In addition, therapeutic concentrations of ouabain and digoxin have antiviral activity against the SARS-CoV-2 virus (543). Screening and modeling studies again support the view that the mechanism is the immediate postentry interference with the replication process before the virus gains full entry to the cytoplasm (544, 545). This same effect apparently extends to influenza as well as other viruses including Japanese encephalitis and Chikungunya viruses (546–548). A precytoplasmic effect of the CTS is supported by the view that ouabain, which is very hydrophilic (8), does not ordinarily (biosynthetic sites excepted) achieve high nanomolar concentrations in the cytoplasm. In contrast, the equally efficacious, but more hydrophobic, CTS such as digoxin, bufalin, and MBG may be subject to significant tissue accumulation.

#### Kidney function.

Aperia and colleagues observed that subnanomolar to low nanomolar ouabain induces low-frequency Ca^2+^ oscillations in primary cultured rat renal proximal tubule cells and immortalized COS-7 monkey kidney cells. The oscillations depend upon interaction between the PM α1 NKA and endoplasmic reticulum inositol trisphosphate receptors (InsP_3_R) (10, 378, 549). The Ca^2+^ oscillations, by activating NF-κB and by phosphorylating and activating CaMKIIγ, protect the kidney cells from apoptosis (10). Slightly higher ouabain concentrations, however, downregulate nephrin, a crucial component of the glomerular filtration barrier, in rat renal podocytes and in rat kidneys in vivo; in the rat, this is manifested by proteinuria and reduced creatinine clearance (242, 550). The ouabain receptor antagonist, rostafuroxin, attenuates the podocyte nephrin downregulation in vitro and glomerular dysfunction, measured in vivo by a decline in creatinine clearance (550, 551).

The kidney proximal tubule apical membrane NHE3, which imports Na^+^ from the tubule lumen into the tubule epithelial cells in exchange for protons, is modulated by dietary salt and EO. Renal NHE3 expression is decreased in salt-loaded rats (552) and, in normal men, salt loading causes plasma EO to rise (227). In cultured LLC-PK1 cells, a pig kidney proximal tubule cell line, 50–100 nM ouabain treatment for 24 h (but see Footnote no. 10 and Supplemental Fig. S9) markedly downregulates NHE3 expression by promoting NHE3 trafficking from the apical membrane to early endosomes (268, 553). In vivo, the resultant reduction of Na^+^ transport from the tubule lumen into the proximal tubule cells, where 60–70% of intralumenal Na^+^ is reabsorbed, would be expected to promote natriuresis at least transiently.

SWAP mice, with α1^S^ but α2^R^ NKA, exhibit enhanced natriuresis to a saline load, versus WT and α2^R/R^ mice; this enhancement is blocked by infusion of anti-ouabain antibodies (554). Because high dietary salt elevates plasma EO in normal men (227), the SWAP mouse data suggest that EO may contribute to salt-induced natriuresis in humans because human renal epithelial cell NKA is largely (but not quite all) α1^S^. Infusion of anti-ouabain antibodies into normal rats for 4 wk, however, causes a significant, albeit transient, reduction in natriuresis on day 2 (223) even though rat kidney epithelial α1 NKA is α1^R^. In this situation, the implication is that, despite very low expression in the kidneys, α2^S^ or α3^S^ also plays a role in the natriuresis.

Notwithstanding these data and the early ideas that the eOLC might be a natriuretic agent (83) (see *The Idea of a Circulating Ouabain-Like Compound: Possible Clinical Implications*), the role of ouabain in natriuresis is remarkably complex and is dependent, in part, on dose and duration of treatment. For example, while it is obvious that acute inhibition of renal tubule basolateral membrane NKA will reduce Na^+^ reabsorption, we now know that low subnanomolar doses can stimulate the NKA and enhance Na^+^ retention. Furthermore, long-term ouabain treatment can also promote Na^+^ retention by inhibiting dopamine-mediated downregulation of renal NKA. Normally, high dietary salt increases local dopamine generation in the kidneys (555). This dopamine stimulates the dopamine receptor D_1_R subtype, thereby activating PKCζ, which phosphorylates renal tubular NKA and promotes its endocytosis (555–557). Local dopamine also triggers a decrease in tubular NHE3 protein by promoting endocytosis (558). Thus, by reducing the expression of both Na^+^ transporters, apical NHE3 and basolateral NKA, dopamine reduces transepithelial Na^+^ reabsorption in the kidneys and thereby promotes natriuresis. As we have seen, however, high dietary salt and NaCl retention also elevates circulating EO acutely. While ouabain downregulates NHE3 expression (268, 553), ouabain also downregulates D_1_R expression; this, in turn, will reduce the dopamine-activated downregulation of both NHE3 and NKA and promote Na^+^ retention (412, 433, 559). Thus, prolonged EO elevation (i.e., up to 1 nM; Fig. 2*C*) may be antinatriuretic.

In chickens, low-dose ouabain infusion stimulated Na^+^ reabsorption by 40% while higher doses were natriuretic (284). Low-dose ouabain infusion slightly reduced fractional excretion of Na^+^ (Fe_Na_) in dogs in HF, but increased Fe_Na_ at a higher (“digitalizing”) dose, whereas both low and higher doses increased Fe_Na_ in normal dogs (560). Several clinical studies that have examined the relationship between the variations in plasma EO and renal function during and following salt loading also have suggested that EO within the measured range of 100–500 pM has a salt-retaining effect (561–564). Furthermore, in the general population, elevation of EO appeared to counter the depressor action of Na^+^ depletion (565). As might be anticipated from these observations, plasma EO, like aldosterone, is elevated in normal humans in response to salt depletion (227). This antinatriuretic mechanism may have evolved in parallel with aldosterone as a protective mechanism to conserve body Na^+^ because most mammals, including early humans, ate salt-deficient diets. Indeed, aldosterone and EO are coelevated in approximately one-third of patients with hypertension, and the BP in those patients is highly salt-sensitive (243).

With the aforementioned data on ouabain/EO and renal function in mind, we should consider how they relate to the well-documented but imprecisely understood relationship between salt and the kidneys in the pathogenesis of hypertension (436). These numerous, sometimes contravening actions of ouabain (NKA-mediated Na^+^ transport stimulation/inhibition, activation of Ca^2+^ and PK cascade signaling, and retrieval and recycling of NKA and NHE3) indicate the difficulty in sorting out the precise way that the kidneys contribute to hypertension. It seems likely that the EO level and the duration that level is maintained may affect the answer.

Autosomal dominant polycystic kidney disease (ADPKD) is a lethal genetic disorder in which large cysts are formed in the kidneys as a result of epithelial cell proliferation, thereby destroying normal renal tissue. The NKA in the epithelium that surrounds the cysts is mislocalized to the apical membrane of the epithelial cells versus the basolateral localization in normal renal tissue. This aberrant distribution may be due to the formation of α1β2 NKA protomers found in ADPKD cysts, rather than the α1β1 protomers present in normal renal tissue (566). Importantly, low nanomolar ouabain stimulates the mislocalized NKA to promote Na^+^, Cl^−^, and water secretion into the lumen of the cysts (504, 567). These studies have all been performed with low concentrations of exogenous ouabain in vitro. Ideally, one would like to see these ideas verified in vivo in an ADPKD animal model (568) in which cyst volume might be manipulated by immunoneutralizing EO or blocking its action with rostafuroxin, and by infusing low-dose ouabain or stimulating EO production with a precursor (105).

A number of investigations indicate that eCTS contributes to renal injury and that the linkage may, in part, be genetic. The group of Bagrov reported that plasma MBG, but not EO, was elevated in patients with chronic kidney disease, and that a similar result was obtained in partially nephrectomized, uremic rats (569). Postoperative acute kidney injury (AKI) is observed in ∼18% of cardiac surgery patients and portends a poor prognosis as it often leads to renal failure and death; a major contributor may be perioperative renal ischemia/hypoxia (570, 571). In clinical studies on three patient cohorts, Manunta and colleagues found that elevated preoperative plasma EO in patients undergoing cardiac surgery was a predictor of postoperative AKI (242, 502). Manunta’s group then showed that, in uninephrectomized rats, ischemia and reperfusion of the residual kidney caused EO to decrease in the plasma but increase in the ischemic kidney. Evidence of renal damage and reduced glomerular filtration rate (GFR) were observed at 24 h and had progressed by 72–120 h (551). The evidence of renal damage and reduced GFR were more severe when ouabain-hypertensive rats (30 µg ouabain SC/kg/day × 8 wk) were similarly subjected to ischemia and reperfusion. In both the “normal” and the ouabain-hypertensive rats, the post ischemia/reperfusion renal damage and reduced GFR were greatly attenuated if the rats were treated with oral rostafuroxin for 4 wk before the ischemia-reperfusion test (551). Recently, the same group suggested that missense variants in lanosterol synthase, which catalyzes the synthesis of cholesterol, an EO precursor, may predispose cardiac surgery patients to AKI (572). In contrast to these findings, however, Aperia’s group reported that in vivo treatment with ouabain (15 µg SC/kg/day × 4 mo) reduces renal cell apoptosis and podocyte loss in a rat model of human chronic kidney disease with proteinuria, but functional improvement was not mentioned (573).

#### Male reproductive physiology.

Following an early report that an eDLC is present in human seminal fluid (574), numerous articles provided evidence for a link between EO and spermatogenesis. Pretreatment of murine Leydig cells, located between the seminiferous tubules, with low-nanomolar ouabain activates PK signaling and augments progesterone secretion in response to luteinizing hormone stimulation (575). Likewise, low-nanomolar ouabain enhances steroidogenesis in rat testicular Sertoli cells, apparently mediated by α4 NKA (576, 577) in contrast to the α3-mediated action of EO in Leydig cells (575). Ouabain has also been implicated in the capacitation (penultimate step in maturation) of bull sperm, but only high (micromolar) concentrations were tested (578). The α4 NKA has also been implicated in sperm motility, although its high-affinity ouabain-binding site does not appear to be required for male mouse fertility (579).

#### Pregnancy and fetal development.

**Figure 11.**
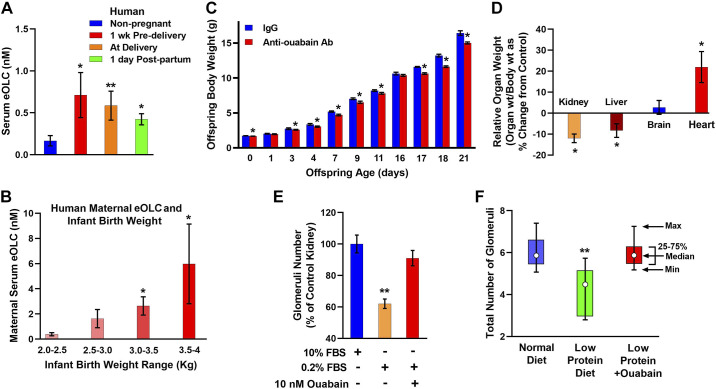
*A* and *B*: endogenous ouabain is elevated in pregnancy and plays a role in human fetal development. *A*: serum endogenous ouabain-like compound (eOLC) was measured in nonpregnant women and in women 1 wk before delivery, at the time of delivery, and 1 day after delivery. eOLC, which was measured by ELISA with selective anti-ouabain antibodies, was elevated during pregnancy and declined at the time of delivery and in the early postpartum period. *P < 0.05; **P < 0.01 vs. eOLC in nonpregnant women; *n* = 11–19 per group. *B*: neonatal birth weight correlates with maternal serum eOLC collected just before delivery: eOLC is lower in mothers of small-for-gestational-age neonates (2.0–2.5 kg) than in mothers of normal-for-gestational-age neonates (3.0–3.5 and 3.5–4.0 kg). *P < 0.05, *n* = 10–30/group. Data in *A* and *B* from Ref. 119 regraphed and used with permission. *C–F*: decreasing endogenous ouabain (EO) in rats lowers neonatal birth weight and impairs organ development. *C*: in rats, treatment of dams with anti-ouabain antibodies (Ab, 10 mg/kg/day) on days 9–18 of pregnancy leads to reduced neonatal birth weight and slower gain of weight by the pups for at least the first 21 postnatal days. *P < 0.05 vs. offspring of dams injected with IgG (10 mg/kg/day); *n* = 22–35. *D*: offspring from anti-ouabain Ab-treated and IgG-treated dams were euthanized at age 17 days. The relative weights (organ wt/body wt) of the kidneys and livers of pups from anti-ouabain Ab-treated dams were less, and heart weights were greater than those from the offspring of control (IgG-treated) dams; *P < 0.05; *n* = 9–12. Data in *C* and *D* from Ref. 119 were regraphed and used with permission. *E*: development of rat kidneys is impaired when they are cultured in fetal bovine serum-deprived media (0.2% FBS); adding ouabain to the media rescues the kidneys. [Note: FBS contains EO (224).] Embryonic day 14 (E14) explanted kidneys were cultured for 72 h in media containing 10% FBS; in some cultures, during the last 24 h, the medium was replaced with one containing only 0.2% FBS, either without or with 10 nM ouabain. Serum deprivation reduced the number of glomeruli in the kidneys by about 40%, but not if that medium was supplemented with 10 nM ouabain. **P < 0.01; *n* = 9–15. *F*: rats fed a low-protein diet (9% vs. normal 18% protein) throughout pregnancy gave birth to pups with abnormally low numbers of renal glomeruli; this loss was prevented by infusing ouabain into the dams (15 µg/kg/day via subcutaneous osmotic minipump) during the pregnancy. **P < 0.01; *n* = 8 per group. Data in *E* and *F* from Ref. 9 regraphed and used with permission.

We already alluded to the elevated plasma EO in normal pregnancy (*The NKA Ouabain-Binding Site-EO-BP Connection*). Figure 11, *A* and *B,* shows EO data from nonpregnant and pregnant humans measured with ouabain-selective antibodies. The EO level is elevated before delivery and declines at the time of delivery and in the early postpartum period (Fig. 11*A*). In humans, low birth weight is associated with low maternal circulating EO (Fig. 11*B*) (119) and, in the neonate, a reduced number of kidney glomeruli (580, 581) and greatly increased risk for hypertension and kidney disease in later life (582, 583).

Maternal malnutrition is an important cause of low birth weight (583). Feeding pregnant rats a low-protein diet (9, 584) or immunoneutralizing EO during the second half of pregnancy (119, 223) leads to low birth weight and cardiac enlargement, and impairs liver and kidney development (Fig. 11, *C* and *D*). Also, serum-deprived cultured fetal rat kidney explants exhibit retarded nephron formation, increased apoptosis, and a reduction in the total number of glomeruli. These effects are prevented by supplementing the serum-deprived medium with low nanomolar concentrations of ouabain (Fig. 11, *E* and *F*) that induce Ca^2+^ waves and activate NF-κB (9), presumably because the exogenous ouabain can replace the EO in the missing fetal bovine serum (224). As “proof of principle,” infusion of low-dose ouabain into protein-deprived, malnourished rat dams throughout pregnancy can rescue renal development (Fig. 11*F*) (9) and prevent hypertension in the adult offspring (585). Together, these results support the view that EO is a crucial growth factor during fetal development and has beneficial consequences for cardiorenal function later in life.

What is the significance of the elevated plasma EO and the resistance to the pressor effect of EO during pregnancy? One possibility is that high maternal EO may be needed so that enough can cross the placental barrier to provide the fetus with sufficient EO to support normal development. The maternal ouabain resistance may then be necessary to minimize ouabain’s vasoconstrictor and BP-elevating actions and maximize blood flow, especially to the placenta, as well as to avoid promoting uterine contraction during gestation.

#### Skeletal muscle and the modulation of exercise tolerance.

The α2^S^ NKA is by far the predominant isoform in skeletal muscle, and mice with skeletal muscle-specific KO of α2 NKA (sk-α2^S^-KO), although expressing markedly upregulated α1^R^ NKA, have a greatly reduced ability to perform exercise on a treadmill (409). Furthermore, α2^R/R^ mice demonstrate that the α2 ouabain-binding site and its endogenous ligand play an important role in exercise tolerance. Resting isolated skeletal (extensor digitorum longus, EDL) muscle NKA from α2^R/R^ mice exports less ^86^Rb (K^+^ mimic) than does NKA from WT mouse EDL. After 30 s of isotonic contraction, however, the α2^R/R^ EDL NKA transports much more ^86^Rb than does the WT EDL NKA. These findings in α2^R/R^ mouse EDL are mimicked by infusion of Digibind into WT mice just before isolation of the EDL (Supplemental Fig. S11*A*, *a* and *b*), implying that circulating EO and its interaction with the α2 NKA ouabain-binding site are responsible for the observations in WT EDL (586). The α2^R/R^ mice are also able to run faster and sustain moderate-intensity exercise (treadmill running) longer than WT mice (Supplement Fig. S11*B*) (586). This suggests that exercise tolerance, too, can be dynamically regulated by circulating EO and the α2 NKA ouabain-binding site, perhaps because of their influence on the rate of NKA-mediated transport.

Skeletal muscle disuse atrophy and dysfunction is often characterized by early depolarization. Pretreatment of rats for 4 days with IP ouabain was found to attenuate the depolarization in immobilized rat soleus muscle and to increase phosphorylation of AMPK (AMP-activated PK) and upregulate the mRNAs of IL-6 and its receptor (587). These data raise the possibility that the NKA might be a therapeutic target for attenuating muscle disuse atrophy.

#### Vascular physiology.

We discussed the evidence from α2^R/R^ mice that the α2 NKA ouabain-binding site and EO participate in the regulation of vascular tone and BP (*The NKA Ouabain-Binding Site-EO-BP Connection*). The PE dose-isometric force curves of WT and α2^R/R^ mouse aortae are, however, not significantly different (414). In contrast, PE-induced constriction of aortic rings from 4 wk anti-ouabain antibody-treated rats is significantly reduced, whereas vasodilation to atrial natriuretic peptide (ANP) is enhanced (223). In neither case were smaller arteries tested. Nevertheless, the implication is that even in the normal rat, the α2 NKA ouabain-binding site and EO help modulate vascular reactivity, and contractility may be reduced if the EO is removed for a prolonged period.

As emphasized in a recent review (588), in addition to helping regulate intracellular Ca^2+^ by influencing the Na^+^ gradient and NCX, EO also helps regulate the Ca^2+^ sensitivity of the VSM contractile apparatus. It does so by promoting the phosphorylation of cSrc which, in turn, promotes the phosphorylation of myosin phosphatase target subunit 1 (MYPT1); this inhibits myosin light-chain phosphatase (589). The resultant increase in phosphorylated myosin light chain results in Ca^2+^ sensitization.

The EO-vascular interaction is further complicated by the fact that ouabain also modulates endothelial function and the effects of ouabain on VSM and endothelial cells (ECs) can induce opposing actions. For example, while ouabain acutely promotes arterial myocyte contraction and vasoconstriction by augmenting myocyte Ca^2+^ signaling, it also enhances Ca^2+^ signaling in ECs and thereby promotes nitric oxide (NO) synthesis and vasodilation (590). Supplemental Fig. S4*C* shows that 0.1 nM ouabain acutely increases myogenic constriction of an endothelium-denuded small artery, whereas this dose was ineffective in the intact artery (not shown; see Ref. 61). Prolonged ouabain-induced activation of Src, however, increases ROS generation and downregulates NO production (591).

#### The brain.

Ouabain-activated signaling in the brain stimulates neurite outgrowth and influences learning and memory. For example, incubation of primary cultured rat cerebrocortical neurons with 1 µM ouabain for 6 h triggers slow Ca^2+^ oscillations, stimulates MAP kinases (e.g., ERK1/2) and CaM kinases, and initiates a transcription program dependent on CREB- and CRE-mediated gene expression (267). Dendrite growth (length and number) increased after 48 h of incubation with 0.1–1 µM ouabain and depended on Ca^2+^ influx as well as the MAP kinases and CaM kinases.

Bilateral infusion of a single low dose of ouabain (10 nM × 2 µL) directly into the hippocampus of rats activates NF-κB at 1–10 h, nuclear transcription factors CREB and BDNF by 10 h, and Wnt/β-catenin by 24 h (592). CA1 neurons exhibited increased dendritic branching by 7 days after ouabain infusion; after 14 days, CA1 neurons displayed increased neurite length and branching, and dentate gyrus neurons also exhibited increased branching. Spatial memory, tested in a Morris Water Maze, was significantly better in the ouabain-treated rats (learning phase, days 7–13 postinfusion; reference memory tests, days 14–15; working memory test, days 16–18). The EO-NKA ouabain-binding site couple therefore appears to play a role in synapse formation and learning and memory.

Bipolar disorders (BD) are a group of chronic and severe psychiatric illnesses characterized by mood fluctuations between mania, euthymia, and depression (593). Abnormally low-circulating levels of ouabain-like and digoxin-like eCTS together with the absence of normal cyclic variations in their circulating levels appear to be uniquely linked with BD (506, 594–596). In turn, this implies some abnormality in adrenocortical function specific to CTS biosynthesis because the circulating levels of aldosterone and cortisol in BD are not impacted dramatically (597, 598). It follows from the relationships shown in Fig. 2*B* that the manic and depressive phases might be determined by very slow oscillations in NKA activity driven by corresponding variations in brain eCTS concentrations. ICV infusion of anti-ouabain antibodies has been used in rodent models to help elucidate mechanisms underlying both depression and mania.

The manic phase of BD is often modeled by the administration of d-amphetamine, a norepinephrine/dopamine transporter inhibitor and dopamine releaser, to rats or mice (506). It has been noted, however, that aside from the increased lithium-inhibitable locomotor activity, this model does not reproduce some exploration-related features of bipolar mania (599) or the depressive phase. IP administration of d-amphetamine to mice (505) markedly increased locomotor activity and oxidative stress (indicated by altered levels of antioxidant enzymes such as superoxide dismutase) and tripled the brain eCTS level (measured by ELISA). Simultaneous ICV infusion of anti-ouabain antibodies, which should immunoneutralize EO in the brain, prevented the d-amphetamine-induced changes in brain antioxidant enzyme levels and the hyperactivity (505). Also, dextromethorphan, an abused cough suppressant that can cause mania in humans (600–602), induced hyperlocomotion in mice that was significantly attenuated by rostafuroxin (603).

A complement to these studies is the widely reproduced early observation of El-Mallakh that ICV administration of ouabain induced “manic-like” behavior in rats. This behavior can be detected as a marked increase in open field activity that persists for more than a week after a single central dose of ouabain (604–606). Common therapies for BD, lithium carbonate (the “gold standard”), and valproic acid (593), prevented the ICV ouabain-induced increase in open field activity (604, 607). Indeed, the ouabain model may be uniquely relevant to BD. Unlike any other model, it can reproduce the mood cycling aspect of BD, i.e., mania- and depression-like signs separated by a euthymia-like period (608). The primary event that triggers behavioral change is almost certainly the interaction of the additional ouabain with NKA in the brain. This model raises the possibility that abnormal up- and downregulation of local EO biosynthesis in critical brain regions might drive the BD phenotype. It seems remarkable that, even in this highly reproduced model, there are no data regarding the disposition of ouabain in the brain: What concentrations are achieved and, especially in those regions thought to be relevant to BD, how long do they linger? Which NKA α-isoforms are relevant? And, does ouabain interfere with the synthesis/levels of EO or other brain eCTS?

The “Flinders Sensitive Line” (FSL), a genetically depressed line of rats characterized by behavioral supersensitivity to diisopropyl fluorophosphate, a cholinesterase inhibitor, is often employed as a model of human depression (609). FSL rats display reduced mobility, exploration, climbing and swimming behaviors, and increased periods of immobility; sustained ICV infusion of anti-ouabain antibodies attenuates these behavioral changes (610). The studies on both the manic and the depressive animal models therefore strongly implicate the EO-NKA ouabain-binding site couple in the abnormal behavioral manifestations of BD (506).

In an uncontrolled (i.e., without placebo) preliminary study, six patients hospitalized with recurrent bipolar depression exhibited transient reduction of symptoms after a short period of Digibind therapy (Zilberstein et al. cited in Ref. 506). As a proof of concept, this result warrants a double-blind follow-up trial that also includes manic patients and is performed under rigorous conditions where all the relevant parameters (including the protein and excipients in Digibind) are properly controlled.

If the NKA and eCTS are involved in BD, genetic engineering, and monogenic diseases of NKA isoforms may support or deny the roles of eCTS and NKAs in behavior and bipolar disorders. To this end, the groups of Ikeda, Kirshenbaum, and Lingrel examined how the global deletion of one α2 or one α3 NKA allele influences behavior in mice. The α2 haploinsufficient mice had reduced locomotor activity and impaired spatial learning (Morris water maze) (38, 611); the mice also displayed an increase in fear and anxiety-related behavior (611, 612), possible evidence of depressive-type behavior. In contrast, α3 haploinsufficient mice exhibited increased locomotor activity and an enhanced locomotor response to methamphetamine and increased interest in exploring a novel environment, perhaps evidence of manic-type behavior; but they also displayed defective spatial learning and memory (38, 611). When chronically stressed, however, the α3 haploinsufficient mice exhibited 33% reduced NKA activity, despair-like behavior (tail suspension test), anhedonia (sucrose preference test), and sociability deficits (social interaction test) (613). In both haploinsufficient models, of course, the reduced brain-wide isoform expression may be equivalent to inhibiting a substantial fraction of that isoform with an eCTS. Overall, the results from these models prove a role for NKA and thus abnormal ion transport in mood and behavior. These models produce brain-wide deficits in NKA, however, and therefore might not fully mimic the impact of regional variations in eCTS concentrations in the brain.

Familial hemiplegic migraine type 2/sporadic hemiplegic migraine and rapid-onset dystonia parkinsonism/alternating hemiplegia of childhood are rare autosomal dominant monogenic neurological diseases involving, respectively, various heterologous mutations of α2 and α3 NKA (614–621). Only small fractions of patients with an α2 mutation and hemiplegic migraine or patients with an α3 mutation and rapid-onset dystonia parkinsonism reportedly exhibited behavioral manifestations such as depression or obsessive-compulsive disorder (617). One concern, however, is that behavioral problems may have been overlooked in many cases because of the focus on the overwhelming neurological issues. Indeed, a focused examination of psychiatric disorders in three families of patients with rapid-onset dystonia parkinsonism indicated that 50% of 26 patients suffered from depression or BD versus 22% in the 27 mutation-negative participants (622).

Another way to address this issue is to study the behavior of mice genetically engineered to express α2 and α3 NKA mutations. To this end, Clapcote and Kirshenbaum generated a mouse (“*Myshkin*” or “*Myk*”) with an α3 NKA I810N loss-of-function mutation (623) located in transmembrane α-helix M6. Most α3 mutations in cases of alternating hemiplegia of childhood are clustered in or near M6, including three involving isoleucine-810 (624). Homozygous *Myshkin* mice are embryonic-lethal, whereas heterozygotes (α3*^Myk^*^/+^), with 42% reduced total brain NKA activity, exhibit spontaneous epileptic seizures in vivo and neuronal hyperexcitability in hippocampal brain slices in vitro (623). Of note, a large fraction of patients with α2 or α3 NKA mutations also exhibit seizure activity (615, 617, 621). The seizures and hyperexcitiability, or “mania-like behavior,” in α3*^Myk^*^/+^ mice could be prevented by insertion of WT α3 NKA transgenes to increase expression of normal α3 (623).

In addition to mood-related animal models, numerous mutations have been described in human NKA α1 and α3 subunits as summarized elsewhere (625, 626). These mutations lead to disorders that include epilepsy, Charcot-Marie-Tooth disease type 2, Angelman’s syndrome, rapid dystonia parkinsonism, alternating hemiplegia of childhood, BD, complex spastic paraplegia, learning impairment, developmental delays, and for mutations in FXYD2, isolated dominant hypomagnesemia. In the adrenal glands, AA substitution at four sites in α1 as well as several deletion mutations lead to hyperaldosteronism (625). It is not known if these mutations impact the biosynthesis of aldosterone in the brain. The consequences of some changes in the α1 and α3 subunits seem obscure in terms of mechanism. Some lead to impaired NKA activity and promote elevated levels of intracellular Ca^2+^, a known stimulus for aldosterone synthesis. Other changes seem minimally related or even unrelated to NKA activity (625) and hence imply abnormal signaling and/or interactions with other intracellular partners.

#### Ménière’s disease and migraine.

A few authors have suggested that there is a link between migraine and Ménière’s disease (627–630); others have focused on altered cation transport mechanisms that may be common to migraine, Ménière’s, epilepsy, and affective disorders (629, 631–634). Ménière’s disease (idiopathic endolymphatic hydrops) is a disorder associated with elevated endolymph volume and pressure in the inner ear. It is manifested by vertigo, often preceded by hearing loss and tinnitus. More than half of patients with Ménière also suffer from migraines (627, 635), i.e., moderate-to-severe recurrent, unilateral, throbbing, pulsating headaches mediated by the trigemino-vascular pathway. The frequent co-occurrence of migraine and Ménière’s has raised the possibility that they are related disorders and that they may be linked by altered ionic regulation (629) possibly involving EO (633, 634, 636). One group, however, reported lower median plasma EO in patients with Ménière than in controls in one study (636), but higher levels in another study (634), with substantial overlap between the ranges of EO in the control and patients with Ménière. The possibility of local alteration of EO in the brain was not mentioned.

The frequent association between migraine and epilepsy may relate to a common pathogenetic mechanism as neuronal hyperexcitability is a risk factor for both (631, 633). Harrington and colleagues reported that the CSF, but not plasma, [Na^+^] was higher in migraineurs than in controls, and that it increased during the ictal phase, whereas CSF [K^+^], [Ca^2+^], and [Mg^2+^] were unchanged (637). They later found that CSF EO in migraineurs was lower in the ictal phase than in the interictal phase; plasma EO did not change between phases but was higher than in control subjects (633). In a rat study, they showed that, like IP nitroglycerin, immunoneutralization of CSF EO with IP or ICV DigiFab,^12^ or ICV infusion of Na^+^- rich aCSF, lowered the aversive mechanical threshold, evidence of increased neural excitability, and increased trigeminal nucleus cFos expression, indicative of neural activation. The nitroglycerin-triggered elevation of CSF [Na^+^] and sensitization to mechanical stimulation was prevented by either IP or ICV administration of ouabain (633). The authors suggest that altered choroid plexus NKA activity and an elevated CSF [Na^+^] concentration may be a triggering mechanism in migraine.

#### CTS and cancer.

There is an extensive literature on this topic, likely stimulated in part by two reports that, in heart failure patients with breast cancer, digitalis therapy was associated with significantly fewer tumor metastases and recurrences (638, 639). Some of the articles on breast cancer in patients on CTS therapy are contradictory, however. Another report claimed that CTS therapy was not associated with increased risk of breast cancer (640), but a meta-analysis of multiple studies concluded that, in women, CTS therapy was associated with a 34% increase in breast cancer risk (641). Nevertheless, several articles on breast cancer cell lines indicate that nanomolar concentrations of some CTS reduce cell proliferation, inhibit cell migration, and are cytotoxic, e.g., Refs. 642, 643; one suggested that these effects are due to inhibition of transport by NKA, and not to CTS receptor signaling (642). Another report showed that in vitro treatment of human breast cancer cells with 20 nM ouabain or digitoxin for 17 days delayed in vivo metastatic outgrowth when the cells were injected into the tail veins of mice. Also, when tumor cells were injected into the fat pads of mice and the tumors were permitted to grow for 14 wk, daily IP injection of ouabain for the next 3 wk markedly suppressed tumor metastatic activity but did not alter the primary tumor size or total number of circulating tumor cells (644).

Several retrospective reviews have indicated that men receiving digoxin therapy had a significantly reduced incidence of prostate cancer (645–648). Digoxin therapy was not, however, associated with reduced prostate cancer-specific mortality (648–651). In an in vitro investigation, the cardenolides, digoxin and lanatoside C, and the bufadienolide, proscillaridin A, all inhibited the growth of six prostate cancer cell lines with mean IC_50_’s = 163, 408, and 13 nM, respectively (645).

Many dozens of studies have exploited the cytotoxic action of ouabain, bufalin, and other CTS in an effort to kill a variety of types of cancer cells preferentially (for reviews, see Refs. 652–657). The vast majority of these reports describe in vitro studies of tumor cell lines, and do not directly compare the effects of CTS in tumor versus nontumor cells. Furthermore, the studies usually used high/toxic (generally >100–200 nM) concentrations of ouabain or other CTS (see Supplemental Fig. S9), overlooked the presence of low eCTS concentrations in media containing FBS, and ignored eCTS and their endocrine role in cell signaling. In a study of three human thyroid cell lines in culture, two derived from papillary cancers and one of nontumor lineage, for example, 100 nM ouabain decreased the number of viable cells and caused cell cycle arrest in all three lines (658). There are, however, a few exceptions to some of these generalities. In one study, 10–40 nM ouabain inhibited cell proliferation, elevated intracellular Ca^2+^ and ROS, and induced apoptosis of human renal cancer and small cell lung cancer cells; the effects were reportedly mediated by α3 NKA (659). In another study, treatment of cultured non-small cell lung cancer (NSCLC), cells with 2.5–40 pM ouabain suppressed several integrins, caused a “dramatic reduction of cell colony size” and inhibited cancer cell migration, perhaps an indication of an anti-metastatic effect (660). A third report described the ability of low nanomolar concentrations of ouabain to induce caspase-dependent apoptosis in leukemia stem cells although the LC_50_ ranged upward from ∼33 nM to >125 nM (661). A fourth investigation indicated that the anti-cancer effects of 0.02–2 µM ouabain, and 2 µM oleandrin and digoxin, may be the result of decreased expression of the PM glucose transporter, GLUT1, that is upregulated in many cancers to support the enhanced glycolysis (Warburg effect) (662). Those data, from human liver, colon, gastric, and oral cancer cell lines, suggest that the CTS interacted with α3 NKA to promote GLUT1 endocytosis via NAADP-mediated Ca^2+^ mobilization and activates dynamin, a GTPase that participates directly in the endocytotic process (663).

Numerous groups have examined the effects of bufalin and other bufadienolides as anticancer agents; for reviews, see Refs. 652, 655, 656. For example, bufalin (10–100 nM) inhibited human ovarian cancer cell proliferation in vitro by downregulating EGFR (664). Bufalin (100 nM) was synergistic with cisplatin in inhibiting cell growth and apoptosis and reversed cisplatin resistance by downregulating the PI3K/Akt pathway in gastric cancer cells (665). Bufalin, in vitro (50–100 nM), and in nude mice in vivo (dose not stated), inhibited the invasion and metastasis of human gastric cancer cells by promoting apoptosis and downregulating invasion-related gene expression (666). In mice with xenotransplanted human hepatocellular carcinoma, bufalin (10 µg/kg IP every 48 h) stimulated the anticancer immune response (667). A traditional Chinese medicine, cinobufatalin, a bufadienolide-containing toad skin extract, has been tested in a number of clinical trials. Two meta-analyses of multiple randomized controlled trials indicate that injection of cinobufatalin as an adjunct treatment in patients with NSCLC significantly improved 1- to 3-year survival rates and other outcome measures without additional adverse effects (668, 669).

Despite this extensive evidence that various CTS can be antitumor agents, one group recently made a compelling case that the EO-α1 NKA interaction contributes to human lung carcinoma aggression by promoting immune escape (123). Circulating EO (measured by LC-MS/MS) was elevated in patients with NSCLC and was highest in those with the most undifferentiated tumors, highest metastasis rates, and shortest survival times. Mice with immunologically competent Lewis lung cancer (LLC) also had higher plasma EO levels (ELISA) than mice in which the immune system was compromised, or circulating EO was immunoneutralized, antagonized with rostafuroxin or depleted by adrenalectomy. Ouabain administration (0.1 mg/kg, IP) to immune-competent LLC mice increased the expression of immunosuppresive marker genes and promoted tumor growth and metastasis; conversely, the immunosuppresive marker genes were downregulated in LLC mice treated with anti-ouabain antibodies. Ouabain (50 nM) also upregulated expression of programmed cell death protein ligand 1 (PD-L1) and negatively influenced tumor immunity in a human NSCLC cell line while anti-ouabain and anti-PD-L1 antibodies reversed the ouabain-induced upregulation of PD-L1. Silencing α1 NKA also increased PD-L1 expression by delaying its degradation. The anti-PD-L1 antibodies also reversed the ouabain-induced upregulation of immunosuppressive cytokines. PD-L1 interacted with, and may complex with, α1 NKA; this interaction led to the endocytosis and degradation of PD-L1. In human NSCLC tumors, PD-L1 expression correlated negatively with α1 NKA expression. Ouabain inhibited the α1/PD-L1 interaction and thereby enhanced PD-L1 expression; moreover, treatment with ouabain for more than an hour caused progressive downregulation of α1 NKA in NSCLC cells in vitro, which would also be expected to enhance PD-L1 expression.

Some of the aforementioned effects of CTS clearly appear to be contradictory. Consider, for example, the claims that EO suppresses the antitumor immunoresponse (123) but that bufalin stimulates this response (667). An alternative possibility, however, is that this might be another example of NKA ligand bias and ouabain-bufalin antagonism (61, 329). Consistent with the latter conjecture is the evidence that, while ouabain treatment of LLC mice increased tumor growth, injection of cinobufacini, a bufadienolide-containing toad skin extract, reduced tumor growth (123). Anti-PD-L1 antibodies inhibited tumor growth in these mice, and this effect was augmented by ouabain as well as by cinobufacini injection. These uncertainties and contradictions need to be resolved because of their potentially important implications for cancer therapy.

## SUMMARY AND CONCLUSIONS: THE ELUCIDATION OF A NOVEL ENDOCRINE SYSTEM

Cardenolide and bufadienolide CTS are synthesized by many plants, and it has long been known that certain amphibians also synthesize bufadienolides (670). These agents are toxic to many animals but certain insects, toads, snakes, and rodents developed strategies to employ the CTS for protection against predators, often, as exemplified by the monarch butterfly, using CTS-resistant mutations in their own NKA ouabain-binding sites. About 3,500 years ago, humans began to use plant extracts containing CTS preparations as therapeutics. In 1785, Withering first established the efficacy of digitalis extracts as a diuretic in patients with “dropsy” (and HF). Digitalis extract and some of its constituents, most notably, digoxin, then became the mainstay of HF therapy for over two centuries. In the 1950s and 1960s, we learned about the CTS receptor, the Na^+^ pump/NKA, and the mechanism, mediated by NCX, that underlies the acute cardiotonic action of CTS. It then all seemed straightforward: a cation pump that was the receptor for a large family of exogenous inhibitory molecules (56). Beginning in the early 1960s, and continuing to the present, however, seminal, largely unanticipated findings have repeatedly required revision of the then prevailing view of this remarkable enzyme whose complexity was so greatly underestimated. The following is a recap of these revelations which were reviewed in this article:

•  The first clues that something was amiss were the repeated reports, beginning in 1960, that subnanomolar concentrations of CTS sometimes stimulate the NKA (Table 2). We summarize the data and propose an explanation for that observation. That explanation also fits a more recent observation that antibodies raised against certain external surface epitopes on the NKA stimulate pump function.
•  The cloning and sequencing of the NKA, in 1985 (87) led to the discovery of the four mammalian NKA α (catalytic) subunit isoforms, with more than one isoform expressed in most cells. The isoforms have different properties, e.g., Na^+^, K^+^ and ouabain affinities, and regulation, and different PM localizations, implying that they have different functions. This is especially evident in rodent cells where α2^S^ or α3^S^ is coexpressed with α1^R^. Cloning also enabled the genetic engineering that permitted investigators to explore some of the complexities of NCX function.
•  After numerous hints and hypotheses about the existence of eCTS, the first such compound, EO, was purified and identified in 1991 (91). The surprise that EO was already a well-known, plant-derived toxin, ouabain, put some investigators off, and a few even continue to be skeptical despite the extensive confirming evidence. Importantly, there are at least three other eCTS, including MBG and an eDLC that have not yet been fully characterized, so we are surely in for more surprises.
•  Much early work had implicated an eCTS in the pathogenesis of hypertension, but proof of principle was required. Even so, a few investigators were incredulous of the direct demonstration (1993) that sustained ouabain treatment does, indeed, induce hypertension in rodents (94). A key early concern was the absence of evidence that digoxin induces hypertension despite its very wide use.
•  That concern was soon laid to rest, but another unanticipated property of CTS-NKA interaction was revealed when the sustained administration of ouabain and digoxin were directly compared in rodents (1992–93). Unlike ouabain, digoxin did not raise BP (327) but, even more surprisingly, it and its analogs blocked the hypertensinogenic effect of ouabain and its analogs (95, 96). As we have seen, that finding and the stimulation by low doses of ouabain require the NKA to function as tetraprotomers with quarter-site reactivity. Indeed, several groups have shown that the NKA forms stable, functional di- and tetraprotomers. The finding that antibodies raised against AA sequences in two NKA extracellular loops stimulate the NKA (111, 406) appears to confirm the view that much of the NKA in the PM is present as multiprotomers capable of dissociation and reassociation.
•  The next breakthrough, in 1996, further altered our concept of this cation pump. Askari and Xie showed that the NKA is not just an ion pump but is also a hormone receptor and signaling molecule (6, 102). This revolutionary endocrine role for the NKA is, itself, very complex and is still incompletely understood and underresearched in view of its widespread and profound functional implications.
•  While these advances were occurring, the DIG trial was under way to determine the clinical efficacy of digoxin therapy in HF. The results, reported in 1997, indicated that digoxin reduced hospitalization rate but not overall mortality (129). Although some small trials and reanalysis of the DIG trial data indicate that very low doses of digoxin may, in fact, be beneficial, effective new therapies have largely replaced the use of digoxin in HF, especially in the United States. Nevertheless, digoxin, largely because of its bradycardic effect, continues to be used for the treatment of atrial arrythmias. We have suggested, however, that the recent insight into CTS-NKA interactions ought to provide impetus for a reevaluation of low-dose digoxin as a therapy for HF (671).
•  Remarkable details of the structure of NKA with bound CTS have been elucidated during the past 15 years, but those results explain neither the mechanism by which NKA functions as a signaling molecule nor the effects of different CTS. This is an endocrine system with several distinct hormone ligands that have ligand-biased actions on the same receptor to induce different effects.
•  Ideas about a link between eCTS and hypertension, put forth in the 1960s and 1970s have now been verified in many rodent models. Furthermore, elevated plasma EO was detected in a large fraction of patients with essential hypertension or primary aldosteronism. Genetic engineering studies (knockout, overexpression, or alteration of α1 and α2 NKA ouabain sensitivity) and immunoneutralization or antagonism of the eCTS reveal that the α2^S^ NKA isoform and eCTS are crucial to the pathogenesis of hypertension in rodents. Genetic engineering in rodents also demonstrated that eCTS and the α2^S^ NKA are involved in the cardiac remodeling that is a manifestation of HF, complementing the observations that EO is elevated in both humans and rodents in HF.
•  Finally, a plethora of observations over the past 25 years demonstrate that CTS and, especially, ouabain/EO-activated, NKA-mediated signaling modulate an enormous variety of cell functions. These include cell viability, cell-cell communication, activation of the immune system, ROS generation, and inflammation. The signaling mechanisms also influence fetal development, exercise tolerance, vascular reactivity, salt balance, synapse development, and even behaviors such as depression and hyperactivity. Indeed, application of some of these findings might help to reduce the recurrence and spread of some cancers. In sum, the EO/NKA endocrine system has broad impact on physiology and pathophysiology, and therefore needs to be mainstreamed.

Our knowledge is still fragmentary, in part, because this system has been flying under the radar. The missing steps in the EO biosynthetic pathway (8) require elucidation, but this is no reason to ignore the hormone. Furthermore, as mentioned (*The CTS-Activated “Signalosome”*), the precise mechanism(s) responsible for EO-activated, PK cascade-mediated signaling are controversial and need to be resolved. For example, how does EO binding to NKA trigger these signals? And, precisely how does the EO-NKA interaction activate the PK cascades? Many studies on the EO-activated signaling pathways have been performed with high (toxic) concentrations of ouabain; these need to be examined with physiological EO levels (i.e., very low nanomolar and subnanomolar concentrations). This is especially important because EO concentrations within the “physiological and pathophysiological range” may have both beneficial and harmful effects. Moreover, different eCTS may have different (biased) effects. Finally, there is a need to isolate, structurally identify, and develop assay methods to determine the circulating concentrations and potencies of all the major human eCTS as a prelude to rigorous investigation of their interactions in vivo.

Even 70 years after the discovery of the Na^+^ pump and its interaction with CTS, there is still much to learn. It is exciting, nevertheless, that new as well as existing horizons for this highly influential cation active transporter/endocrine system continue to expand conceptually in multiple dimensions.

## SUPPLEMENTAL DATA

10.6084/m9.figshare.24849108 Supplemental Sections 1–3, Supplemental Tables S1–S3, and Supplementals Figs. S1–S11: 10.6084/m9.figshare.24849108.

## GRANTS

The research from our laboratories mentioned in this review was supported by research grants from the National Heart, Lung, and Blood Institute and the National Neurological Diseases and Stroke Institute of the National Institutes of Health, by the American Heart Association (AHA), and by an AHA Established Investigator Award (to J.M.H.).

## DISCLOSURES

The authors are unpaid consultants for a clinical trial supported by Serb Pharmaceuticals/BTG Pharmaceuticals. No conflicts of interest, financial or otherwise, are declared by the author.

## AUTHOR CONTRIBUTIONS

M.P.B. and J.M.H. analyzed data; interpreted results of experiments; prepared figures; drafted manuscript; edited and revised manuscript; and approved final version of manuscript.

## Footnotes

1 Cardenolide CTS are steroids with a five-member lactone; bufadienolide CTS are steroids with a six-member lactone ring (Fig. 1, *A* and *B*). In both types of steroids, the rings in the steroid core are typically cis-trans-cis-fused so that the A and D rings face one-another, and the lactone is attached to the core at C_17_.

2 The genus is also called *Scilla* or *Urginea*, and the plant is synonymous with sea onion or sea squill.

3 For clarity and uniformity, all NKA α subunit AA sequence numbering cited in this review is based on the sheep α1 NKA sequence numbering of Lingrel and Kuntzweiler (197).

4 Personal communication from Joseph F. Hoffman, who said that Post reported this exchange with Skou at the first meeting of the “Red Cell Club,” held at the National Institutes of Health in Bethesda, Maryland, on October 11, 1958.

5 We refer to these antibodies as “ouabain-selective” and not “ouabain-specific” because they are not truly “specific,” although they are often called “ouabain-specific” in the literature (e.g., Ref. 223). They all have some cross-reactivity and may be more or less selective depending upon the degree of reactivity with related molecules. This is especially important given the confusion about various antibodies used in the eCTS field.

6 As noted elsewhere (244), critical evidence was missing from the Baecher article (184). Specifically, following introduction of the internal standard into the sample plasma, it is appropriate/necessary to show that the internal standard can be detected in the extracted plasma in the LC-MS run. This approach is a cornerstone of all quantitative LC-MS and is used, in part, to compensate for variances in ion suppression that occur from sample to sample – but the extent of ion suppression was not shown by Baecher (184). Under sample conditions spiked with an internal standard and where the key molecular ions for EO are absent from an LC-MS-MS run while the internal standard is readily visible, it is entirely reasonable to conclude either that EO is not present or that it is below the lower limit of detection under the conditions used. Absent from the Baecher report (218), however, were the critical ion chromatograms necessary to demonstrate that the internal standard could be seen in the same plasma extract as that in which EO was absent/undetectable. Shown instead were ion chromatograms for the internal standard alone after extraction in the absence of plasma (i.e., in the absence of ion suppression), and ion chromatograms for the plasma extract without the internal standard (i.e., with heavy ion suppression). Most disturbing, the latter ion chromatograms had been deliberately altered to remove ion peaks (233) that, at a minimum, would have been consistent with isomers of EO. Crucially, depending to a large extent on the extraction method used, ion suppression can make the detection of EO difficult. This phenomenon could explain why they did not show the key ion chromatogram (218): heavy ion suppression in the plasma extract may have obscured both EO and the internal standard. Furthermore, because of the way their plasma extracts were prepared, the extent to which EO was lost during extraction cannot be readily determined due to the absence of the key ion suppression data.

7 For simplicity, and because no relevant information is available, we will assume that all the NKA di- and tetra-protomers are homo-oligomers with identical α and identical β subunit isoforms.

8 Personal communication from Robert Post to JMH at the 49^th^ Annual Society of General Physiologists Symposium/11^th^ ATPase Conference (Woods Hole, MA, September 6–11, 2005).

9 An important caveat concerns the controls for the antibody studies. In some cases, but not all, IgG was used as the antibody control, e.g., Supplemental Fig. S10 (100, 223, 109, 445); in no instances, however, were controls for the excipients in the antibody preparations included. Nevertheless, the antibody preparations were greatly diluted and the outcomes sufficiently robust so that the results were likely due to the immunoneutralization of the eCTS.

10 In practice, a very large range of concentrations of ouabain or other CTS has been employed, often, regrettably, in the toxic range. Orlov and colleagues have summarized the ouabain concentration ranges that activate and inhibit various cellular activities in nonrodent mammalian cells [Supplemental Fig. S9*A* (14)].

11 A 10 mM digoxin concentration in aqueous solution is unlikely because digoxin is very hydrophobic. Is this a typographic error and did the authors perhaps mean 10 μM?

12 DigiFab, like Digibind, is a commercial anti-digoxin Fab fragment preparation that is assumed to bind ouabain with high affinity.
